# Short Convergent Synthesis of the Mycolactone Core Through Lithiation–Borylation Homologations

**DOI:** 10.1002/chem.201503122

**Published:** 2015-09-01

**Authors:** Christopher A. Brown, Varinder K. Aggarwal

**Affiliations:** ^1^ School of Chemistry, University of Bristol, Cantock's Close, Bristol, BS8 1TS (UK)

**Keywords:** asymmetric synthesis, boron, iterative homologation, mycolactone, natural products

## Abstract

Using iterative lithiation–borylation homologations, the mycolactone toxin core has been synthesized in 13 steps and 17 % overall yield. The rapid build‐up of molecular complexity, high convergence and high stereoselectivity are noteworthy features of this synthesis.

The third most common *Mycobacterium* infection (after *M. tuberculosis* and *M. leprae*) is that of *M. ulcerans*, the pathogen responsible for the severe ulcerative skin disease, Buruli ulcer.^1^ Endemic in tropical Africa, it infects over 5000 patients per annum with 48 % of cases being aged under 15.1a, 2 Transmission is thought to occur by an aquatic organism bite,^3^ with initial manifestation occurring as a painless skin nodule. If diagnosed early, simple antibiotic chemotherapy is effective (80 %),^4^ however, if untreated, propagation of the infection results in large skin lesions of necrotic tissue and bone loss which are only treatable through aggressive surgery, resulting in scarring and loss of limb function.^2^, ^5^ The serious morbidity due to the socio‐economic burden of a young disabled workforce in rural communities^6^, ^7^ resulted in the World Health Organization identifying Buruli ulcer as one of seventeen neglected tropical diseases requiring research.^7^

*M. ulcerans* secretes a unique polyketide‐derived virulence factor, an equilibrating mixture of mycolactones A and B, **1** (Scheme 1, 3:2 *trans*/*cis*) which inhibits the immune response and causes necrosis of the infected tissue.1a, c Small and co‐workers^8^ successfully isolated milligram quantities of **1** allowing structure elucidation by NMR^9^ and confirmation of mycolactones A/B as the causative toxin through studies in vivo.^10^ A number of congers (C–F) have since been isolated containing the common lactone **2**, varying only by the appended acyl side chain.^11^, 1

**Scheme 1:**
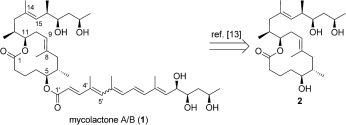
Structure of mycolactone A/B **1** and core **2**.

The absolute stereochemistry of firstly the lactone core **2** 12 and then mycolactones A/B **1** 13 was determined through total synthesis by Kishi and co‐workers. Multiple synthetic studies have since followed including a 3rd generation (1.3 g)^14^ synthesis of protected core **2** by Kishi, in addition to other accomplished syntheses by the groups of Negishi,^15^ Blanchard,^16^ and Altmann.^17^ These efforts have enabled further research into the pathogenesis of Buruli ulcer,^18^ aid the invention of new/simpler diagnostic techniques^19^ and allowed structural activity relationships of the core.^16^, ^17^, ^20^ These SAR investigations have shown that while the northern fragment can be augmented, a complete side chain is critical for the potency of **1**.20a The side chain of **1** has already been synthesized by the groups of Kishi,^21^ Negishi^22^ and Feringa/Minnaard,^23^ so we therefore focused our efforts towards the synthesis of the lactone core **2**. We were particularly keen on applying our recently developed lithiation–borylation methodology,^24^ which is highly effective in not only controlling stereochemistry but also simultaneously creating C—C bonds. Whilst such methodology has already been applied to a number of targets,^25^ including strategies involving iterative homologations for generating contiguous stereocenters,^26^ mycolactone core **2** represents a considerably higher level of complexity. Herein we describe our success in applying our lithiation–borylation methodology to a short convergent synthesis of this target molecule.

Our retrosynthetic analysis began with disconnection to the known intermediate **3** (Scheme 2).^12^, ^15^ We considered a lithiation–borylation disconnection between C11–C12 as this would lead to high convergency. Both boronic ester **4** and carbamate **5** could themselves be obtained through consecutive lithiation–borylation reactions of fragments **7** and **10**. Indeed, through our iterative methodology there was the prospect of coupling boronic ester **7** with building block **6** followed by carbamate **5** in one pot to give the lactone precursor **3**. Similarly carbamate **5** could be constructed in one pot from iterative coupling of boronic ester **10** and carbamates **9** and **6**.

**Scheme 2:**
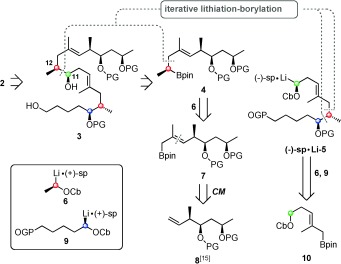
Retrosynthetic analysis of mycolactone core **2**. PG=Protecting Group, pin=pinacolato, Cb=*N*,*N‐*diisopropylcarbamoyl, sp=sparteine.

We began with the synthesis of boronic ester **10**, which was achieved in three high yielding steps (Scheme 3). Copper‐catalyzed formal hydroboration^27^ of alkynol **11** with B_2_pin_2_ in the presence of MeOH gave the desired vinyl boronate in 83 % yield as a single regio‐ and stereoisomer. Subsequent carbamoylation of the alcohol gave carbamate **12** in 87 % yield. Matteson one‐carbon homologation with chloromethyllithium **13** (formed in situ with **12**)^28^ gave the desired allylic boronic ester **10** as a 99:1 mixture with **12**. High conversion was achieved through the addition of precooled *n*BuLi^29^ and using an excess of the dihalide with respect to the organolithium to limit competing addition of *n*BuLi to boronic ester **12** and thereby favour lithium–halogen exchange. In contrast to vinyl boronic ester **12**, allylic boronic ester **10** was unstable to silica gel but was nevertheless obtained in high purity by simple filtration and evaporation.3

**Scheme 3:**
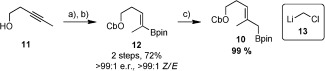
Synthesis of **10. a)** CuCl (5 mol %), PPh_3_ (6 mol %), KO*t*Bu (20 mol %), B_2_pin_2_, MeOH, THF, 83 %; **b)** CbCl, Et_3_N, THF, 87 %; **c)** BrCH_2_I *or* ClCH_2_I (3.5 equiv), *n*BuLi (2.4 equiv), Et_2_O (0.4 m), −78 °C *or* −95 °C, 99 %.

With boronic ester **10** in hand, our key lithiation–borylation reactions were examined (Scheme 4). The boronate complex, formed from the addition of **10** into **6** (1.5 equiv), underwent 1,2‐metallate rearrangement in refluxing Et_2_O to form **14** in 83 % yield. NMR analysis of the derived Mosher’s ester showed that the homologation occurred in 97:3 e.r. Subsequent reaction of **14** with **9** (1.3 equiv) proceeded well, providing alcohol **15** after oxidation in 77 %, >97:3 e.r. and 94:6 d.r. The diastereomeric ratio is consistent with reactions that are under full reagent control employing lithiated carbamates **6** and **9** with 97:3 e.r. Thus, carbamate **15** was formed in 63 % yield from **12** in three steps. These three steps could also be carried out sequentially, without intermediate purification (“one‐pot”), on identical scale in an increased 81 % yield without detriment to d.r. and further performed on an 8 mmol scale thereby delivering 3.0 g of **15**, with 85 % recovery of (+)‐sparteine. The potentially reactive carbamate group remained intact allowing us to circumvent functional group manipulation and, after C5 silyl protection (82 %), we obtained the desired carbamate **5** in 48 % over six steps from alcohol **11**.

**Scheme 4:**
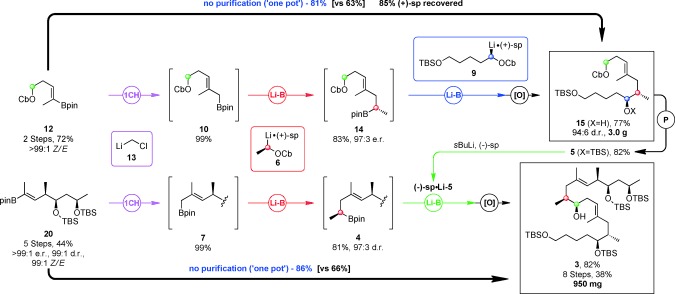
**1 CH** = ClCH_2_I (3.5 equiv), *n*BuLi (2.4 equiv), Et_2_O (0.4 m), −95 °C, formation of carbenoids**=**Carbamate (1.0 equiv), sparteine (1.0 equiv), *s*BuLi (1.0 equiv), Et_2_O (0.4 m), −78 °C, 5 h, **Li‐B=**Carbenoid (1.5 equiv), then RBpin (1.0 equiv), −78 °C, 2 h, then 40 °C, 16 h, **[O]=**NaOH/H_2_O_2_, THF, 0 °C, 2 h, **P=**TBSCl (1.4 equiv), imidazole (1.6 equiv), DMF, 25 °C, 16 h.

The synthesis of boronic ester **4** started with preparation of 7 g of alkene **8** through a four‐step known procedure in 74 % yield and 96:4 d.r. (Scheme 5).^15^, 17a Direct formation of allylic boronate **7** was investigated, employing methallyl boronic ester **17** and Hoveyda–Grubbs 2nd generation catalyst **19**, which gave **7** in 62 % yield but only as a 90:10 *E*/*Z* mixture of isomers. Olefin metathesis with vinylic boronic ester **18** has been reported to occur with much higher selectivity^30^ and was therefore explored. We were pleased to find that subjecting alkene **8** and vinylic boronic ester **18** to the identical cross metathesis conditions, yielded **20** as a single geometric isomer (0.4 mmol scale, 68 % yield). However, upon scale up we encountered two major problems: i) a dramatic reduction in conversion (10 % after 14 h, 3.8 mmol scale), and; ii) the formation of 1,2‐disubstituted alkene **21** (15 %). The latter observation has been described previously,^30^ possibly due to the transposition of boron from the internal to the terminal position of alkene **18** and subsequent metathesis with **8**. As this product was only observed by GC‐MS after extended reaction times (over 10 h), it was attributed to a transmuted catalyst of **19** causing the isomerization of **18**.^31^ Therefore, it was imperative to increase conversion over a short reaction time to avoid catalyst degradation. Through running the reaction at higher concentration (1.0 m) and adding the catalyst portion‐wise (5+5 mol %), we increased conversion to 45 % and reduced the amount of **21** formed (5 %). Finally, periodic degassing of the reaction every two hours removed the ethylene content of the solution and further pushed the equilibrium towards vinyl boronate **20**, achieving a 60 % yield on a 5.3 mmol scale over 10 h with minimal formation of alkene **21** (<5 %).5

**Scheme 5:**
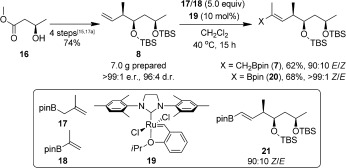
Olefin metathesis of **8**.

With our two key building blocks in hand, we examined our final iterative lithiation–borylation process. Matteson one‐carbon homologation of **20** proceeded in near quantitative yield and homologation of **7** with lithiated carbamate **6** (1.5 equiv) gave our required fragment **4** in 81 % isolated yield. Oxidation and NMR analysis showed it to be 97:3 d.r., consistent with the homologation of **6** with analogous allylic boronate **10**. For the final step, lithiation of **5** in the presence of (−)‐sparteine was required, but in explorative lithiation–deuteration experiments we isolated diene **22** 32 in 10 % yield, in addition to the required deuterated product (90 %). This showed that 10 % lithiation of **5** occurred at the allylic position followed by E_2_ elimination of the carbamate. We therefore used an excess of carbamate **5** (1.5 equiv) with respect to boronic ester **4** (1.0 equiv), and the final homologation and subsequent oxidation gave known intermediate **3** in 82 % yield and high d.r.^33^ with 950 mg prepared. With isolation and chromatographic purification of each intermediate, **3** was formed from **20** in 66 % over three steps. Once again, these three steps could also be carried out sequentially, without intermediate purification (“one‐pot”), in an increased 86 % yield. As a result, significant amounts (>900 mg) of **3** was obtained over eight steps from (*R*)‐3‐hydroxybutyrate in 38 % overall yield.

Completion of the synthesis followed literature precedent (Scheme 6).^12^, ^15^ Selective deprotection of the primary silyl ether with TBAF (85 %), followed by a two‐step TEMPO/Pinnick oxidation, yielded acid **23** in 81 %. Lactonisation of the 12‐membered core proceeded efficiently under Yamaguchi conditions (81 % yield), and subsequent global deprotection with HF⋅pyridine gave the mycolactone core **2** in 80 %. In forming the lactone ring, minor diastereomers observed in the formation of **3** were separated completing the synthesis of lactone **2**, which was identical in all respects to the literature, in a total of 13 steps and 17 % overall yield.6

**Scheme 6:**
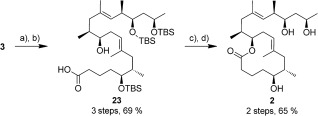
Completion of synthesis. a) TBAF, THF, 85 %; b) i. TEMPO (15 mol %), BAIB, CH_2_Cl_2_/H_2_O (2:1); then b) ii. NaClO_2_, 2‐methyl‐2‐butene, Na_2_H_2_PO_4_ buffer/*t*BuOH (2:1), 81 %; c) (C_6_H_2_Cl_3_)COCl, DMAP, PhH, 81 %; d) HF⋅pyridine, THF, 80 %.

In conclusion, the shortest synthesis of the mycolactone core to date has been completed both in terms of longest linear sequence (13 vs 14^14^ steps) and total step count (17 vs 28^14^ steps). Moreover if the sequenced iterative homologation is counted as one step, then the mycolactone core is achieved in only 11 steps. Although a scalable route has already been accomplished, our synthesis is able to rapidly deliver significant amounts (>100 mg) of highly enantio‐ and diastereoenriched mycolactone core through utilization of simple carbamate building blocks. Both in terms of step count and scale, the synthesis showcases the power of lithiation–borylation methodology for the efficient and convergent synthesis of complex molecules.

## Supporting information

As a service to our authors and readers, this journal provides supporting information supplied by the authors. Such materials are peer reviewed and may be re‐organized for online delivery, but are not copy‐edited or typeset. Technical support issues arising from supporting information (other than missing files) should be addressed to the authors.

miscellaneous_information
