# Supplementary material for: Short Convergent Synthesis of the Mycolactone Core Through Lithiation–Borylation Homologations
Source: Chemistry. 2015 Sep 1;21(40):13900–3. doi: 10.1002/chem.201503122 (PMC6519258; doi:10.1002/chem.201503122)
Supplement: Supplementary file 1 — miscellaneous_information [file CHEM-21-13900-s001.pdf]

CHEMISTRY
A European Journal

Supporting Information

Short Convergent Synthesis of the Mycolactone Core Through Lithiation–Borylation Homologations

Christopher A. Brown and Varinder K. Aggarwal*[a]

chem_201503122_sm_miscellaneous_information.pdf

Supplementary information

Short Convergent Synthesis of the Mycolactone Toxin Core Through Lithiation-Borylation Homologations

Christopher A. Brown and Varinder K. Aggarwal[1]

School of Chemistry, University of Bristol, Cantock's Close, Bristol BS8 1TS, UK.

1 General Information **2**
2 Materials and reagents **3**
3 General Procedures **3**
4 Synthesis of Carbamates and Reagents **5**
5 Synthesis of Carbamate 5 .... **9**
6 Synthesis Boronic Ester 4 .... **19**
7 Endgame **32**
8 ^{13}C Chemical Shift Comparison **36**
9 Determination of Stereochemical Purity **37**
10 ^{1}H and ^{13}C NMR Spectra **40**

1 General Information

All air- and water-sensitive reactions were carried out in oven-dried glassware under a N_2 atmosphere using standard Schlenk techniques. Analytical TLC was performed on aluminium backed plates pre-coated (0.25 mm) with Merck Silica Gel 60 F254. Compounds were stained using phosphomolybdic acid (PMA) or $KMnO_4$ followed by heating. Flash column chromatography was performed using Merck Silica Gel 60 (40-63 μm). All mixed solvent eluents are reported as *v/v* solutions.

^{1}H- and ^{13}C- Nuclear Magnetic Resonance (NMR) spectra were acquired at various field strengths as indicated using JEOL ECS 400, Varian 400 and Bruker Cryo 400 MHz spectrometers. ^{1}H spectra were referenced internally to the residual protio solvent resonance ($CHCl_3$ = 7.27 ppm, CH_3OH = 3.31 ppm). ^{13}C spectra were referenced internally to the residual deuterio solvent resonance ($CDCl_3$ = 77.16 ppm; CD_3OD = 49.15 ppm). ^{11}B spectra were referenced externally to $BF_3{\cdot}OEt_2$. ^{1}H and ^{13}C NMR coupling constants are reported in Hertz (Hz). Coupling constants are reported as follows: s = singlet, br. s = broad singlet, d = doublet, t = triplet, q = quartet, quin = quintet, sx = sextet, sept = septet, m = multiplet, dd = doublet of doublet, etc. Assignment of signals in ^{1}H- and ^{13}C-spectra was performed using ^{1}H-^{1}H COSY, DEPT, HMQC and HMBC experiments where appropriate. ^{13}C signals adjacent to boron are generally not observed due to quadrupolar relaxation. Impurity at 29.7 ppm in ^{13}C spectra is due to trace amounts of Apiezon high vacuum grease. Signal at 100.0 ppm is due to an artefact of JEOL.ECS 400 spectrometer at half scan width.

High resolution mass spectra were recorded using Electronic Ionization (EI), Electron Spray Ionization (ESI) or Chemical Ionization (CI). For CI, methane or NH_4OAc/MeOH was used. All Infra-Red (IR) data was obtained on a Perkin-Elmer Spectrum One FT-IR spectrometer. Optical rotations were obtained on a Perkin-Elmer 241MC polarimeter.

Names given to structures are those generated from ChemBioDraw 13.0 software (Perkin Elmer, 2014).

2 Materials and reagents

All reagents were used as received unless otherwise stated. Anhydrous Et_2O, PhMe and CH_2Cl_2 were dried using a purification column composed of activated alumina.[1] Anhydrous Et_2O, THF and CH_2Cl_2 were stored over 3 Å mol sieves. Benzene (PhH) was obtained by distilling over calcium hydride and subsequently stored over 3 Å mol sieves. Petroleum ether (PE) refers to the fraction collected between 40-60 °C. Where stated solvents and/or reagents were degassed using the freeze-pump-thaw method.[2] TMEDA, triethylamine and *N*,*N*-diisopropylethylamine (Hünig's base) were distilled over CaH_2 and stored in a Young's tube under N_2. Bromochloromethane and chloroiodomethane were distilled and stored in a Young's tube with a bead of copper as a stabilizer. (−)-Sparteine was obtained from the commercially available sulfate pentahydrate salt (ABCR chemicals) and isolated according to literature procedure.[3] (+)-Sparteine was obtained as the free base (BOC sciences), distilled over CaH_2 and stored in a Young's tube under N_2. The sparteine free base readily absorbs atmospheric carbon dioxide (CO_2) and should be stored in a Young's tube under argon/N_2 at −20 °C. Recovery of sparteine is described in the General Procedure 4 (**GP4**). Organolithiums were periodically titrated using *N*-benzylbenzamide.[4] Copper (I) chloride was purified by the addition of 37% HCl followed by the addition of water and filtration. Potassium *tert*-butoxide (KO*t*-Bu) was sublimed (192 °C, 0.8 mbar) and stored in a Schlenk tube under N_2 in a dessicator.

3 General Procedures

General Procedure 1 (GP1): Matteson One-Carbon Homologation of boronic esters

In a flamed dried flask under nitrogen a solution of vinyl boronate (1.0 equiv.) and chloroiodomethane (3.5 equiv.) in dry Et_2O (0.4 M) was cooled to −95 °C. *n*-Butyllithium (1.35 M in hexanes, 2.4 equiv.) was added slowly *via* a long needle partially submerged in the stirring solution. After the addition was complete the reaction mixture was allowed to warm slowly to room temperature and stirred for 10 minutes. The cloudy solution was filtered through a plug of silica and the filtrate concentrated under vacuum (1 mbar) to yield the homologated primary boronic ester (99%, <1% Starting material).

General Procedure 2 (GP2): Lithiation-borylation of boronic esters with primary *N,N*-diisopropylcarbamates

In an oven dried flask under nitrogen a stirring solution of primary carbamate (1.5 equiv.) and (+)-sparteine (1.5 equiv.) in Et_2O (0.4 M) was cooled to −78°C. To it was added *s*-butyllithium

(1.26 M in 92:8 cyclohexane:hexanes, 1.5 equiv.) dropwise and the resultant clear yellow solution was stirred for 5 h. The boronic ester (1.0 equiv.) in Et_2O (0.5 M) was added dropwise to the solution at −78°C and stirred for 2 h. The reaction mixture was slowly warmed to room temperature and the yellow solution was heated at 40 °C for 16 h and monitored by ^{11}B NMR until no borate complex remained. The reaction was diluted with Et_2O (15 mL) and aqueous HCl (2 M, 10 mL) and separated. The aqueous layer was extracted with Et_2O (2 × 10 mL) and the combined organics were washed with brine, dried over $MgSO_4$ and concentrated. The crude boronic esters were purified by flash column chromatography (SiO_2) to yield pure secondary boronic esters. Racemic analogues were obtained by exchanging (+)-sparteine with TMEDA.

General Procedure 3 (GP3): Oxidation of Boronic esters using NaOH/H_2O_2

Crude/purified boronic ester (0.2 mmol) was taken up in THF (2 mL) and cooled to 0 °C. To the vigorously stirring solution was added a mixture of 3 M NaOH with 1 g/l EDTA : 30% H_2O_2 (1:1, 2 mL) dropwise. The resultant solution was allowed to warm up to room temperature and monitored by TLC until no boronic ester remained (2 h). The solution was quenched slowly by adding saturated $Na_2S_2O_3$ (10 mL) dropwise. The crude solution was extracted with Et_2O (3 × 10 mL) and the organics combined, washed with brine (20 mL), dried with $MgSO_4$ and concentrated. The crude alcohols were purified by flash column chromatography (SiO_2) to obtain the pure alcohols.

General Procedure 4 (GP4): Recovery of sparteine[3]

The crude reaction mixture in Et_2O was extracted with 2 M HCl. The aqueous extracts were combined and made basic (to approximately pH 11) with saturated NaOH (20% *v/w*) and the aqueous layer extracted with Et_2O to obtain the sparteine free base. The organics were combined and dried over $MgSO_4$ and concentrated. Distillation of the free base over calcium hydride (CaH_2) gave sparteine as a colourless oil. (125-126 °C, 0.8 mbar)

1. A. B. Pangborn, M. A. Giardello, R. H. Grubbs, R. K. Rosen, F. J. Timmers, *Organometallics* **1996,** *15,* 1518
2. D. F. Shriver, M. A. Drezdzon, Wiley: New York **1986**, The manipulation of air-sensitive compounds 2nd ed
3. N. A. Nikolic, P. Beak, *Org. Synth* **1997,** *74,* 23
4. A. F. Burchat, J. M. Chong, N. Nielsen, *J. Organomet. Chem* **1997,** *542,* 281

4 Synthesis of Carbamates and Reagents

N,N-Diisopropyl ethylcarbamate, SI-1

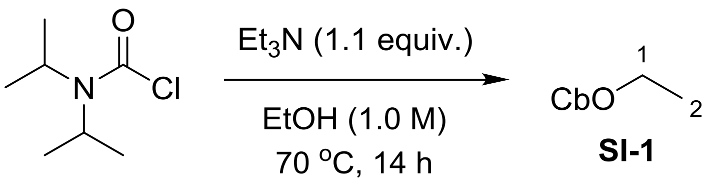

In a flame dried flask a solution of ethanol (100 mL, 1.00 M) and Et_3N (14.4 mL, 0.110 mol, 1.1 equiv.) was stirred and carbamoyl chloride (16.3 g, 0.100 mol, 1.0 equiv.) was added. The vessel was fitted with a reflux condenser and the reaction mixture was heated to 80 °C for 14 h. The reaction mixture was then cooled and diluted with water (150 mL), and pentane (100 mL). The organics were separated and washed with water (100 mL), brine (100 mL), dried over $MgSO_4$ and concentrated. The crude oil was purified by distillation under reduced pressure (37 °C bp, 20 mbar) to yield **SI-1** (15.6 g, 90%) as a colourless oil.

R_f (PE:Et_2O, 95:5)= 0.36

^{1}H NMR (400 MHz, $CDCl_3$): 4.12 (2H, q, J = 7.1 Hz, C^1H_2), 3.85 (2H, br. s, 2 × Cb NCH), 1.26 (3H, t, J = 7.1 Hz, C^2H_3), 1.19 (12H, d, J = 6.8 Hz, 4 × Cb CH_3)

^{13}C NMR (100 MHz, $CDCl_3$): 156.0 (C=O), 60.5 (C^2), 45.6 (2 × Cb NCH), 21.1 (4 × Cb CH_3), 14.8 (C^1)

Data in accordance with: J. L. Stymiest, G. Dutheuil, A. Mahmood, V. K. Aggarwal, *Angew. Chem. Int. Ed* **2007,** *119,* 7635

5-Hydroxypentyl-*N,N*-diisopropylcarbamate, SI-2

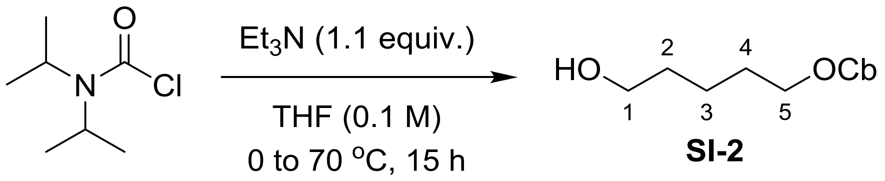

SI-2

A suspension of sodium hydride (1.44 g, 61.0 mmol, 1 equiv.) in THF (61 mL, 1.0 M) was cooled to 0 °C. To the suspension a solution of 1,5-pentanediol (19.0 mL, 183 mmol, 3 equiv.) in THF (61 mL) was added dropwise and the reaction mixture stirred for 1 h. A solution of carbamoyl chloride (10.0 g, 61.0 mmol, 1.0 equiv.) in THF (4 mL) was added and the reaction vessel subsequently fitted with a reflux condenser. The reaction mixture was heated at 70 °C for 14 h. The reaction was quenched with 2 M HCl (150 mL) and diluted with Et_2O (100 mL). The organics were separated and the aqueous layer extracted with Et_2O (2 × 80 mL). The combined organics were washed with saturated $NaHCO_3$ (100 mL), brine, dried over $MgSO_4$ and concentrated. The crude product was purified by flash column chromatography (SiO_2, PE:EtOAc, 50:50) to yield **SI-2** (12.8 g, 91%) as a colourless oil.

R_f (PE:EtOAc, 70:30)= 0.19

^{1}H NMR (400 MHz, $CDCl_3$): 4.08 (2H, t, J= 6.7 Hz, C^5H_2), 3.81 (2H, br. s, 2 × Cb NCH), 3.65 (2H, t, J = 6.5, C^1H_2), 1.72–1.66 (2H, m, C^4H_2), 1.65–1.58 (2H, m, C^2H_2), 1.49–1.41 (2H, m, C^3H_2), 1.20 (12H, d, J = 6.8 Hz, 4 × Cb CH_3)

^{13}C NMR (100 MHz, $CDCl_3$): 156.1 (C=O), 64.6 (C_5), 62.8 (C_1), 45.8 (2 × Cb NCH), 32.5 (C^4), 29.0 (C^2), 22.5 (C^3), 21.1 (4 × Cb CH_3)

IR v_{max} (neat)/cm^{-1}: 3436, 2935, 1668, 1291, 1065, 772

HMRS (ESI$^+$): calcd for $C_{12}H_{25}NO_3Na$ (M+Na): 254.1732, found 254.1739

5-((*tert*-Butyldimethylsilyl)oxy)pentyl-*N,N*-diisopropylcarbamate, SI-3

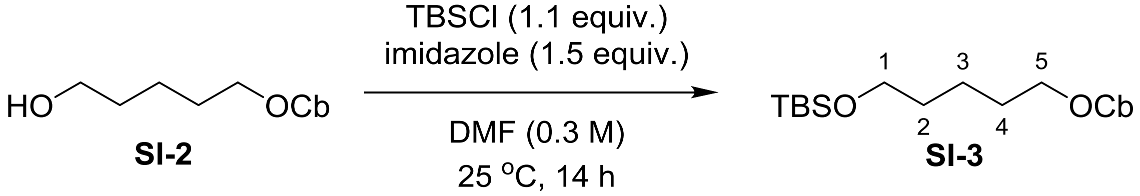

A solution of **SI-2** (9.40 g, 41.0 mmol) and imidazole (4.20 g, 61.0 mmol, 1.5 equiv.) in DMF (150 mL, 0.30 M) was cooled to 0 °C and to it was added *tert*-butyldimethylsilyl chloride (6.70 g, 45.0 mmol, 1.1 equiv.). The mixture was stirred at 25 °C for 14 h. Methanol (20 mL) was added and stirred for 10 minutes followed by 5% aqueous lithium chloride solution (150 mL) and Et_2O (100 mL). The layers were separated and the aqueous layer was extracted with more Et_2O (2 × 60 mL). The organics were combined and washed with brine, dried over $MgSO_4$ and concentrated. The crude product was purified by flash column chromatography (SiO_2, PE:EtOAc 96:4) to yield **SI-3** (13.6 g, 96%) as a colourless oil.

R_f (PE:EtOAc, 95:5)= 0.27

^{1}H NMR (400 MHz, CDCl$_3$): 4.08 (2H, t, J = 6.6 Hz, C^5H_2) , 3.83 (2H, br. s, 2 × Cb NCH), 3.62 (2H, t, J = 6.4 Hz, C^1H_2), 1.71–1.63 (2H, m, C^4H_2), 1.59–1.52 (2H, m, C^2H_2), 1.47–1.41 (2H, m, C^3H_2), 1.20 (12H, d, J = 6.8 Hz, 4 × Cb CH_3), 0.89 (9H, s, 3 × TBS CH_3), 0.04 (6H, s, 2 × $SiCH_3$)

^{13}C NMR (100 MHz, CDCl$_3$): 156.1 (C=O), 64.8 (C^5), 63.2 (C^1), 45.8 (2 × Cb NCH), 32.7 (C^2), 29.1 (C^4), 26.1 ($SiCMe_3$), 22.8 (C^3), 21.2 (4 × Cb CH_3), 18.5 (3 × TBS CH_3), −5.2 (2 × $SiCH_3$)

IR v_{max} (neat)/cm^{-1}: 2930, 1691, 1435, 1288, 1095, 772

HMRS (ESI$^+$): calcd for $C_{18}H_{39}NO_3SiNa$ (M+Na): 368.2597, found 368.2591

4,4,5,5-Tetramethyl-2-(2-methylallyl)-1,3,2-dioxaborolane, 17

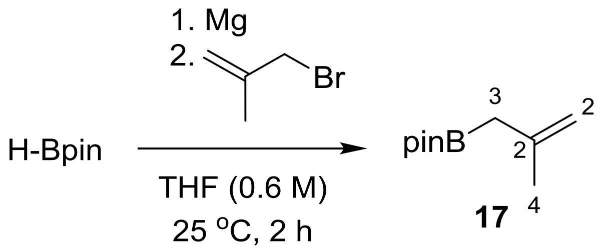

In a dry flask under nitrogen was added magnesium (722 mg, 29.7 mmol, 1.2 equiv.). Dry THF (41 mL, 0.60 M) and pinacolborane (2.30 mL, 24.8 mmol, 1.0 equiv.) were added and the mixture stirred at room temperature. To the mixture was added 3-bromo-2-methyl propene (2.50 mL, 25.0 mmol, 1.0 equiv.) dropwise. After 0.5 h of stirring, an additional 1.0 equivalent of allylic bromide was added. After the magnesium had been consumed (1.5 h) and ^{11}B NMR showed no remaining pinacolborane, the reaction was cooled to 0 °C, diluted with hexanes (80 mL) and, with care, aqueous HCl (150 mL, 0.1 M) was added (*caution: hydrogen evolution*). After 10 minutes the mixture was separated and the aqueous layer further extracted with hexane (2 × 80 mL). The combined organics were dried over $MgSO_4$, and concentrated, removing excess bromide under vacuum yielding **17** (2.90 g, 65%) as a colourless oil.

R_f (Hexanes:EtOAc 70:30)= 0.74

^{1}H NMR (400 MHz, $CDCl_3$): 4.65–4.69 (2H, m, C^1H_2), 1.77 (3H, s, C^4H_3), 1.72 (2H, s, C^3H_2), 1.25 (12H, s, 4 × pin CH_3)

^{13}C NMR (100 MHz, $CDCl_3$): 143.0 (C^2), 110.5 (C^1), 83.3 (2 × pin $C(Me)_2$), 24.7 (C^4), 24.4 (4 × pin CH_3)

Data in accordance with: J. W. Clary, T. J. Rettenmaier, R. Snelling, W. Bryks, J. Banwell, W. T. Wipke, B. Singaram, *J. Org. Chem.* **2011**, *76*, 9602

5 Synthesis of Carbamate 5

(*Z*)-4-(4,4,5,5-Tetramethyl-1,3,2-dioxaborolan-2-yl)pent-3-en-1-ol, SI-4

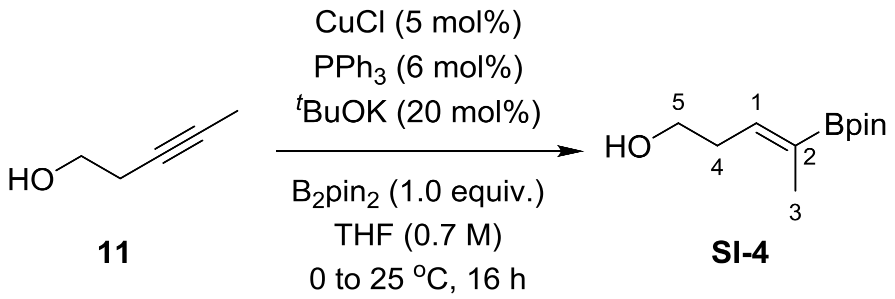

Under an atmosphere of nitrogen a solution of CuCl (336 mg, 2.50 mmol, 5 mol%), triphenylphosphine (787 mg, 3.00 mmol, 6 mol%) and KO*t*-Bu (1.12 g, 10.0 mmol, 20 mol%) in dry THF (25 mL) was stirred at 25 °C for 0.5 h. A solution of bis(pinacolato)diboron (14.0 g, 55.0 mmol, 1.1 equiv.) in THF (30 mL) was added and the resultant mixture stirred for 0.5 h. The reaction mixture was cooled to 0 °C and a solution of 3-pentyn-1-ol (4.60 mL, 50.0 mmol, 1.0 equiv.) and MeOH (4.00 mL, 100 mmol, 2.0 equiv.) in THF (15 mL) was added dropwise (*caution: vigorous exotherm*). After the addition was complete the reaction mixture was warmed to 25 °C and stirred for 16 h. The subsequent reaction mixture was filtered through celite with Et_2O and the solution concentrated. The residue was dissolved in THF (80 mL) and saturated $NaHCO_3$ solution (30 mL) and stirred at 25 °C for 4 h. EtOAc (100 mL) was added and the organics separated. The aqueous layer was washed with EtOAc (2 × 80 mL) and the organics were combined, washed with brine (100 mL), dried over $MgSO_4$ and concentrated. The alkyne was removed under high vacuum (<1 mbar). The crude product was purified by flash column chromatography (SiO_2, PE:EtOAc 85:15) to yield boronic ester **SI-4** (8.59 g, 81%) as a colourless oil.

R_f (PE:EtOAc, 60:40)= 0.29

^{1}H NMR (400 MHz, $CDCl_3$): 6.30 (1H, tq, J = 7.1, 1.8 Hz, C^1H), 3.71 (2H, t, J = 6.6 Hz, C^5H_2), 2.43 (2H, dtq, J = 7.1, 6.6, 0.9, C^4H_2), 1.72 (3H, s, C^3H_3), 1.44 (1H, br. s, C^5OH), 1.26 (12H, s, 4 × pin CH_3)

^{13}C NMR (100 MHz, $CDCl_3$): 141.5 (C^1), 83.4 (2 × pin $C(Me)_2$), 62.0 (C^5), 32.3 (C^4), 25.0 (4 × pin CH_3), 14.3 (C^3)

Data in accordance with: M. J. Hesse, C. P. Butts, C. L. Willis, V. K. Aggarwal, *Angew. Chem. Int. Ed.* **2012,** *51,* 12444

(Z)-4-(4,4,5,5-Tetramethyl-1,3,2-dioxaborolan-2-yl)pent-3-en-1-yl-*N,N*-diisopropylcarbamate, 12

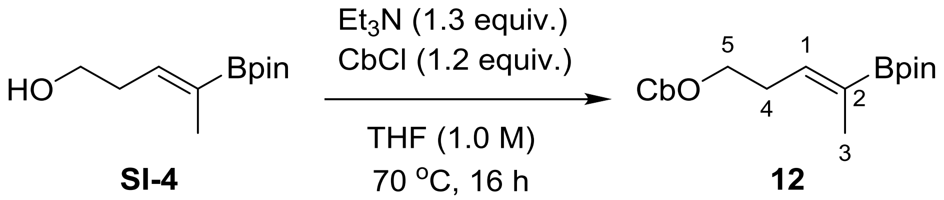

In a flame dried flask a solution of **SI-4** (8.41 g, 40.0 mmol, 1.0 equiv.), triethylamine (6.70 mL, 52.0 mmol, 1.3 equiv.) and carbamoyl chloride (7.70 g, 48.0 mmol, 1.2 equiv.) in THF (40 mL, 1.0 M) was heated to reflux for 16 h. The resultant reaction mixture was diluted with Et_2O (120 mL), filtered through a silica pad and the organics concentrated. The crude oil was purified by flash column chromatography (SiO_2, Hexanes:EtOAc, 88:12) to yield carbamate **12** (11.0 g, 84%) as a colourless oil.

R_f (PE:EtOAc, 85:15)= 0.27

^{1}H NMR (400 MHz, $CDCl_3$): 6.31 (1H, tq, J = 6.9, 1.7 Hz, C^1H), 4.12 (2H, t, J = 6.8 Hz, C^5H_2), 2.48 (2H, q, J = 6.8 Hz, C^4H_2), 1.69 (3H, m, C^3H_3), 1.23 (12H, s, 4 × pin CH_3), 1.17 (12H, d, J = 6.9 Hz, 4 × Cb CH_3)

^{13}C NMR (100 MHz, $CDCl_3$): 155.9 (C=O), 141.8 (C^1), 83.3 (2 × pin $C(Me)_2$), 63.7 (C^5), 45.8 (2 × Cb NCH), 28.7 (C^4), 24.9 (4 × pin CH_3), 21.2 (4 × Cb CH_3), 14.1 (C^3)

^{11}B NMR (96 MHz, $CDCl_3$): 29.9

IR v_{max} (neat)/cm^{-1}: 2974, 1668, 1368, 1133, 1067, 857

HMRS (ESI$^+$): calcd. for $C_{18}H_{34}BNO_4Na$ (M+Na): 362.2473, found 362.2460

(*E*)-4-Methyl-5-(4,4,5,5-tetramethyl-1,3,2-dioxaborolan-2-yl)pent-3-en-1-yl-*N,N*-diisopropylcarbamate, 10

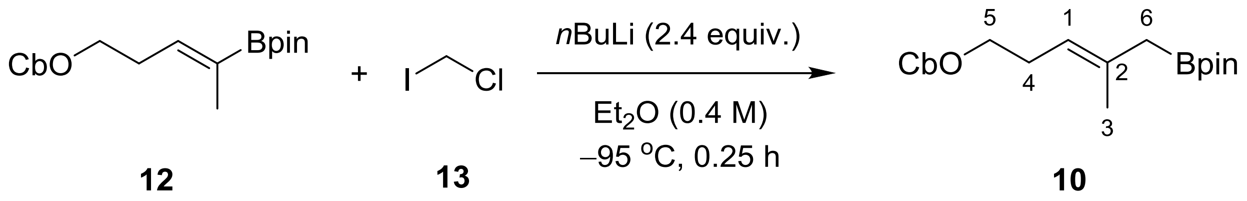

Following **GP1** with 8.0 mmol of vinyl boronic ester **12** yielded allylic boronic ester **10** (2.8 g, 99%) as a colourless oil.

R_f (PE:EtOAc, 80:20)= 0.47

^{1}H NMR (400 MHz, $CDCl_3$): 5.11 (1H, tq, J = 7.2, 1.2 Hz, C^1H), 4.02 (2H, t, J = 7.0 Hz, C^5H_2), 3.75 (2H, br. s, 2 × Cb NCH), 2.34 (2H, q, J = 7.1 Hz, C^4H_2), 1.69 (3H, s, C^3H_3) 1.67 (2H, s, C^6H_2), 1.23 (12H, s, 4 × pin CH_3), 1.19 (12H, d, J = 6.9 Hz, 4 × Cb CH_3)

^{13}C NMR (100 MHz, $CDCl_3$): 156.2 (C=O), 134.7 (C^2), 120.1 (C^1), 83.4 (2 × pin $C(Me)_2$), 64.7 (C^5), 46.3 (2 × Cb NCH), 28.6 (C^4), 25.0 (4 × pin CH_3), 21.3 (4 × Cb CH_3), 18.4 (C^3)

^{11}B NMR (96 MHz, $CDCl_3$): 29.4

IR ν_{max} (neat)/cm^{-1}: 2929, 1472, 1253, 1088, 831, 771

HMRS (ESI$^+$): calcd. for $C_{19}H_{36}BNO_4Na$ (M+Na): 376.2629, found 376.2625

Data in accordance with: S. Roesner, C. A. Brown, M. Mohiti, A. P. Pulis, R. Rasappan, D. J. Blair, S. Essafi, D. Leonori, V. K. Aggarwal, *Chem Commun.* **2014,** *50,* 4053

(*S,E*)-4-Methyl-6-(4,4,5,5-tetramethyl-1,3,2-dioxaborolan-2-yl)hept-3-en-1-yl-*N,N*-diisopropylcarbamate, 14

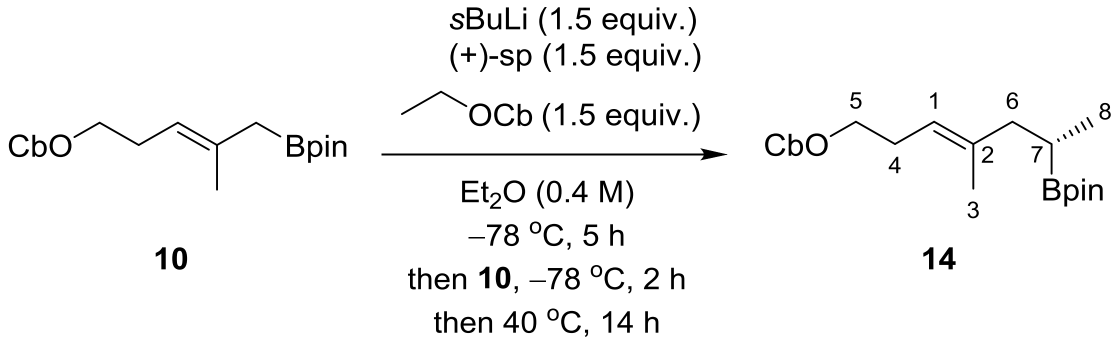

10 **14**

Following **GP2** on a 1.40 mmol scale of boronate **10** with 12 h reflux and the crude oil was purified by flash column chromatography (SiO_2, 4 × 15 cm PE:EtOAc, 90:10) to yield secondary boronic ester **14** (450 mg, 83%) as a colourless oil.

R_f (PE:EtOAc, 85:15)= 0.31

^{1}H NMR (400 MHz, $CDCl_3$): 5.15 (1H, t, J = 6.9 Hz, C^1H), 4.04 (2H, t, J = 6.9 Hz, C^5H_2), 3.78 (2H, br. s, 2 × Cb NCH), 2.34 (2H, m, C^4H_2), 2.19 (1H, dd, J= 13.7, 7.0 Hz, C^6H), 1.90 (1H, dd, J = 13.7, 8.6 Hz, C^6H), 1.60 (3H, s, C^3H_3), 1.22 (12 H, s, 4 × pin CH_3), 1.22 (1H, m, C^7H), 1.19 (12H, d, J = 6.9 Hz, 4 × Cb CH_3), 0.88 (3H, d, J = 7.4 Hz, C^8H_3)

^{13}C NMR (100 MHz, $CDCl_3$): 156.0 (C=O), 137.3 (C^2), 120.7 (C^1), 83.0 (2 × pin $C(Me)_2$), 64.5 (C^5), 46.5 (2 × Cb NCH), 42.9 (C^6), 28.2 (C^4), 24.9 (4 × pin CH_3), 21.2 (4 × Cb CH_3), 16.0 (C^3), 15.0 (C^8)

^{11}B NMR (96 MHz, $CDCl_3$): 32.6

$[\alpha]_D^{22.4}$ **=** −5.8 (c = 0.69, $CHCl_3$)

IR ν_{max} (neat)/cm^{-1}: 2927, 1690, 1369, 1310, 1068, 735

HMRS (ESI$^+$): calcd. for $C_{21}H_{40}NO_4Na$ (M+Na): 404.2946, found 404.2938

(*S,E*)-6-Hydroxy-4-methylhept-3-en-1-yl-*N,N*-diisopropylcarbamate, SI-5

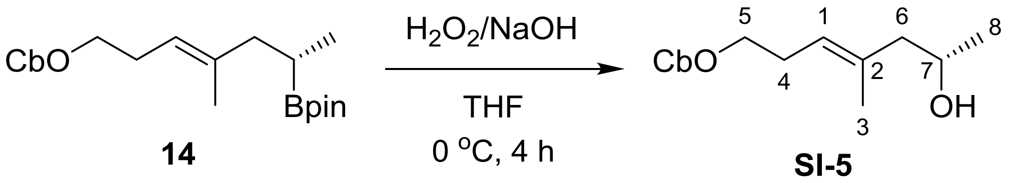

Following **GP3** on a 0.20 mmol scale of boronic ester **14** the crude oil was purified by flash column chromatography (SiO_2, 2 × 15 cm PE:EtOAc, 70:30) to yield secondary alcohol **SI-5** (50 mg, 88%) as a colourless oil.

R_f (PE:EtOAc, 70:30)= 0.32

^{1}H NMR (400 MHz, $CDCl_3$): 5.28 (1H, tq, J = 7.2, 1.0 Hz, C^1H), 4.11 (2H, t, J = 6.5 Hz, C^5H_2), 3.88 (1H, dqd, J = 8.7, 6.1, 4.0 Hz, C^7H), 3.76 (2H, br. s, 2 × Cb NCH), 2.39 (2H, m, C^4H_2), 2.15 (1H, dd, J = 13.4, 4.0 Hz, C^6H), 2.07 (1H, dd, J = 13.4, 8.9 Hz, C^6H), 1.89 (1H, br. s, C^7OH), 1.66 (3H, s, C^3H_3), 1.19 – 1.17 (15H, 2 × d, J = 6.9 Hz, 4 × Cb CH_3 and C^8H_3)

^{13}C NMR (100 MHz, $CDCl_3$): 156.0 (C=O), 134.7 (C^2), 124.2 (C^1), 65.0 (C^7), 64.2 (C^5), 50.0 (C^6), 45.9 (2 × Cb NCH), 28.4 (C^4), 23.0 (C^8), 21.2 (4 × Cb CH_3), 16.5 (C^3)

IR v_{max} (neat)/cm^{-1}: 2927, 1690, 1369, 1310, 1068, 735

$[\alpha]_D^{22.4}$ **=** +5.2 (c = 1.2, $CHCl_3$)

HMRS (ESI$^+$): calcd. for $C_{15}H_{29}NO_3Na$ (M+Na): 294.2040, found 294.2041

(*S,E*)-7-((*N,N*-Diisopropylcarbamoyl)oxy)-4-methylhept-4-en-2-yl (*S*)-3,3,3-trifluoro-2-methoxy-2-phenylpropanoate, SI-6

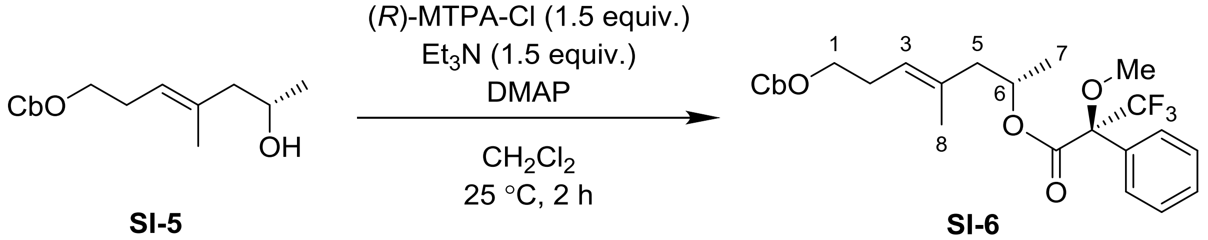

A solution of alcohol **SI-5** (19 mg, 0.069 mmol, 1.0 equiv) and triethylamine (15µL, 0.10 mmol, 1.5 equiv.) in CH_2Cl_2 (0.6 mL, 0.1 M) was added (*R*)-MTPA-Cl (19 µL, 0.10 mmol, 1.5 equiv.) and a catalytic amount of DMAP (1 mg) and stirred at 25 °C for two hours. Once complete, the reaction mixture was diluted with CH_2Cl_2 (5 mL) and water (5 mL) and the organics separated. The organics were washed with brine, dried over $MgSO_4$ and concentrated. The crude product was purified by flash column chromatography (SiO_2, PE:EtOAc 90:10) to yield ester **SI-6** (11 mg, 23%, 97:3 *dr*) as a colourless oil.

R_f (PE:EtOAc, 90:10)= 0.31

^{1}H NMR (500 MHz, $CDCl_3$): 7.54 – 7.50 (2H, m, ArH), 7.41 – 7.37 (3H, ArH), 5.31 (1H, dqd *J* = 7.4, 7.2, 6.4 Hz, C^6H), 5.26 (1H, t, *J* = 7.2 Hz, C^3H), 4.05 (2H, t, *J* = 6.8 Hz, C^1H_2), 3.81 (br. s, 2 × Cb NCH), 3.54 (3H, s, MeO), 2.42 (2H, x, *J* = Hz, C^2H_2), 2.31 (1H, dd, *J* = 14.1, 7.1 Hz, C^5H), 2.22 (1H, dd, *J* = 14.1, 6.5 Hz, C^5H) 1.68 (3H, s, C^8H_3), 1.24 (3H, d, *J* = 6.3 Hz, C^7H_3), 1.20 (12H, d, *J* = 6.8 Hz, 4 × Cb CH_3)

^{13}C NMR (125 MHz, $CDCl_3$): 166.2 (CO_2R), 155.9 (Cb C=O), 133.0 (ArC), 132.5 (ArC), 129.6 (C^4), 128.5 (C^3), 127.5 (ArC), 124.5 (ArC), 123.5 (q, $^1J_{C-F}$=290 Hz, CF_3), 84.7 (q, $^2J_{C-F}$= 28 Hz, C-CF_3), 72.3 (C^6), 64.1 (C^1), 55.5 (COMe), 46.1(C^5), 45.8 (2 × Cb NCH), 28.2 (C^2), 21.2 (4 × Cb CH_3), 19.5 (C^7), 16.4 (C^8)

^{19}F NMR (400 MHz, $CDCl_3$): -71.34, Minor diastereomer at -71.18

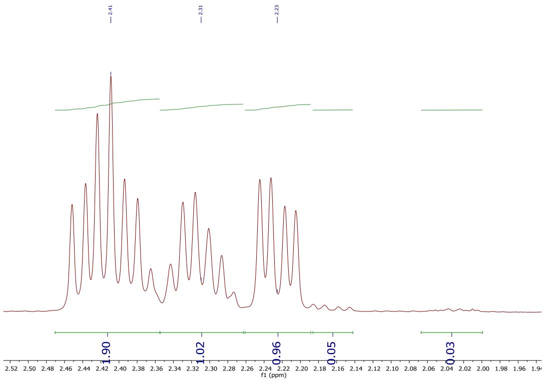

(*6S,7S,E*)-11-((*tert*-Butyldimethylsilyl)oxy)-7-hydroxy-4,6-dimethylundec-3-en-1-yl-*N,N*-diisopropylcarbamate, 15

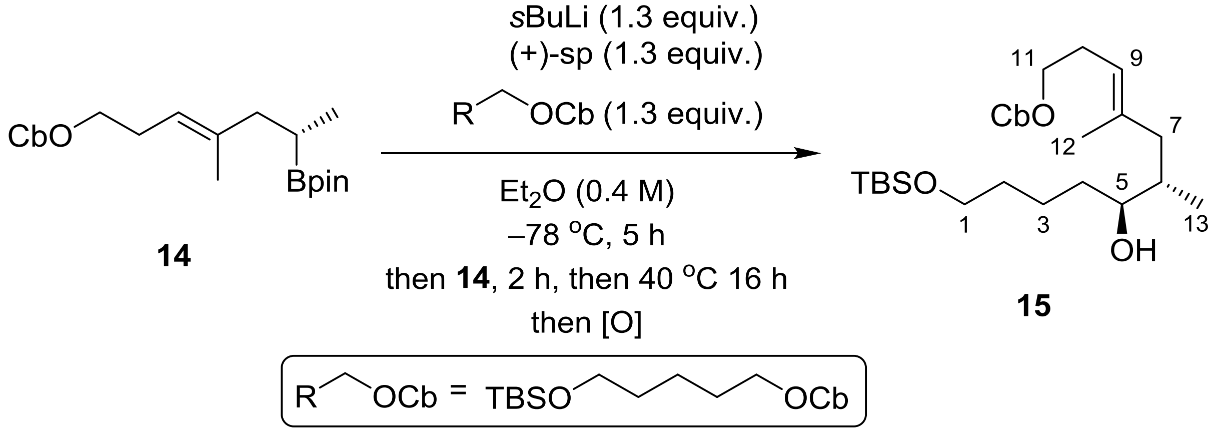

Following **GP2** on a 1.0 mmol scale with secondary boronic ester **14** with 12 h reflux. Following **GP3** the crude oil was oxidised and purified by flash column chromatography (SiO_2, 2 × 15 cm PE:EtOAc, 90:10) to yield alcohol **15** (360 mg, 77%) as a colourless oil.

R_f (PE:EtOAc, 70:30)= 0.39

^{1}H NMR (400 MHz, $CDCl_3$): 5.19 (1H, t, J = 7.1 Hz, C^9H), 4.07 (2H, t, J = 6.8 Hz, $C^{11}H_2$), 3.79 (2H, br. s, 2 × Cb NCH), 3.62 (2H, t, J = 6.3 Hz, C^1H_2), 3.47, (1H, m, C^5H), 2.36 (2H, app q, J = 6.9 Hz, $C^{10}H_2$), 2.13 (1H, dd, J = 13.1, 6.1 Hz, C^7H), 1.86 (1H, dd, J = 13.1, 8.7 Hz, C^7H), 1.68 (1H, m, C^6H), 1.61 (3H, s, $C^{12}H_3$), 1.57 – 1.50 (2H, m, C^3H_2), 1.48 – 1.41 (3H, m, C^2H_2, C^4H), 1.35 (1H, m, C^4H), 1.19 (12H, d, J = 6.9 Hz, 4 × Cb CH_3), 0.89 (9H, s, 3 × TBS CH_3), 0.79 (3H, d, J = 6.8 Hz, $C^{13}H_3$), 0.05 (3H, s, TBS CH_3), 0.04 (3H, s, TBS CH_3)

^{13}C NMR (100 MHz, $CDCl_3$): 156.0 (C=O), 136.2 (C^8), 122.2 (C^9), 74.6 (C^5), 64.4 (C^{11}), 63.3 (C^1), 45.9 (2 × Cb NCH), 44.1 (C^7), 35.8 (C^6), 34.4 (C^2), 33.0 (C^3), 28.2 (C^{10}), 26.1 ($SiCMe_3$), 22.8 (C^4), 21.1 (4 × Cb CH_3), 18.5 (3 × TBS CH_3), 16.1 (C^{12}), 13.2 (C^{13}), −5.1 (2 × $SiCH_3$).

IR v_{max} (neat)/cm^{-1}: 3474, 2930, 1674, 1296, 1097, 834, 773

$[\alpha]_D^{21.4}$ **=** −5.4 (c = 0.92, $CHCl_3$)

HMRS (ESI$^+$): calcd. for $C_{26}H_{53}NO_4SiNa$ (M+Na): 494.3636, found 494.3631

Process from 12 to 15 without Chromatographic Purification

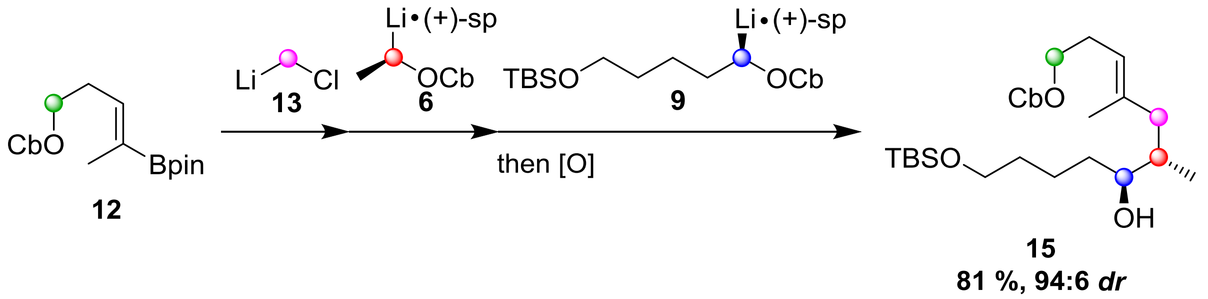

Following **GP1** with 7.9 mmol (2.7 g) of vinyl boronic ester **12** yielded allylic boronic ester **10** (2.8 g, 99%) as a colourless oil. This was used directly in the next step.

A solution of **10** (2.8 g, 7.9 mmol, 1.0 equiv.) in Et_2O (20 mL) was added into a solution of lithiated carbamate **6** (12 mmol, 1.5 equiv., synthesised following **GP2**) dropwise and stirred for 2 h at −78 °C. The reaction mixture was slowly warmed to room temperature and subsequently heated to 40 °C for 16 h. The resultant solution was taken up in a syringe and the residual solid was further washed with additional dry Et_2O (10 mL).

The crude solution of **14** was added into a solution of lithiated carbamate **9** (10.3 mmol, 1.3 equiv., synthesised following **GP2**) dropwise and stirred for 2 h at −78 °C. The reaction mixture was slowly warmed to room temperature and subsequently heated to 40 °C for 16 h. The reaction mixture was concentrated *in vacuo.*

The crude reaction mixture was taken up in THF (30 mL), cooled to 0 °C and oxidised following **GP3** with 3M NaOH (containing 1 g/l EDTA) : 30% H_2O_2 (1:1 *v/v*, 30 mL).

(+)-sparteine was recovered from the resultant crude mixture by following **GP4** and, after distillation, 4.3 mL was recovered (85% recovered from both steps).

The crude alcohol was purified via flash column chromatography (SiO_2, PE:EtOAc, 90:10 to 85:15) to yield secondary alcohol **15** (3.0 g, 81%, 94:6 *dr*) as a colourless oil.

(*6S,7S,E*)-7,11-Bis((*tert*-butyldimethylsilyl)oxy)-4,6-dimethylundec-3-en-1-yl-*N,N*-diisopropylcarbamate, 5

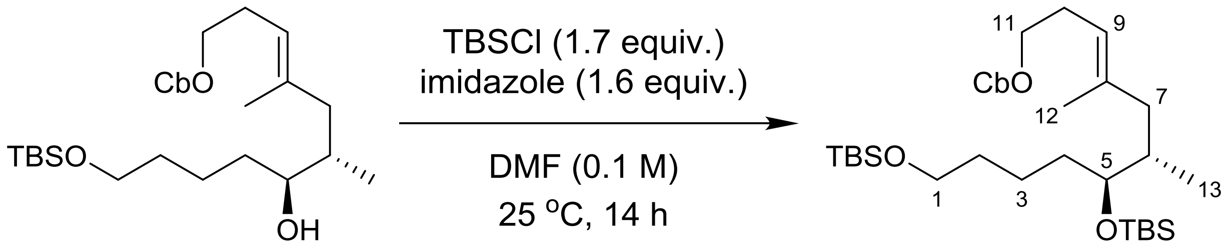

15 **5**

A solution of **15** (3.0 g, 6.4 mmol) and imidazole (680 mg, 10 mmol,1.6 equiv) in DMF (64 mL, 0.1 M) was stirred at 25 °C and to it was added *tert*-butyldimethylsilyl chloride (1.4 g, 9.6 mmol, 1.5 equiv.) and the solution was stirred at 25 °C for 12 h. The reaction was quenched with 5% aqueous lithium chloride solution (120 mL) followed by Et_2O (150 mL). The layers were separated and the aqueous layer was extracted twice more with Et_2O (2 × 100 mL). The organics were combined and washed with brine (200 mL), dried over $MgSO_4$ and concentrated. The crude product was purified by flash column chromatography (SiO_2 5 × 16 cm, PE:Et_2O, 94:6) to yield **5** (3.0 g, 82%) as a colourless oil.

R_f (PE:EtOAc, 85:15)= 0.67

^{1}H NMR (500 MHz, $CDCl_3$): 5.15 (1H, t, J= 7.1 Hz, C^9H), 4.07 (2H, t, J = 6.9 Hz, $C^{11}H_2$), 3.82 (2H, br. s, 2 × Cb NCH), 3.61 (2H, t, J = 6.9 Hz, C^1H_2), 3.51 (1H, m, C^5H), 2.37 (2H, q, J = 6.9 Hz, $C^{10}H_2$), 2.18 (1H, app q, J = 9.5 Hz, C^7H), 1.76 – 1.68 (2H, m), 1.59 (3H, s, C^3H_3), 1.54 – 1.48 (2H, m), 1.44 – 1.35 (3H, m), 1.26 (1H, m), 1.20 (12H, d, J = 6.8 Hz, 4 × Cb CH_3), 0.90 (9H, s, 3 × TBS CH_3), 0.90 (9H, s, 3 × TBS CH_3), 0.73 (3H, d, J= 6.3 Hz, $C^{13}H_3$), 0.05 (6H, s 2 × $SiCH_3$), 0.04 (6H, s, 2 × $SiCH_3$)

^{13}C NMR (125 MHz, $CDCl_3$): 156.0 (C=O), 136.7 (C^8), 121.6 (C^9), 76.1 (C^5), 64.5 (C^{11}), 63.3 (C^1), 45.7 (2 × Cb NCH), 42.9 (C^7), 35.7 (C^6), 33.4 (C^2), 33.3 (C^3), 28.2 (C^{10}), 26.1 (2 × $SiCMe_3$), 22.5 (C^4), 21.2 (4 × Cb CH_3), 18.5 (3 × TBS CH_3), 18.3 (3 × TBS CH_3), 16.1 (C^{12}), 13.9 (C^{13}), −4.0 ($SiCH_3$), −4.2 ($SiCH_3$), −5.1 (2 × $SiCH_3$)

IR v_{max} (neat)/cm^{-1}: 2930, 1694, 1290, 1069, 833, 771

$[\alpha]_D^{21.4}$ **=** −4.6 (c = 1.1, $CHCl_3$)

HMRS (ESI$^+$): calcd. for $C_{32}H_{67}NO_4Si_2Na$ (M+Na): 608.4501, found 608.4495

6 Synthesis Boronic Ester 4

Methyl-(*R*)-3-((*tert*-butyldimethylsilyl)oxy)butanoate, SI-7

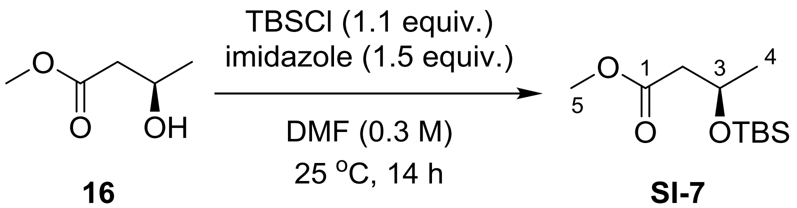

To a solution of methyl 3-(*R*)-hydroxybutyrate **(16)** (13.5 g, 114 mmol) in DMF (115 mL, 1.0 M) were added imidazole (15.5 g, 228 mmol, 2.0 equiv.) and *tert*-butyldimethylsilyl chloride (20.7 g, 137 mmol, 1.2 equiv.) and the mixture stirred for 20 h. The reaction mixture was diluted with Et_2O (150 mL) and 2 M HCl (80 mL) and the organics were separated. The aqueous layer was extracted with Et_2O (2 × 50 mL) and the combined organics were washed with saturated $NaHCO_3$ solution (75 mL), brine (50 mL) and dried over $MgSO_4$ and concentrated. The crude product was purified by flash column chromatography (SiO_2, PE:Et_2O, 97:3) to give **SI-7** (6.90 g, 99%) as a colourless oil.

R_f (PE:EtOAc, 95:5)= 0.42

^{1}H NMR (400 MHz, $CDCl_3$): 4.27 (1H, ddq, J = 5.3, 7.7, 6.1 Hz, C^3H), 3.66 (3H, s, C^5H_3), 2.48 (1H, dd, J = 7.7, 14.5 Hz, C^2H_2), 2.37 (H, dd, J = 7.7, 14.5 Hz, C^2H_2), 1.19 (3H, d, J = 6.1 Hz, C^4H_3), 0.86 (9H, s, 3 × TBS CH_3), 0.06 (3H, s, $SiCH_3$), 0.03 (3H, s, $SiCH_3$)

^{13}C NMR (100 MHz, $CDCl_3$): 172.1 (C^1), 65.8 (C^3), 51.4 (C^2), 44.7 (C^5), 25.7 ($SiCMe_3$), 23.9 (C^4), 17.9 (3 × TBS CH_3), −4.5 ($SiCH_3$), −5.1 ($SiCH_3$)

$[\alpha]_D^{20.6}$ = −17 (c = 0.92, $CHCl_3$)

Data in accordance with: H. Ishiyama, M. Ishibashi, A Ogawa, S. Yoshida, J.-i Kobayashi, *J. Org. Chem.* **1997,** *62,* 3831, **and**, D. S. Coffey, A. I. McDonald, L. E. Overman, M. H. Rabinowitz, P. A. Renhowe, *J. Am. Chem. Soc.* **2000,** *122,* 4893

(*R*)-3-((*tert*-Butyldimethylsilyl)oxy)butanal, SI-8

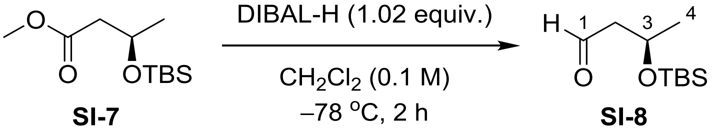

A solution of **SI-7** (9.3 g, 40 mmol) in CH_2Cl_2 (400 mL, 0.1 M) was cooled to −78 °C and to it was added dropwise DIBAL-H (41 mL, 1 M in hexanes, 41 mmol, 1.02 equiv.). After being stirred at −78 °C for 1 h, the reaction mixture was diluted with Et_2O (30 mL) quenched slowly with water (1.64 mL), then NaOH solution (1.64 mL, 15% *wt*). After warming to room temperature and stirring for 15 minutes, water (4.1 mL) was added. After 10 minutes stirring $MgSO_4$ was added and the solution was filtered through celite and concentrated. The crude material was purified by flash column chromatography (SiO_2, PE:EtOAc 98.5:1.5) to give **SI-8** (7.7 g, 93%) as a colourless oil.

R_f (PE:EtOAc, 97:3)= 0.3

^{1}H NMR (400 MHz, $CDCl_3$): 9.79 (1H, dd, J = 2.1, 2.7 Hz, C^1HO), 4.35 (1H, dqd, J = 5.1, 6.2, 6.4 Hz, C^3H), 2.55 (1H, ddd, J = 2.9, 7.0, 15.6 Hz, C^2H_2), 2.46 (1H, ddd, 2.1, 5.0, 15.6 Hz, C^2H_2), 1.24 (3H, d, J = 6.15 Hz, C^4H_3), 0.87 (9H, s, TBS), 0.07 (3H, s, $SiCH_3$), 0.06 (3H, s, $SiCH_3$)

^{13}C NMR ($CDCl_3$, 100 MHz): 202.4 (C^1), 64.7 (C^3), 53.1 (C^2), 25.9 ($SiCMe_3$), 24.3 (C^4), 18.1 (3 × TBS CH_3), −4.2 ($SiCH_3$), −4.8 ($SiCH_3$)

$[\alpha]_D^{20.9}$ = +2.0 (c = 1.0, $CHCl_3$)

Data in accordance with: G. E. Keck, A. Palani, S. F. McHardy, *J. Org. Chem.* **1994**, *59*, 3113

(*3R,4R,6R*)-6-((*tert*-Butyldimethylsilyl)oxy)-3-methylhept-1-en-4-ol, SI-9

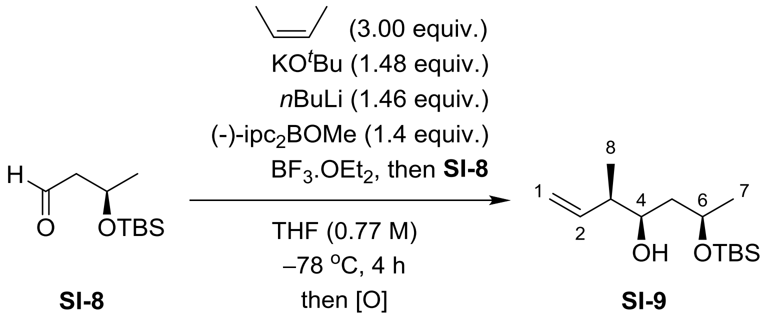

To a stirred solution of KO*t*-Bu (200 mL, 0.14 M in THF, 40 mmol, 1.48 equiv.) and (*Z*)-2-butene (7.0 mL, 80 mmol, 3.0 equiv.) was added dropwise *n*-butyllithium (25 mL, 1.60 M in hexanes, 39 mmol, 1.46 equiv.) at −78 °C. The bright yellow suspension was stirred at −78 °C for 15 minutes, warmed to −42 °C for 20 minutes and returned to −78 °C. (-)-*B*-methoxydiisopinocamphenyl borane (13 g, 38 mmol, 1.4 equiv.) in Et_2O (20 mL, 1.9 M) was added dropwise and stirred for 1 h at −78 °C. Boron trifluoride etherate (6.3 mL, 51 mmol, 1.9 equiv.) was added dropwise at −78 °C followed immediately with a solution of **SI-8** (5.5 g, 27 mmol, 1.0 equiv.) in THF (27 mL, 1.0 M) dropwise. The reaction mixture was kept at −78 °C for 3 h followed by addition of 3 M NaOAc (40 mL) and H_2O_2 (30% w/w, 35 mL). The resulting mixture was warmed to room temperature and stirred for 14 h. The mixture was then diluted with ethyl acetate (100 mL) and washed with water (60 mL), brine (60 mL) then dried over $MgSO_4$ and the organics concentrated. The crude product was purified by flash column chromatography (PE:EtOAc, 98:2) to yield **SI-9** (5.7 g, 82%) as a colourless oil.

R_f (PE:EtOAc, 95:5)= 0.39

^{1}H NMR (400 MHz, $CDCl_3$): 5.81 (1H, ddd, J = 16.9, 10.8, 7.6, C^2H), 5.06 (ddd, J = 1.8, 1.2, 0.9 Hz, C^1H_{trans}), 5.01 (1H, d, J = 1.2 Hz, C^1H_{cis}), 4.05 (1H, dqd, J = 3.7, 6.0, 9.5 Hz, C^6H), 3.63 (1H, ddd, J = 9.7, 5.7, 1.7 Hz, C^4H), 2.23 (1H, m, C^3H), 1.61 (1H, ddd, J = 14.4, 3.9, 1.8 Hz, C^5H), 1.49 (1H, dt, J = 14.4, 9.7 Hz, C^5H), 1.18 (3H, d, J = 6.1 Hz, C^7H_3), 1.03 (3H, d, J= 6.9 Hz, C^8H_3), 0.90 (9H, s, $SiC(CH_3)_3$), 0.12 (3H, s, $SiCH_3$), 0.10 (3H, s, $SiCH_3$)

^{13}C NMR (100 MHz, $CDCl_3$): 141.2 (C^2), 114.9 (C^1), 74.9 (C^4), 70.6 (C^6), 43.9 (C^3), 42.7 (C^5), 26.0 ($SiCMe_3$), 24.8 (C^7), 18.0 (3 × TBS CH_3), 15.1 (C^8), −3.7 ($SiCH_3$), −4.6 ($SiCH_3$)

$[\alpha]_D^{20.0}$ = −18 (c = 0.91, $CHCl_3$)

Data in accordance with: G. Wang, Y. Ning, E.–i. Negishi, *Chem. Eur. J,* **2011,** *17,* 4118

(5R,7R)-5-((R)-But-3-en-2-yl)-2,2,3,3,7,9,9,10,10-nonamethyl-4,8-dioxa-3,9-disilaundecane, 8

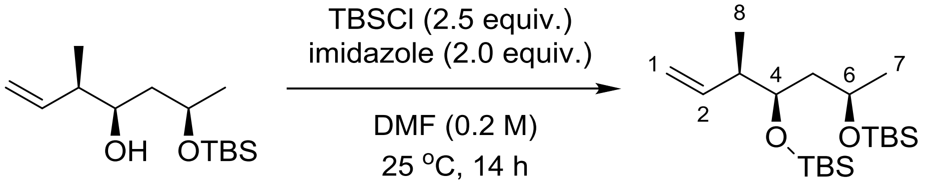

SI-9 **8**

To a stirring solution of alcohol **SI-9** (4.8 g, 19 mmol), imidazole (2.9 g, 38 mmol, 2.0 equiv.) and a catalytic amount of 4-dimethylaminopyridine (1.2 mg, 0.010 mmol) in DMF (95 mL, 0.2 M) was added *tert*-butyldimethylsilyl chloride (7.0 g, 46 mmol, 2.0 equiv.) and the resultant mixture was stirred at room temperature for 14 hr. The reaction mixture was hydrolysed with LiCl solution (5% *w/v*, 150 mL) and extracted with Pentane:CH_2Cl_2 (90:10, 3 × 75 mL). The combined organics were washed with water (80 mL), brine (80 mL), dried over $MgSO_4$ and concentrated. The crude product was purified with flash column chromatography (PE:Et_2O, 99:1) to yield **8** (7.0 g, 99%) as a colourless oil.

R_f (PE:EtOAc, 95:5)= 0.76

^{1}H NMR (400 MHz, $CDCl_3$): 5.91 (1H, ddd, J = 17.3, 10.8, 6.7 Hz, C^2H), 5.02 (1H, ddd, J = 17.3, 1.7, 1.2 Hz, C^1H_{trans}), 4.97 (1H, ddd, J = 10.8, 1.9, 1.4 Hz, C^1H_{cis}), 3.90 (1H, app sx, J = 6.2 Hz, C^6H), 3.68 (1H, ddd, J = 7.1, 5.6, 3.7 Hz, C^4H), 2.33 (1H, m, C^3H), 1.60 (1H, m, C^5H), 1.48 (1H, m C^5H), 1.13 (3H, d, J = 6.1 Hz, C^7H_3) 0.95 (3H, d, J = 6.9 Hz, C^8H_3), 0.89 (9H, s, $SiC(CH_3)_3$), 0.89 (9H, s, $SiC(CH_3)_3$), 0.05 (6H, 2 × $SiCH_3$), 0.04 (9H, 2 × $SiCH_3$)

^{13}C NMR (100 MHz, $CDCl_3$): 141.6 (C^2), 114.0 (C^1), 73.1 (C^4), 66.1 (C^6), 44.0 (C^3), 42.4 (C^5), 26.09 ($SiCMe_3$), 26.05 ($SiCMe_3$), 23.9 (C^7), 18.28 (3 × TBS CH_3), 18.25 (3 × TBS CH_3), 13.9 (C^8), −4.1 ($SiCH_3$), −4.15 ($SiCH_3$), −4.16 ($SiCH_3$), −4.5 ($SiCH_3$)

$[\alpha]_D^{20.0}$ = −17 (c = 0.91, $CHCl_3$)

Data in accordance with: G. Wang, Y. Ning, E.–i. Negishi, *Chem. Eur. J,* **2011,** *17,* 4118

(*5R,7R*)-2,2,3,3,5,9,9,10,10-Nonamethyl-7-((*R,Z*)-4-(4,4,5,5-tetramethyl-1,3,2-dioxaborolan-2-yl)pent-3-en-2-yl)-4,8-dioxa-3,9-disilaundecane, 20

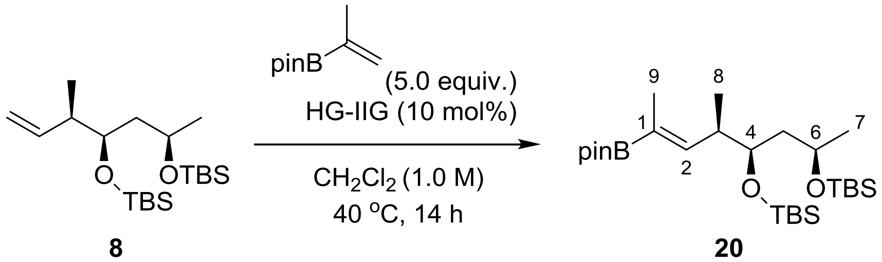

Dry CH_2Cl_2 was obtained through activated alumina purification columns and degassed by freeze pump thaw (4 cycles) and used immediately. Commercial isopropenyl boronic acid pinacol ester contains a phenothiazine stabiliser and was therefore twice distilled under reduced pressure (47 °C, 11 mbar) followed by freeze pump thaw (3 cycles) and used immediately.

A dry round bottomed flask containing alkene **8** (2.0 g, 5.3 mmol, 1 equiv.) was fitted with a reflux condenser and evacuated. Under nitrogen, isopropenyl boronic acid pinacol ester (4.4 g, 26 mmol, 5.0 equiv.) was added followed by CH_2Cl_2 (2 mL, 2.5 M). A solution of fresh Hoveyda-Grubbs 2nd generation catalyst (330 mg, 0.53 mmol, 10 mol%) in CH_2Cl_2 (3 mL) was prepared and 5 mol% added. The solution was heated to 40 °C under nitrogen for 2 h and the reaction mixture was then frozen with liquid nitrogen, exposed to vacuum for 0.5 h and warmed to room temperature and nitrogen reintroduced to remove solvated ethylene. The remaining catalyst solution was added portionwise after every freeze-pump-thaw cycle. The reaction mixture was then heated to 40 °C for 2 h, and this process was repeated 3 more times until no starting material remained. The reaction mixture was diluted with a mixture of hexanes:Et_2O (10:1, 30 mL) and run through a silica pad and the filtrate concentrated and excess isopropenyl boronic acid pinacol ester was removed under vacuum (<1 mbar). The crude product was purified by flash column chromatography (SiO_2 6 × 16 cm, hexanes:Et_2O 98.5:1.5 to 98:2) to yield vinyl boronate **20** (1.6 g, 60%, >99:1 Z/E, 99:1 *dr*) as a pale yellow oil.

R_f (PE:Et_2O 98:2)= 0.4

^{1}H NMR (500 MHz, $CDCl_3$): 6.28 (1H, dq, J = 9.7, 1.7 Hz, C^2H), 3.90 (1H, app sx, J = 6.2 Hz, C^6H), 3.65 (1H, app q, J = 5.9 Hz, C^4H), 2.66 (1H, ddq, J = 9.7, 6.8, 4.9 Hz, C^3H), 1.69 (3H, d, J = 1.7 Hz, C^9H_3), 1.68 (1H, ddd, J = 13.8, 12.4, 6.3 Hz, C^5H), 1.57 (1H, ddd, J = 13.8, 12.8, 6.4 Hz, C^5H), 1.26 (6H, s, 2 × pin CH_3), 1.24 (6H, s, 2 × pin CH_3), 1.14 (3H, d, J = 6.1 Hz, C^7H_3), 0.93 (3H, d, J = 6.8 Hz, C^8H_3), 0.90 (9H, s, $SiC(CH_3)_3$), 0.88 (9H, s, $SiC(CH_3)_3$), 0.05 (2 × 3H, 2 × s, 2 × $SiCH_3$), 0.04 (6H, s, 2 × $SiCH_3$)

<u>^{13}C NMR</u> (125 MHz, CDCl$_3$): 149.9 (C^2), 83.1 (2 × pin C(Me)$_2$), 72.8 (C^4), 66.2 (C^6), 45.7 (C^5), 38.2 (C^3), 26.2 (SiCMe$_3$), 26.1 (SiCMe$_3$), 25.2 (2 × pin CH$_3$), 24.8 (2 × pin CH$_3$), 24.3(C^8), 18.30 (3 × TBS CH$_3$), 18.21 (3 × TBS CH$_3$), 14.8 (C^7), 14.3 (C^9), −3.9 (SiCH$_3$), −4.2 (SiCH$_3$), −4.3 (SiCH$_3$), −4.5 (SiCH$_3$)

<u>^{11}B NMR</u> (96 MHz, CDCl$_3$): 29.0

<u>IR</u> ν_{max}(neat)/cm^{-1}= 2929, 1634, 1370, 1141, 833, 722

$[\alpha]_D^{20.0}$ = +7.9 (c = 0.89, CHCl$_3$)

<u>HMRS</u> (ESI$^+$): calcd. for $C_{27}H_{57}BO_4Si_2Na$ (M+Na): 535.3786, found 535.3782

(*5R,7R*)-2,2,3,3,5,9,9,10,10-Nonamethyl-7-((*R,E*)-4-(4,4,5,5-tetramethyl-1,3,2-dioxaborolan-2-yl)but-3-en-2-yl)-4,8-dioxa-3,9-disilaundecane, 21

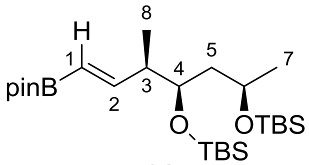

21

1,1-disubstituted boronic ester **21** eluted after trisubstituted boronic ester **20** and was isolated (<5%) as a pale yellow oil.

<u>R$_f$</u> (PE:Et$_2$O, 98:2)= 0.33

<u>^{1}H NMR</u> (400 MHz, CDCl$_3$): 6.66 (1H, dd, *J* = 18.1, 6.9 Hz, C^{2}H), 5.42 (1H, dd, *J* = 18.1, 1.3 Hz C^{1}H), 3.88 (1H, dqd, *J* = 6.2 Hz, C^{6}H), 3.74 (1H, td, *J* = 6.4, 3.9 Hz, C^{4}H), 2.37 (1H, dddq, *J* = 6.9, 6.9, 4.0, 1.5 Hz, C^{3}H), 1.62 (1H, ddd, *J* = 13.7, 6.4, 6.4 Hz, C^{5}H), 1.56 (1H, ddd, *J* = 13.7, 6.5, 6.4 Hz, C^{5}H), 1.26 (6H, s, 2 × pin CH$_3$), 1.25 (6H, s, 2 × pin CH$_3$), 1.12 (3H, d, *J* = 6.0 Hz, C^{7}H$_3$), 0.96 (3H, d, *J* = 6.8 Hz, C^{8}H$_3$), 0.88 (9H, s, SiC(CH$_3$)$_3$),0.87 (9H, s, SiC(CH$_3$)$_3$), 0.04 (6H, s, 2 × SiCH$_3$), 0.02 (3H, s, SiCH$_3$), 0.02 (3H, s, SiCH$_3$)

<u>^{13}C NMR</u> (100 MHz, CDCl$_3$): 157.7 (C^2), 83.0 (2 × pin C(Me)$_2$), 72.7 (C^4), 65.9 (C^6), 44.7 (C^5), 44.1 (C^3), 26.1 (SiCMe$_3$), 26.0 (SiCMe$_3$), 24.9 (2 × pin CH$_3$), 24.8 (2 × pin CH$_3$), 24.0 (C^8), 18.20 (3 × TBS CH$_3$), 18.15 (3 × TBS CH$_3$), 13.2 (C^7), −4.19 (SiCH$_3$), −4.21 (SiCH$_3$), −4.22 (SiCH$_3$), −4.5 (SiCH$_3$)

<u>^{11}B NMR</u> (96 MHz, CDCl$_3$): 29.1

<u>IR</u> ν_{max} (neat)/cm^{-1}= 2957, 1637, 1360, 1145, 833

$[\alpha]_D^{20.1}$ = +13 (c = 0.98, CHCl$_3$)

<u>HMRS</u>: (ESI$^+$) calcd. for $C_{26}H_{55}BO_4Si_2Na$ (M+Na): 521.3629, found 521.3620

(*5R,7R*)-2,2,3,3,5,9,9,10,10-Nonamethyl-7-((*R,E*)-4-methyl-5-(4,4,5,5-tetramethyl-1,3,2-dioxaborolan-2-yl)pent-3-en-2-yl)-4,8-dioxa-3,9-disilaundecane, 7

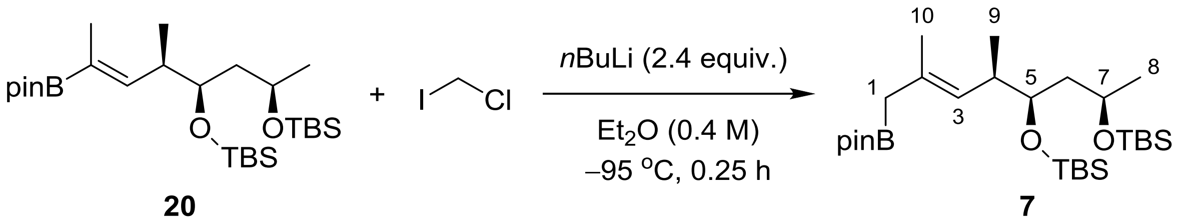

Following **GP1** on a 2.4 mmol scale with vinyl boronate **20** to yield **7** (1.3 g, 99%) as a colourless oil.

<u>R_f</u> (Hexanes:Et_2O, 99:1)= 0.26

<u>^{1}H NMR</u> (500MHz, $CDCl_3$): 5.02 (1H, d, J = 9.5 Hz, C^3H), 3.90 (1H, app sx, J = 6.2 Hz, C^7H), 3.55 (1H, app q, J = 6.0 Hz, C^5H), 2.42 (1H, ddq, J = 9.6, 6.8, 4.7 Hz, C^4H), 1.65 (3H, d, J = 1.1 Hz, $C^{10}H_3$), 1.63 (2H, s, C^1H_2), 1.60 – 1.56 (2H, 2 × m, C^6H_2), 1.23 (12H, s, 4 × pin CH_3), 1.12 (3H, d, J = 6.0 Hz, C^8H_3), 0.89 – 0.87 (21H, 2 × $SiC(CH_3)_3$, 1 × C^9H_3), 0.04 (6H, s, 2 × $SiCH_3$), 0.03 (6H, s, 2 × $SiCH_3$)

<u>^{13}C NMR</u> (125 MHz, $CDCl_3$): 130.7 (C^2), 128.3 (C^3), 83.1 (2 × pin $C(Me)_2$), 73.6 (C^5), 66.3 (C^7), 45.7 (C^6), 38.3 (C^4), 26.2 ($SiCMe_3$), 26.1 ($SiCMe_3$), 24.93 (2 × pin CH_3), 24.90 (2 × pin CH_3), 24.2 (C^8), 18.517 (C^{10}), 18.31 (3 × TBS CH_3), 18.21 (3 × TBS CH_3), 16.1 (C^9), −3.9 ($SiCH_3$), −4.1 ($SiCH_3$), −4.2 ($SiCH_3$), −4.4 ($SiCH_3$)

<u>^{11}B NMR</u> (96 MHz, $CDCl_3$): 32.4

<u>IR v_{max}</u> (neat)/cm^{-1}: 2928, 1472, 1321, 1142, 1044, 833

$[\alpha]_D^{20.0}$ = +1.8 (c = 0.92, $CHCl_3$)

<u>HMRS</u> (ESI$^+$): calcd. for $C_{28}H_{59}BO_4Si_2Na$ (M+Na): 549.3943, found 549.3940

(*5R,7R*)-2,2,3,3,5,9,9,10,10-Nonamethyl-7-((*2R,6S,E*)-4-methyl-6-(4,4,5,5-tetramethyl-1,3,2-dioxaborolan-2-yl)hept-3-en-2-yl)-4,8-dioxa-3,9-disilaundecane, 4

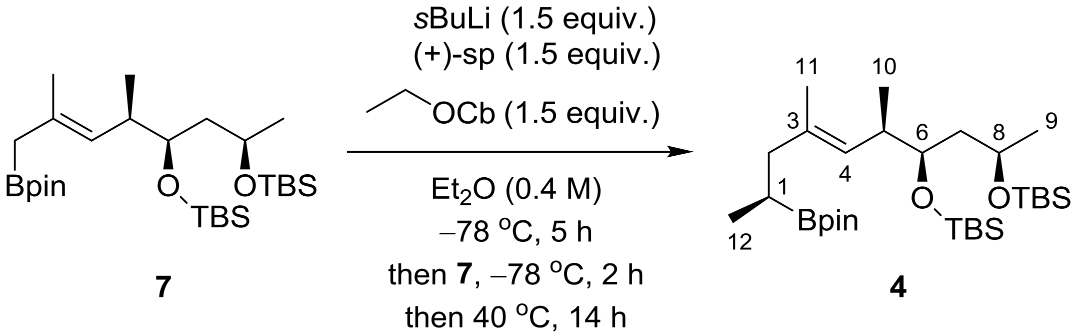

Following **GP2** on a 2.4 mmol scale with allylic boronate **7** and 12 h reflux. The crude oil was purified by flash column chromatography (SiO_2, 4 × 15 cm PE:Et_2O, 98:2) to yield secondary boronic ester **4** (1.1 g, 81%) as a colourless oil.

<u>R_f</u> (PE:Et_2O, 99:1)= 0.23

<u>^{1}H NMR</u> (500 MHz, $CDCl_3$): 5.07 (1H, d, *J* = 9.3 Hz, C^4H), 3.89 (1H, sx, *J* = 6.3 Hz, C^8H), 3.56 (1H, app q, *J* = 4.9 Hz, C^6H), 2.45 (1H, ddq, *J* = 9.7, 6.8, 4.9 Hz, C^5H), 2.19 (1H, dd, *J* = 13.9, 5.5 Hz, C^7H), 1.83 (1H, dd, *J* = 13.9, 9.9 Hz, C^7H), 1.64 (1H, m, C^2H), 1.56 – 1.54 (1H, m, C^2H), 1.56 (3H, $C^{11}H_3$), 1.23 (12H, s, 4 × pin CH_3), 1.17 (1H, m, C^1H), 1.12 (3H, d, *J* = 6.1 Hz, C^9H_3), 0.88 (12H, m, $SiC(CH_3)_3$ and $C^{10}H_3$), 0.87 (9H, s, $SiC(CH_3)_3$), 0.86 (3H, m, $C^{12}H_3$), 0.04 (3H, s, 1 × $SiCH_3$), 0.03 (9H, s, 3 × $SiCH_3$)

<u>^{13}C NMR</u> (125 MHz, $CDCl_3$):

133.1 (C^3), 129.1 (C^4), 83.0 (2 × pin $C(Me)_2$), 73.5 (C^6), 66.2 (C^8), 45.3 (C^2), 42.3 (C^7), 37.9 (C^5), 26.2 ($SiCMe_3$), 26.1 ($SiCMe_3$), 24.90 (2 × pin CH_3), 24.88 (2 × pin CH_3), 24.1 (C^9), 18.28 (3 × TBS CH_3), 18.26 (3 × TBS CH_3), 16.21 (C^{10}), 16.18 (C^{11}), 14.4 (C^{12}), −3.9 ($SiCH_3$), −4.17 ($SiCH_3$), −4.20 ($SiCH_3$), −4.5 ($SiCH_3$).

<u>^{11}B NMR</u> (96 MHz, $CDCl_3$): 35.5

<u>IR ν_{max}</u> (neat)/cm^{-1}: 2929, 1461, 1253, 1144, 833, 772

$[\alpha]_D^{20.0}$ = −2.9 (c = 1.0, $CHCl_3$)

<u>HMRS</u> (ESI$^+$): calcd. for $C_{30}H_{63}BO_4Si_2Na$ (M+Na): 577.4256, found 577.4283

(*2S,6R,7R,9R,E*)-7,9-bis((*tert*-Butyldimethylsilyl)oxy)-4,6-dimethyldec-4-en-2-ol, SI-10

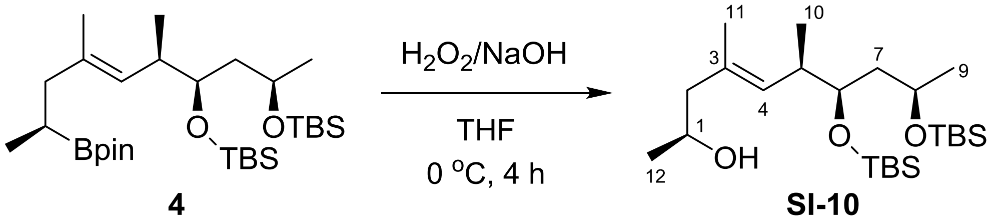

Following **GP3** on a 0.12 mmol scale with secondary boronic ester **4** and the crude oil was purified by flash column chromatography (2 × 15 cm SiO_2, PE:Et_2O, 80:20) to yield secondary alcohol **SI-10** (54 mg, 99%, 97:3 *dr*) as a colourless oil.

<u>R$_f$</u> (PE:Et_2O, 80:20)= 0.28

<u>^{1}H NMR</u> (500 MHz, $CDCl_3$): 5.25 (1H, d, *J* = 9.6 Hz, C^4H), 3.89 (1H, dqd, *J* = 6.2 Hz, C^8H), 3.85 (1H, dqd, *J* = 6.2 Hz, C^1H), 3.62 (1H, app q, *J* = 4.9 Hz, C^6H), 2.53 (1H, dddq, *J* = 6.9, 6.9, 4.0, 1.5 Hz, C^5H), 2.14 (1H, dd, *J* = 13.2, 4.0 Hz, C^7H), 2.03 (1H, dd, *J* = 13.2, 8.9 Hz, C^7H), 1.64 (3H, d, *J* = 1.3 Hz, $C^{11}H_3$), 1.62 (1H, ddd, *J* = 13.5, 6.5, 6.5 Hz, C^2H), 1.56 (1H, ddd, *J* = 13.7, 6.5, 6.4 Hz, C^2H), 1.18 (3H, d, *J* = 6.2 Hz, $C^{12}H_3$), 1.13 (3H, d, *J* = 6.1 Hz, C^9H_3), 0.88 (3H, d, *J* = 6.9 Hz, $C^{10}H_3$), 0.88 (9H, s, 3 × TBS CH_3), 0.87 (9H, s, 3 × TBS CH_3), 0.05 (3H, s, $SiCH_3$), 0.04 (6H, s, 2 × $SiCH_3$), 0.03 (3H, s, $SiCH_3$)

<u>^{13}C NMR</u> (125 MHz, $CDCl_3$): 131.7 (C^3), 131.4 (C^4), 73.1 (C^8), 66.1 (C^1), 64.9 (C^6), 50.3 (C^2), 44.4 (C^7), 37.7 (C^5), 26.1 ($SiCMe_3$), 26.0 ($SiCMe_3$), 24.0 (C^9), 22.8 (C^{12}), 18.3 (3 × TBS CH_3), 18.2 (3 × TBS CH_3), 16.7 (C^{11}), 15.6 (C^{10}), −4.0 ($SiCH_3$), −4.17 ($SiCH_3$), −4.19 ($SiCH_3$), −4.5 ($SiCH_3$)

<u>IR v_{max}</u> (neat)/cm^{-1}: 3352, 2928, 1251, 1083, 833

$[\alpha]_D^{20.8}$ = +11 (c = 1.2, $CHCl_3$)

<u>HMRS</u> (ESI$^+$): calcd. for $C_{24}H_{52}O_3Si_2Na$ (M+Na): 467.3353, found 467.3347

(*5R,7R*)-2,2,3,3,5,9,9,10,10-Nonamethyl-7-((*2R,6S,E*)-4-methyl-6-(4,4,5,5-tetramethyl-1,3,2-dioxaborolan-2-yl)hept-3-en-2-yl)-4,8-dioxa-3,9-disilaundecane, 3

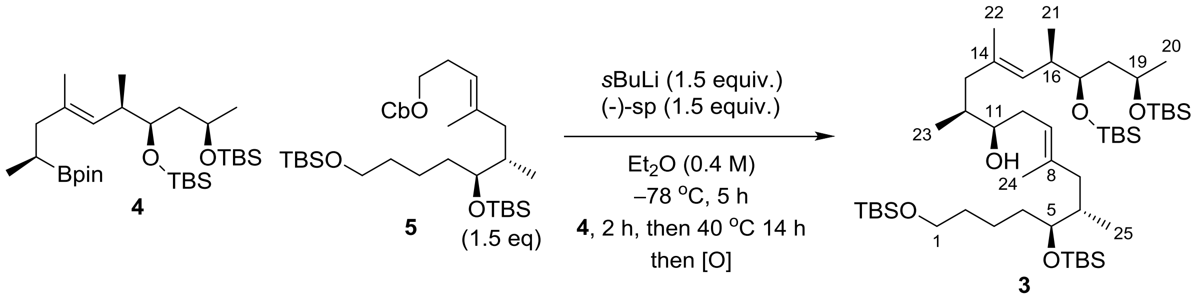

Following **GP2** on a 1.3 mmol scale with secondary boronic ester **4.** After 10 h reflux the reaction mixture concentrated. **GP3** was then followed in THF (8 mL) and 30% H_2O_2 : 3 M NaOH (8 mL, 1:1 *v/v)* and the resultant crude oil was purified by flash column chromatography (SiO_2, 4 × 17 cm, PE:EtOAc, 98.5:1.5 to 98:2) to yield secondary alcohol **3** (950 mg, 82%) as a colourless oil.

R_f (PE:EtOAc, 90:10)= 0.42

^{1}H NMR (500MHz, $CDCl_3$): 5.17 (1H, t, *J* = 7.2 Hz, C^9H), 5.12 (1H, t, *J* = 9.5 Hz, $C^{15}H$), 3.90 (1H, app sx, *J* = 6.2 Hz, $C^{19}H$), 3.60 (2H, t, *J* = 6.4 Hz, C^1H_2), 3.57 (1H, m, $C^{17}H$), 3.52 (1H, m, C^5H), 3.42 (1H, m, *J* = 4.7 Hz, $C^{11}H$), 2.47 (1H, m, $C^{16}H$), 2.29 (1H, dd, *J* = 12.3, 2.3 Hz, $C^{18}H$), 2.21 – 2.14 (3H, m, $C^{18}H$, $C^{10}H_2$), 1.79 − 1.71 (4H, m, C^7H_2, $C^{12}H$), 1.61 (3H, s, $C^{24}H_3$), 1.58 (3H, s, $C^{22}H_3$), 1.53 – 1.48 (2H, m, C^3H_2), 1.43 – 1.35 (3H, m, C^2H_2, C^4H), 1.30 – 1.23 (2H, m, C^4H), 1.13 (3H, d, *J* = 6.0 Hz, $C^{20}H_3$), 0.89 – 0.86 (6H, m, $C^{21}H_3$, 3H), 0.89 (27H, 3 × $SiC(CH_3)_3$), 0.88 (9H, $SiC(CH_3)_3$), 0.82 (3H, d, *J* = 6.5 Hz, $C^{23}H_3$), 0.74 (3H, d, *J* = 6.4 Hz, $C^{25}H_3$), 0.05 (12H, s, 4 × $SiCH_3$), 0.04 (12H, s, 4 × $SiCH_3$)

^{13}C NMR (125 MHz, $CDCl_3$): 138.1 (C^8), 132.4 (C^{14}), 130.4 (C^{15}), 122.0 (C^9), 76.1 (C^5), 75.9 (C^{11}), 73.3 (C^{17}), 66.2 (C^{19}), 63.3 (C^1), 45.3 (C^{13}), 43.1 (C^{18}), 42.9 (C^7), 37.7 (C^{16}), 36.2 (C^{12}), 35.7 (C^6), 33.34 (C^2), 33.29 (C^3), 32.7 (C^{10}), 26.14 (3 × $SiMe_3$), 26.07 ($SiCMe_3$), 24.2 (C^{20}), 22.5 (C^4), 18.5 (3 × TBS CH_3), 18.33 (3 × TBS CH_3), 18.29 (3 × TBS CH_3), 18.26 (3 × TBS CH_3), 16.34 (C^{22}), 16.31 (C^{24}), 15.8 (C^{21}), 15.1 (C^{23}), 14.1 (C^{25}), −3.91 ($SiCH_3$), −4.0 ($SiCH_3$), −4.14 ($SiCH_3$), −4.15 ($SiCH_3$), −4.22 ($SiCH_3$), −4.5 ($SiCH_3$), −5.11 (2 × $SiCH_3$)

$[\alpha]_D^{21.1}$ = −0.90 (c = 1.1, $CHCl_3$)

Data in accordance with: A. B. Benowitz, S. Fidanze, P. L. C. Small, Y. Kishi, *J. Am. Chem. Soc.* **2001,** *123,* 5128 – 5129; **and** G. Wang, Y. Ning, E.–i. Negishi, *Chem. Eur. J,* **2011**, *17,* 4118

(*S*)-2,2,3,3,11,11,12,12-Octamethyl-5-((*S,E*)-4-methylhepta-4,6-dien-2-yl)-4,10-dioxa-3,11-disilatridecane, 22

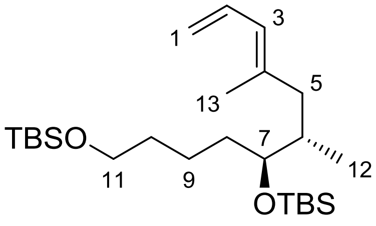

22

Diene **22** was purified via flash column chromatography from the crude of **3** (SiO_2, Hexanes:Et_2O 99.5:0.5) to yield **22** (60 mg, 11%) as a yellow oil.

R_f (Hexanes:Et_2O, 99:1)= 0.38

^{1}H NMR (500 MHz, $CDCl_3$): 6.59 (1H, dt, *J* = 16.8, 10.6 Hz, C^3H), 5.84 (1H, d, *J* = 10.8 Hz, C^2H), 5.09 (1H, dd, *J* = 16.8, 1.6 Hz, C^1H^{trans}) 4.97 (1H, dd, *J* = 10.2, 1.5 Hz, C^1H^{cis}), 3.62 (2H, t, *J* = 6.4 Hz, $C^{11}H$), 3.53 (1H, m, C^7H), 2.26 (1H, m), 1.79 (1H, m), 1.73 (3H, s), 1.54 – 1.48 (2H, m), 1.43 – 1.37 (2H, m), 1.31 – 1.24 (2H, m), 1.14 (1H, m), 0.91 (9H, s, 3 × TBS CH_3), 0.90 (9H, s, 3 × TBS CH_3), 0.76 (3H, *J* = 6.4 Hz, $C^{12}H_3$), 0.06 (6H, s, $SiCH_3$), 0.05 (6H, s, $SiCH_3$)

^{13}C NMR (125 MHz, $CDCl_3$): 138.6 (C^4), 133.4 (C^3), 126.9 (C^2), 114.4 (C^1), 75.8 (C^7), 63.2 (C^{11}), 42.9 (C^5), 35.8 (C^6), 33.2 (C^{10}), 33.1 (C^9), 26.0 (2 × $SiMe_3$), 22.4 (C^8), 18.4 (3 × TBS CH_3), 18.2 (3 × TBS CH_3), 16.5 (C^{12}), 13.9 (C^{13}), −4.1 ($SiCH_3$), −4.4 ($SiCH_3$), −5.3 (2 × $SiCH_3$)

IR ν_{max} (neat)/cm^{-1}: 2929, 1472, 1255, 1098, 835, 773

$[\alpha]_D^{20.9}$ = −4.2 (c = 0.47, $CHCl_3$)

HMRS (ESI$^+$): calcd for $C_{25}H_{52}O_2Si_2Na$ (M+Na) = 463.3403, found 463.3413

Process from 20 to 3 without chromatographic purification

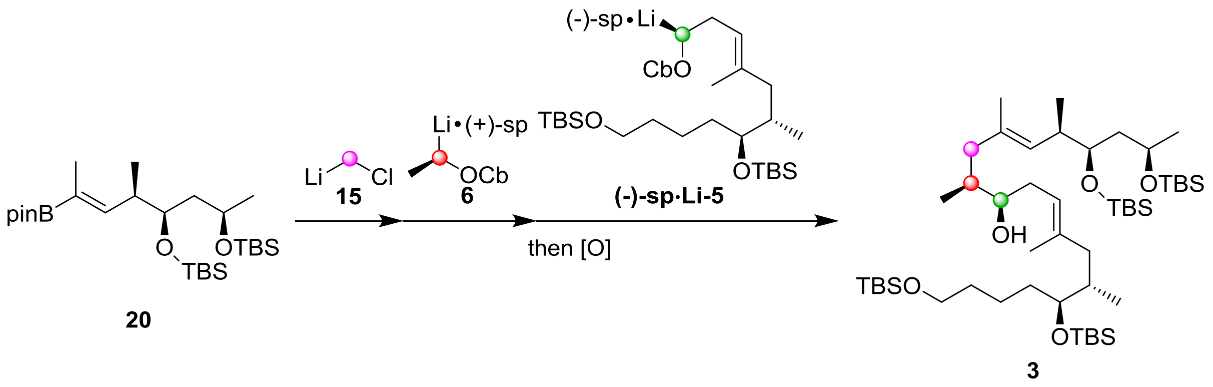

Following **GP1** with 1.0 mmol (510 mg) of vinyl boronic ester **20** yielded allylic boronic ester **7** (524 mg, 99%) as a colourless oil. This was used directly in the next step.

A solution of **7** (520 mg, 1.0 mmol, 1.0 equiv.) in Et_2O (2 mL) was added into a solution of lithiated carbamate **6** (1.5 mmol, 1.5 equiv., synthesised following **GP2**) dropwise and stirred for 2 h at −78 °C. The reaction mixture was slowly warmed to room temperature and subsequently heated to 40 °C for 12 h. The resultant solution was taken up in a syringe.

The crude solution was added into a solution of lithiated carbamate **(-)-sp·Li-5** (1.5 mmol, 1.5 equiv., synthesised following **GP2**) dropwise and stirred for 2 h at −78 °C. The reaction mixture was slowly warmed to room temperature and subsequently heated to 40 °C for 10 h. The reaction mixture was concentrated *in vacuo.*

The crude reaction mixture was taken up in THF (4 mL), cooled to 0 °C and oxidised following **GP3** with 3 M NaOH:30% H_2O_2 (1:1 *v/v*, 4 mL).

The crude alcohol was purified via flash column chromatography (SiO_2, 4 ×17 cm, PE:EtOAc, 99:1 to 98:2) to yield secondary alcohol **3** (750 mg, 86%) as a colourless oil.

7 Endgame

(*5S,6S,8E,11R,12S,14E,16R,17R,19R*)-5,17,19-Tris((*tert*-butyldimethylsilyl)oxy)-6,8,12,14,16-pentamethylicosa-8,14-diene-1,11-diol, SI-11

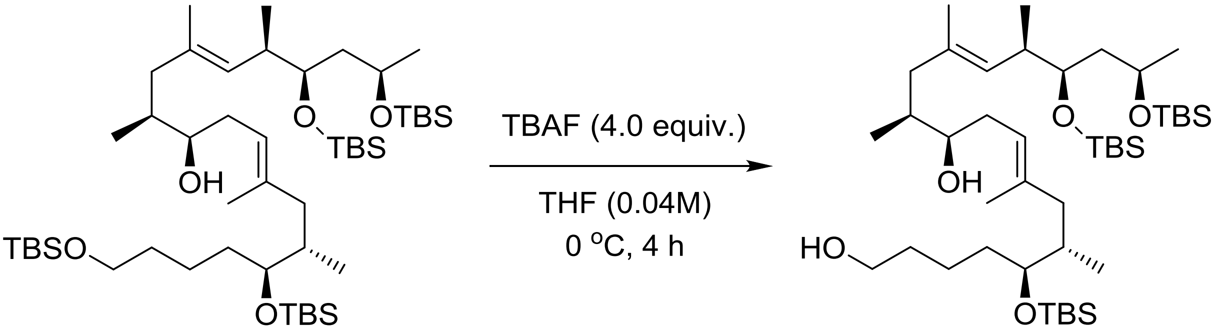

3 **SI-11**

A solution of Silyl ether **3** (620 mg, 0.70 mmol) in dry THF (18 mL, 0.04 M) was cooled to 0 °C and with stirring a freshly prepared solution of TBAF·3H_2O in THF (0.5 M, 5.6 mL, 2.8 mmol, 4.0 equiv.) was added dropwise and stirred. Reaction was monitored by TLC (with acidic quench) until all of the starting material was consumed (8 h). The reaction was quenched with saturated NH_4Cl (20 mL) and extracted with EtOAc (3 × 20 mL). The combined organics were washed with brine, dried over $MgSO_4$ and concentrated. The crude material was purified by flash chromatography (SiO_2, 3 × 16 cm, Hexanes:EtOAc, 88:22) to yield alcohol **SI-11** (460 mg, 85%) as a colourless oil.

R_f (PE:EtOAc, 80:20)= 0.28

^{1}H NMR (500 MHz, $CDCl_3$): 5.17 (1H, t, *J* = 7.3 Hz), 5.12 (1H, d, *J* = 9.4 Hz), 3.89 (1H, sept, *J* = 6.2 Hz), 3.63 (2H, t, *J* = 6.6 Hz), 3.59 (1H, td, *J* = 6.1, 4.5 Hz), 3.53 (1H, m), 3.42 (1H, m), 2.47 (1H, m), 2.28 (1H, dd, *J* = 12.5, 2.5 Hz), 2.23 – 2.15 (3H, m), 1.81 – 1.66 (4H, m), 1.60 (3H, m), 1.58 (3H, m), 1.64 – 1.52 (6H, m), 1.45 – 1.36 (3H, m), 1.33 – 1.24 (2H, m), 1.13 (3H, d, *J* = 6.0 Hz), 0.89 – 0.87 (20H, m), 0.81 (3H, d, *J* = 6.4 Hz), 0.74 (3H, d, *J* = 6.4 Hz), 0.04 (6H, s), 0.03 (12H, 2 × s)

^{13}C NMR (125 MHz, $CDCl_3$): 138.0, 132.4, 130.4, 122.1, 75.94, 75.88, 73.3, 66.2, 63.1, 45.3, 43.1, 42.9, 37.7, 36.2, 35.7, 33.3, 33.2, 32.7, 26.2, 26.12, 26.06, 24.2, 22.3, 18.33, 18.29, 18.26, 16.4, 16.3, 15.8, 15.1, 14.1, −3.9, −4.0, −4.1, −4.2, −4.5

$[\alpha]_D^{20.9}$ = −3.7 (c = 0.27, $CHCl_3$)

Data in accordance with: A. B. Benowitz, S. Fidanze, P. L. C. Small, Y. Kishi, *J. Am. Chem. Soc.* **2001,** *123,* 5128 – 5129; **and** G. Wang, Y. Ning, E. –i. Negishi, *Chem. Eur. J,* **2011**, *17,* 4118

(*5S,6S,8E,11R,12S,14E,16R,17R,19R*)-5,17,19-Tris((*tert*-butyldimethylsilyl)oxy)-11-hydroxy-6,8,12,14,16-pentamethylicosa-8,14-dienoic acid, 23

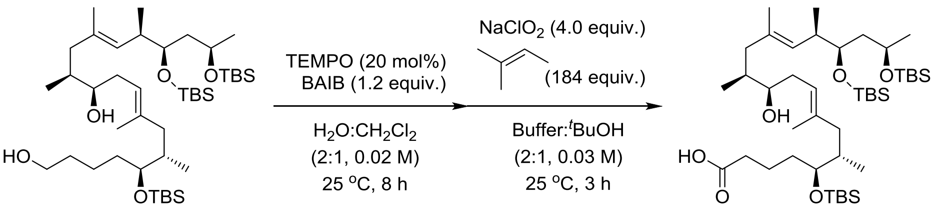

SI-11 **23**

Alcohol **SI-11** (440 mg, 0.57 mmol, 1 equiv.) was dissolved in a mixture of H_2O/CH_2Cl_2 (29.5 mL, 2:1 *v/v*, 0.02 M) and stirred vigorously at room temperature. TEMPO (18 mg, 0.11 mmol, 20 mol%) and bisacetoxyiodobenzene (220 mg, 0.68 mmol, 1.2 equiv.) were added in one portion and stirred at 25 °C for 4.5 h. The reaction was quenched with saturated $NaHCO_3$ (30 mL) and the organics separated. The aqueous layer was further extracted with CH_2Cl_2 (2 × 25 mL) and the organics were combined, washed with brine (20 mL), dried with $MgSO_4$ and concentrated. The crude oil was taken up in Et_2O and the slurry run through a short silica pad and concentrated.

The crude aldehyde was dissolved in a mixture of aqueous NaH_2PO_4 buffer (0.67 M)/^{t}BuOH (16 mL, 2:1 *v/v*, 0.03 M) and stirred at 25 °C. 2-Methyl-2-butene (5.3 mL, 105 mmol, 184 equiv.) was added followed by $NaClO_2$ (210 mg, 2.28 mmol, 4.0 equiv.) and the reaction was monitored by TLC (stained with PMA). After 2 h the reaction mixture was quenched with NH_4Cl (30 mL), and extracted with EtOAc (3 × 25 mL). The combined organics were washed with brine, dried with $MgSO_4$ and concentrated. The crude oil was purified by flash column chromatography (SiO_2, 4 × 16 cm, CH_2Cl_2:MeOH 99:1 to 98:2) to yield acid **23** (362 mg, 81%) as a yellow oil.

R_f (CH_2Cl_2:MeOH, 98:2)= 0.32

^{1}H NMR ($CDCl_3$, 500 MHz): 5.17 (1H, t, *J* = 7.3 Hz,), 5.12 (1H, d, *J* = 9.4 Hz), 3.60 (1H, app sx, *J* = 6.2 Hz), 3.54 (1H, m), 3.43 (1H, m), 3.42 (1H, m), 2.47 (1H, dddq, *J* = 6.8, 6.8, 4.4, 1.5 Hz), 2.34 (2H, t, *J* = 7.3 Hz), 2.28 (1H, dd, *J* = 2.7, 12.8 Hz), 2.22 – 2.13 (4H, m), 1.78 – 1.70 (6H, m), 1.62 – 1.57 (2H, m), 1.60 (3H, s), 1.57 (3H, s), 1.53 – 1.38 (4H, m), 1.12 (3H, d, *J* = 6.3 Hz), 0.89 – 0.86 (33H, 3 × 9 H, s, and 6H, m), 0.81 (3H, d, *J* = 6.4 Hz), 0.74 (3H, d, *J* = 6.2 Hz), 0.04 (9H, 3 × 3H, 3 × s), 0.03 (9H, 3 × s)

^{13}C NMR (125 MHz, $CDCl_3$): 178.5, 137.8, 132.4, 130.4, 122.1, 76.0, 75.6, 73.3, 66.2, 45.2, 42.94, 42.88, 37.7, 36.2, 35.7, 34.2, 32.8, 32.7, 26.12, 26.08, 26.01, 24.2, 21.3, 18.27, 18.27, 18.24, 16.34, 16.30, 15.8, 15.0, 14.2, −3.9, −4.1, −4.3, −4.5

$[\alpha]_D^{21.0}$ = −0.86 (c = 1.2, $CHCl_3$)

Data in accordance with: G. Wang, Y. Ning, E. –i. Negishi, *Chem. Eur. J,* **2011**, *17,* 4118

(*6S,7S,12R,E*)-12-((*2S,6R,7R,9R,E*)-7,9-Bis((*tert*-butyldimethylsilyl)oxy)-4,6-dimethyldec-4-en-2-yl)-6-((*tert*-butyldimethylsilyl)oxy)-7,9-dimethyloxacyclododec-9-en-2-one, SI-12

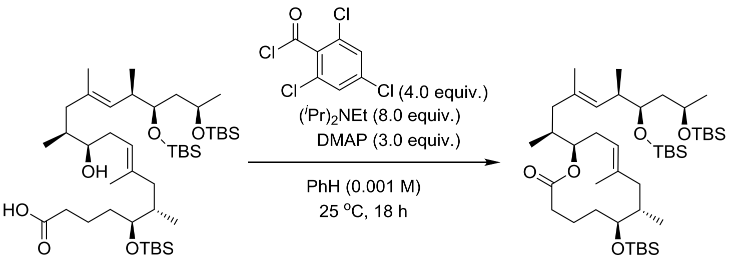

23 **SI-12**

In a dry flask under nitrogen, acid **23** (340 mg, 0.43 mmol, 1.0 equiv.) was dissolved in benzene (9 mL, 0.047 M) and to it was added Hunig's base (600 µL, 3.4 mmol, 8.0 equiv.), followed by 2,4,6-trichlorobenzoyl chloride (270 µL, 1.7 mmol, 4.0 equiv.) and stirred for 1 h at 25 °C. A solution of DMAP (210 mg, 1.7 mmol, 4.0 equiv.) in benzene (190 mL, 0.009 M), was prepared under nitrogen. With vigorous stirring, the solution derived from acid **23** was added to the DMAP solution by syringe pump over 12 h at 25 °C. The resultant cloudy white solution was stirred for a further 6 h. To the reaction mixture was added saturated $NaHCO_3$ (150 mL) and the organics separated. The organics were washed with brine, dried over $MgSO_4$ and concentrated. The crude material was purified by flash column chromatography (SiO_2, 3 × 15 cm, PE:Et_2O, 98:2) to yield lactone **SI-12** (269 mg, 81%) as a colourless oil.

R_f (Hexanes:EtOAc, 98:2)= 0.38

^{1}H NMR ($CDCl_3$, 500 MHz): 5.11 (1H, d, *J* = 9.4 Hz), 5.00 (1H, d, *J* = 10.3 Hz), 4.83 (1H, ddd, *J* = 11.6, 5.8, 3.0 Hz), 3.89 (1H, ddq, *J* = 6.2, 6.2 Hz), 3.58 (1H, ddd, *J* = 5.8, 5.8, 5.8 Hz), 3.36 (1H, dd, *J* = 9.0, 1.8 Hz), 2.48 (2H, tt, *J* = 12.2, 3.2 Hz), 2.41 (1H, dd, *J* = 13.5, 11.6 Hz), 2.12 (1H, dd, *J* = 13.3, 3.9 Hz), 2.03 (1H, d, *J* = 13.8 Hz), 1.96 (1H, td, *J* = 12.3, 3.1 Hz), 1.90 – 1.80 (4H, m), 1.72 – 1.62 (5H, m), 1.67 (3H, s), 1.62 – 1.52 (2H, m), 1.58 (3H, s), 1.13 (3H, d, *J* = 6.0 Hz), 0.96 (3H, d, *J* = 6.8 Hz), 0.89 (9H, s), 0.88 (9H, s), 0.87 (9H + 3H, m), 0.83 (3H, d, *J* = 6.8 Hz), 0.04 – 0.03 (18H, 3 × s)

^{13}C NMR (125 MHz, $CDCl_3$): 173.6, 137.4, 131.8, 130.6, 121.6, 77.6, 75.9, 73.3, 66.2, 45.5, 45.2, 43.3, 37.8, 36.1, 35.3, 33.59, 33.57, 30.3, 26.2, 26.11, 26.08, 24.2, 21.9, 18.8, 18.30, 18.28, 18.25, 16.2, 15.92, 15.87, 14.6, −3.9, −4.0, −4.16, −4.21, −4.5, −4.6

$[\alpha]_D^{21.2}$ = −18.0 (c = 0.78, $CHCl_3$)

Data in accordance with: A. B. Benowitz, S. Fidanze, P. L. C. Small, Y. Kishi, *J. Am. Chem. Soc.* **2001,** *123,* 5128 – 5129; **and** G. Wang, Y. Ning, E. –i. Negishi, *Chem. Eur. J,* **2011**, *17,* 4118

Mycolactone Core, 2

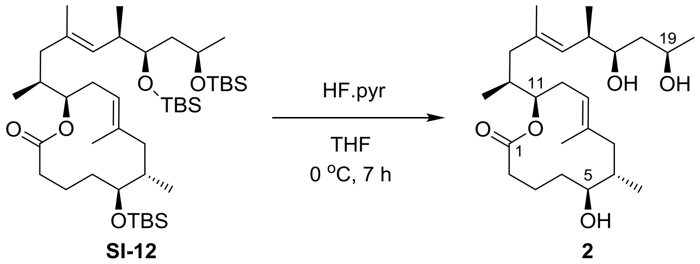

In a PTFE vial, a stirring solution of protected mycolactone core **SI-12** (260 mg, 0.33 mmol) in THF (8 mL, 0.04M) was cooled to 0 °C and to it was added dropwise HF·pyridine (2.6 mL, 30% pyridine). The reaction mixture was then allowed to warm to room temperature and stirred for 7 h (TLC monitored). Once complete, the reaction was slowly quenched with saturated $NaHCO_3$ (25 mL) and the aqueous layer extracted with EtOAc (2 × 25 mL). The organics were combined, washed with brine (30 mL), dried over $MgSO_4$ and concentrated. The crude product was purified by flash column chromatography (SiO_2, 3 × 16 cm, CH_2Cl_2:MeOH 99:1 to 98.25:1.75) to yield mycolactone core **2** (110 mg, 80%) as a colourless oil.

R_f (CH_2Cl_2:MeOH, 95:5) = 0.35

^{1}H NMR (600 MHz, CD_3OD): 5.03 (1H, d, *J* = 11.2 Hz), 5.00 (1H, d, *J* = 10.0 Hz), 4.93 (1H, ddd, *J* = 11.7, 5.0, 2.7 Hz), 3.95 (1H, app sx, *J* = 6.2 Hz), 3.42 (1H, ddd, *J* = 10.1, 7.7, 2.3 Hz), 3.34 (1H, m), 2.49 (1H, ddd, *J* = 14.0, 11.6 Hz), 2.40 (1H, tt, *J* = 13.8, 6.5 Hz), 2.34 (1H, ddd, *J* = 13.1, 7.9, 2.5 Hz), 2.15 (1H, m), 2.11 (1H, dd, *J* = 13.5, 6.1 Hz), 2.07 (1H, br. d, *J* = 14.0 Hz), 1.97 – 1.93 (2H, m), 1.90 (1H, dd, *J* = 13.8, 9.8 Hz), 1.84 (1H, dd, *J* = 13.3, 8.8 Hz), 1.73 (1H, m), 1.66 (3H, s), 1.65 (3H, d, 1.2 Hz), 1.64 − 1.60 (2H, m), 1.60 − 1.53 (3H, m), 1.50 (1H, ddd, *J* = 14.1, 10.3, 7.3 Hz), 1.16 (3H, d, *J* = 6.2 Hz), 0.98 (3H, d, *J* = 6.7 Hz), 0.97 (3H, d, *J* = 6.8 Hz), 0.89 (3H, d, *J* = 6.8 Hz)

^{13}C NMR (125 MHz, CD_3OD): 176.13, 138.55, 134.43, 131.35, 123.91, 77.81, 76.55, 75.76, 68.45, 47.32, 44.80, 44.46, 40.95, 36.67, 36.13, 35.68, 35.52, 30.22, 23.66, 21.47, 19.56, 17.44, 16.43, 16.21, 15.28 (Reference MeOD at 49.15)

$[\alpha]_D^{24.2}$ = +55 (c = 1.0, $CHCl_3$)

HMRS (ESI$^+$): calcd. for $C_{25}H_{44}O_5Na$ (M+Na): 447.3081, found 447.3067

Data in accordance with: A. B. Benowitz, S. Fidanze, P. L. C. Small, Y. Kishi, *J. Am. Chem. Soc.* **2001,** *123,* 5128 – 5129; **and** G. Wang, Y. Ning, E.–i. Negishi, *Chem. Eur. J,* **2011**, *17,* 4118

8 ^{13}C Chemical Shift Comparison

Table 1. NMR Data comparison of Mycolactyone Core **2**

^{13}C Data referenced to C19 at 67.10 (chiral centre derived from (*R*)-3-hydroxybutyrate). The resultant CD_3OD peak (47.84) matches that in the ^{13}C spectrum of the Negishi synthesis

Chemical Shift / ppm				
This Work	Δ	Kishi	Δ	Negishi
**174.78**	−0.09	**174.87**	−0.09	**174.87**
**137.19**	−0.07	**137.26**	−0.06	**137.25**
**133.07**	−0.08	**133.15**	−0.06	**133.13**
**129.99**	−0.04	**130.03**	−0.05	**130.04**
**122.55**	−0.05	**122.60**	−0.05	**122.60**
**76.46**	−0.03	**76.49**	−0.07	**76.53**
**75.20**	0.00	**75.20**	−0.04	**75.24**
**74.40**	−0.11	**74.51**	−0.05	**74.45**
**67.10**	0.00	**67.10**	−0.03	**67.13**
**CD_3OD (47.84)**	–	**–**	–	**47.84**
**45.97**	−0.03	**46.00**	−0.04	**46.01**
**43.44**	−0.05	**43.49**	−0.02	**43.46**
**43.10**	−0.08	**43.18**	−0.05	**43.15**
**39.60**	−0.06	**39.66**	−0.04	**39.64**
**35.32**	−0.05	**35.37**	−0.03	**35.35**
**34.77**	−0.03	**34.80**	−0.04	**34.81**
**34.33**	−0.02	**34.35**	−0.03	**34.36**
**34.17**	−0.03	**34.20**	−0.03	**34.20**
**28.87**	−0.02	**28.89**	−0.02	**28.89**
**22.30**	+0.02	**22.28**	+0.01	**22.29**
**20.11**	−0.04	**20.15**	−0.05	**20.16**
**18.20**	−0.05	**18.25**	+0.01	**18.19**
**16.09**	−0.01	**16.10**	0.00	**16.09**
**15.08**	+0.02	**15.06**	0.00	**15.08**
**14.86**	+0.01	**14.85**	+0.01	**14.85**
**13.93**	+0.01	**13.92**	+0.00	**13.93**

9 Determination of Stereochemical Purity

Carbamate 5

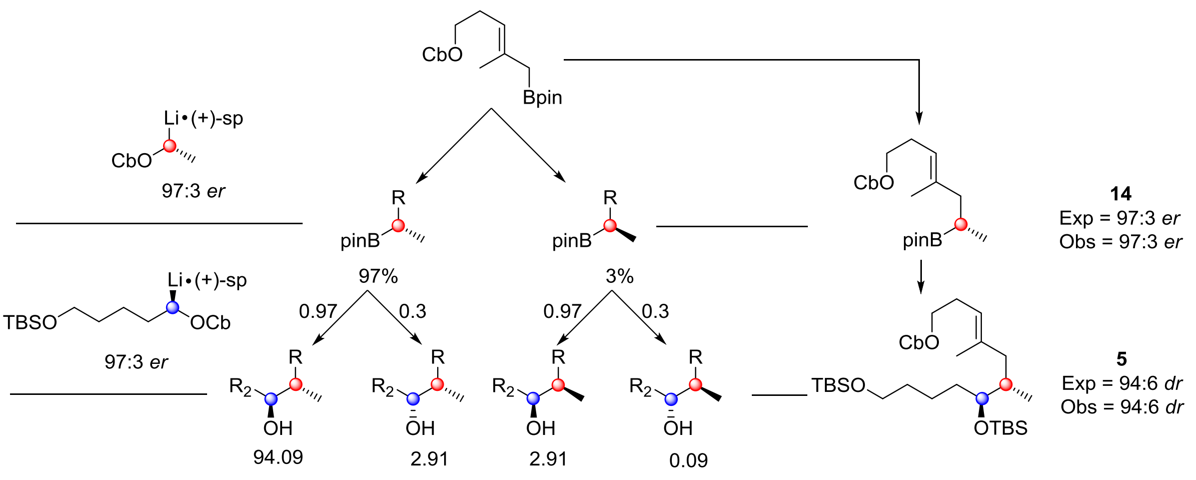

Calc. *er* = 94.09:0.09 = >99:1 *er*
Calc. *dr* = 94.09:5.91 = 94:6 *dr*

Determination of *dr*, *syn*:*anti*, 94:6

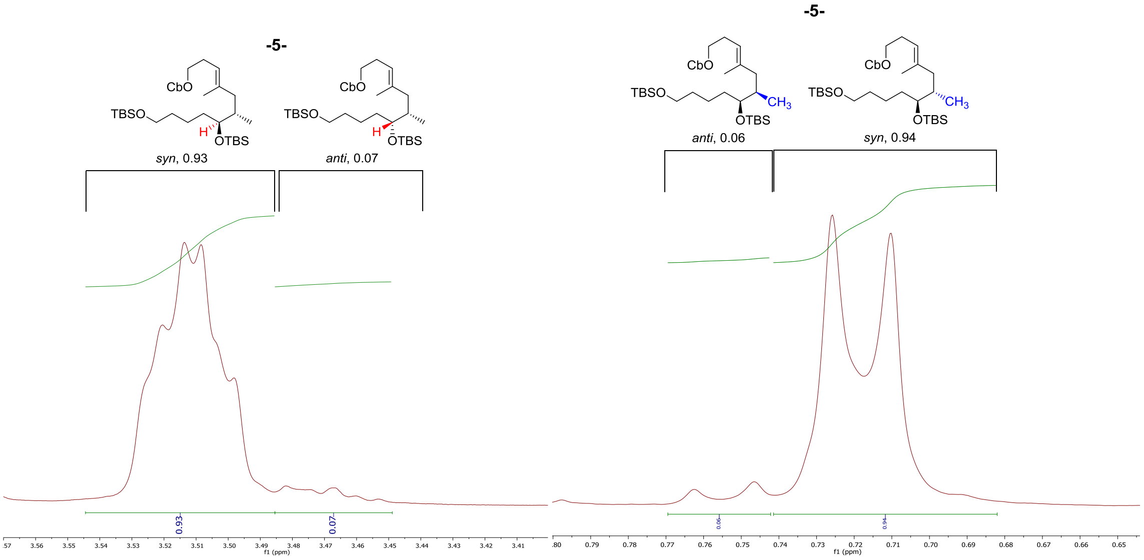

Pinacol Boronic Ester 5

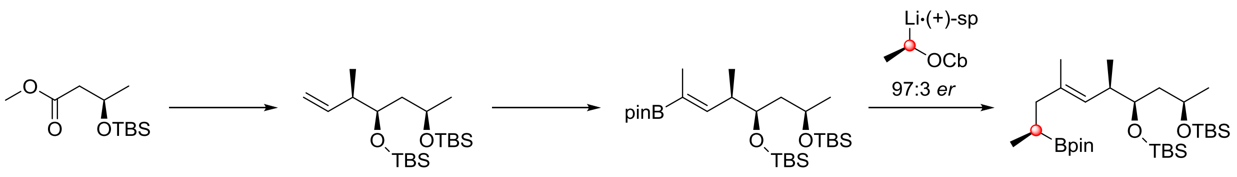

SI-8	8	20	4
>99:1 *er*	>99:1 *er*	>99:1 *er*	>99:1 *er*
	94:6 *dr*	>99:1 Z/E	97:3 *dr*
		purified to 99:1 *dr*	

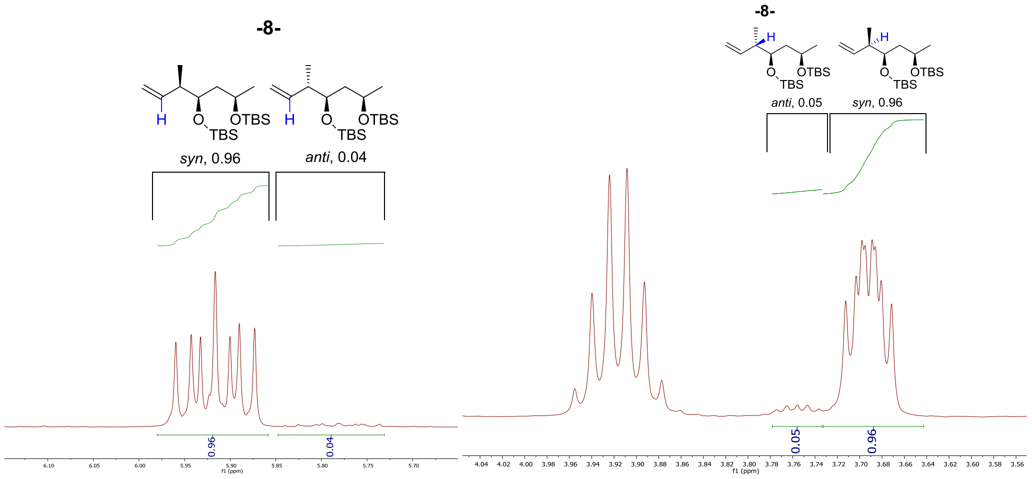

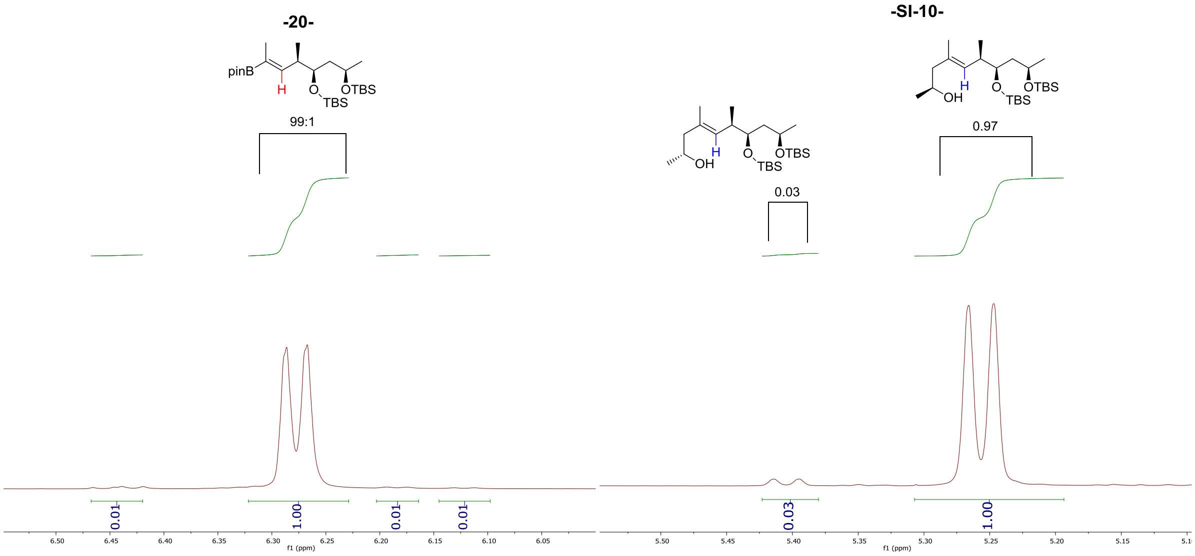

Known intermediate 3

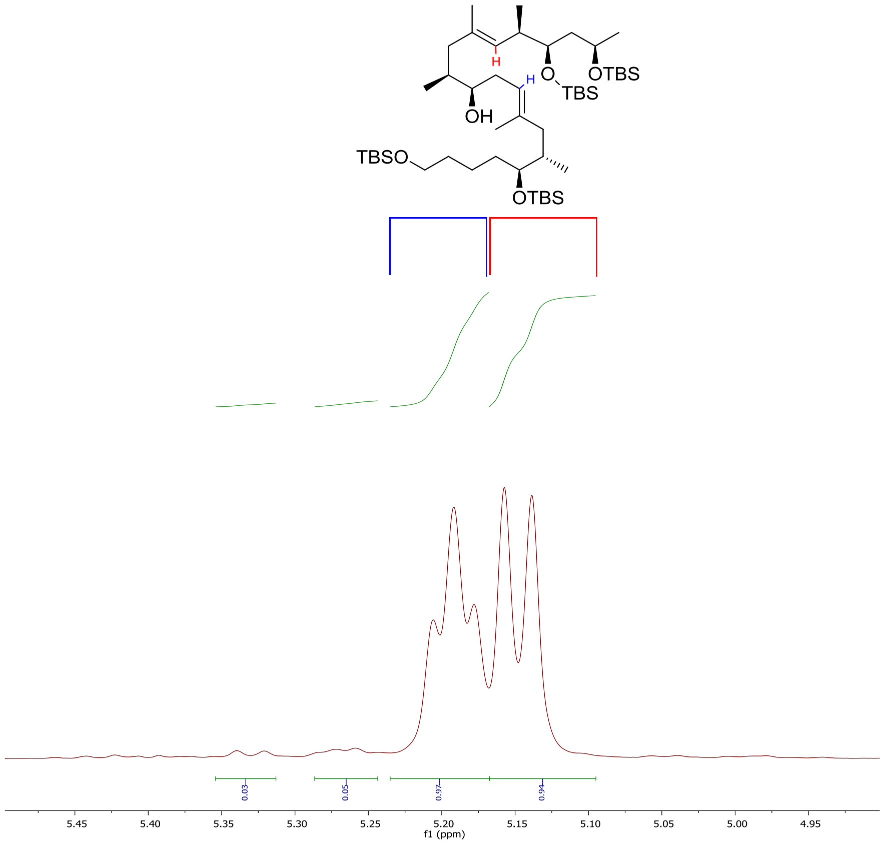

Mycolactone Core 2

Enlarged view of ^{1}H spectrum for the methyl groups of Core **2**:

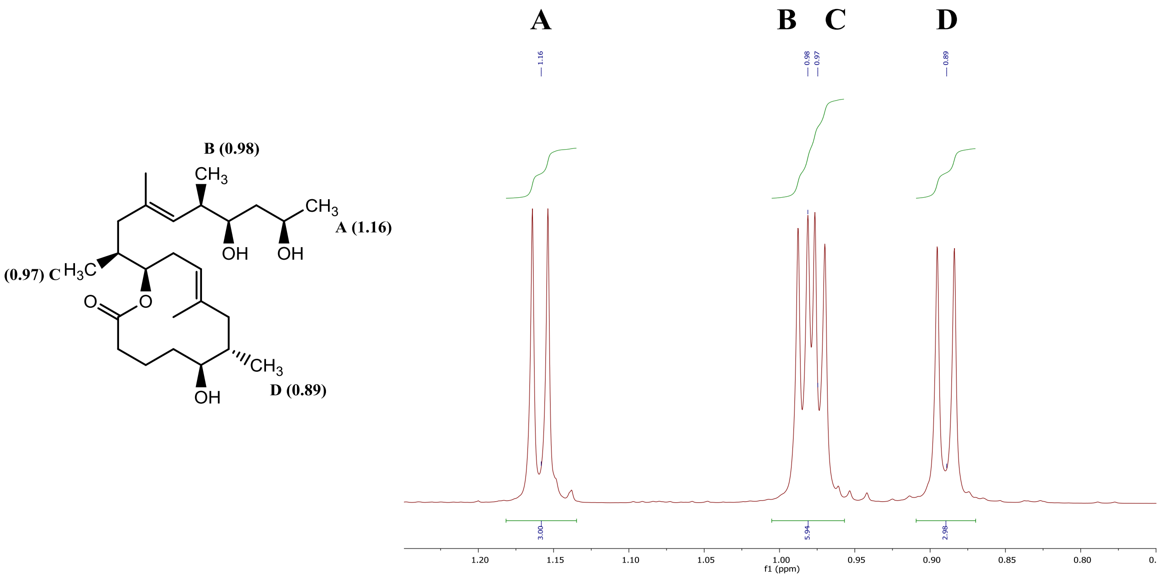

Enlarged view of ^{13}C spectrum of Core **2**:

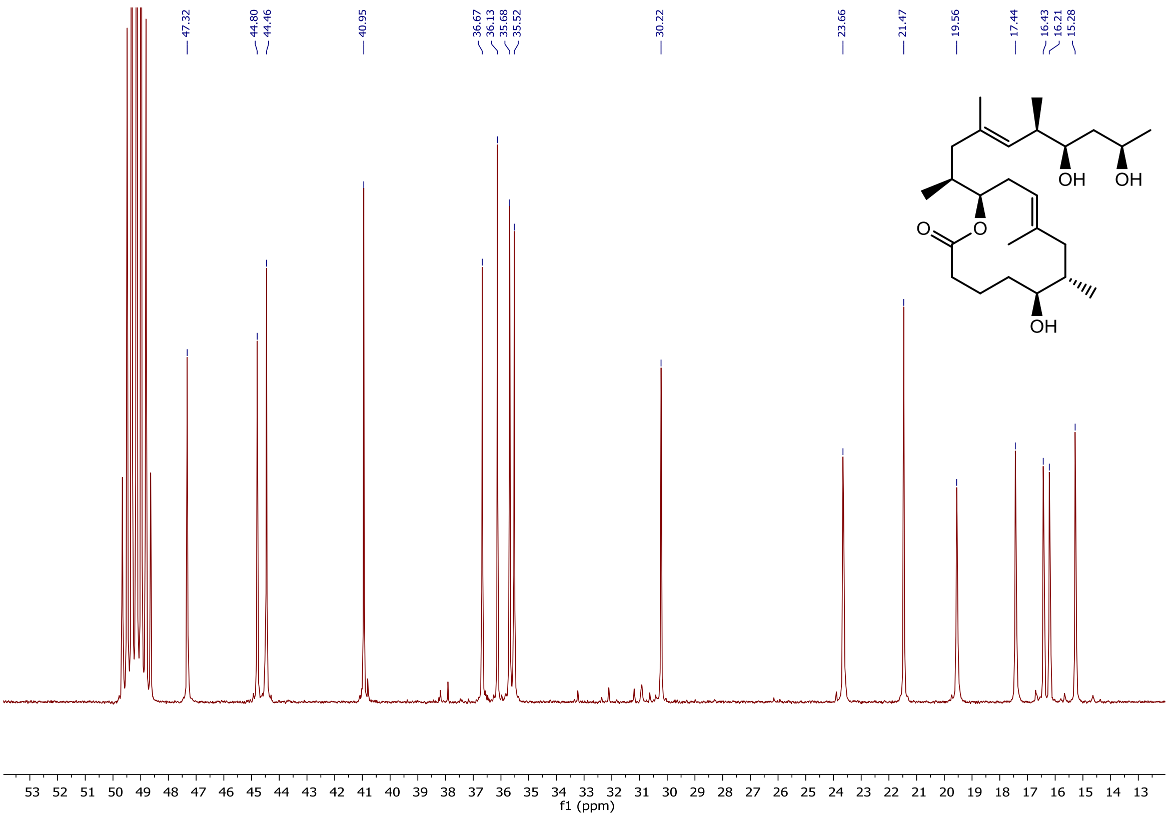

10 ^{1}H and ^{13}C NMR Spectra

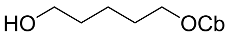

SI-2

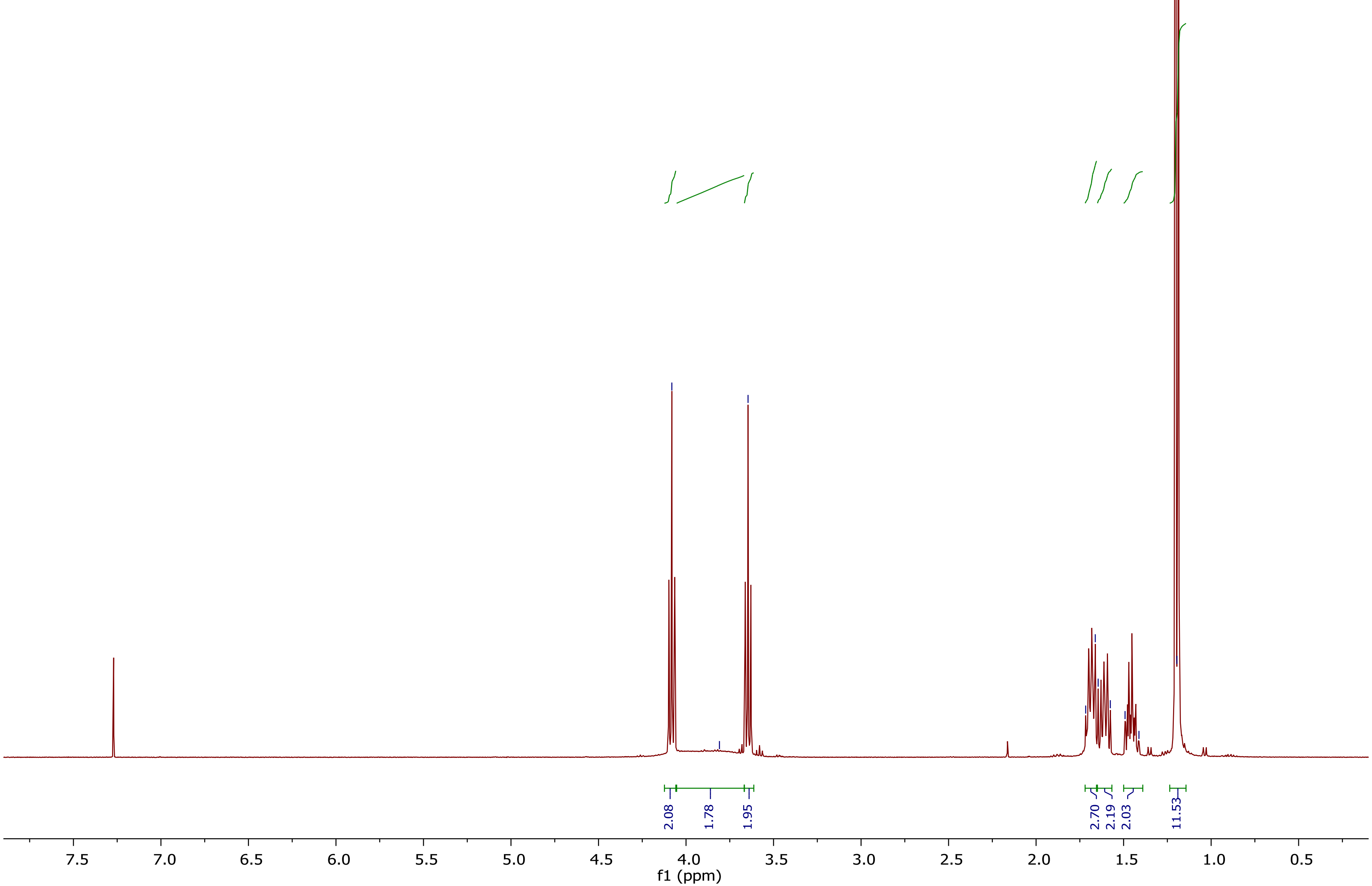
4.08
3.81
3.65
1.72
1.66
1.65
1.58
1.49
1.41
1.20
2.08
1.78
1.95
2.70
2.19
2.03
11.53
7.5
7.0
6.5
6.0
5.5
5.0
4.5
4.0
3.5
3.0
2.5
2.0
1.5
1.0
0.5
f1 (ppm)

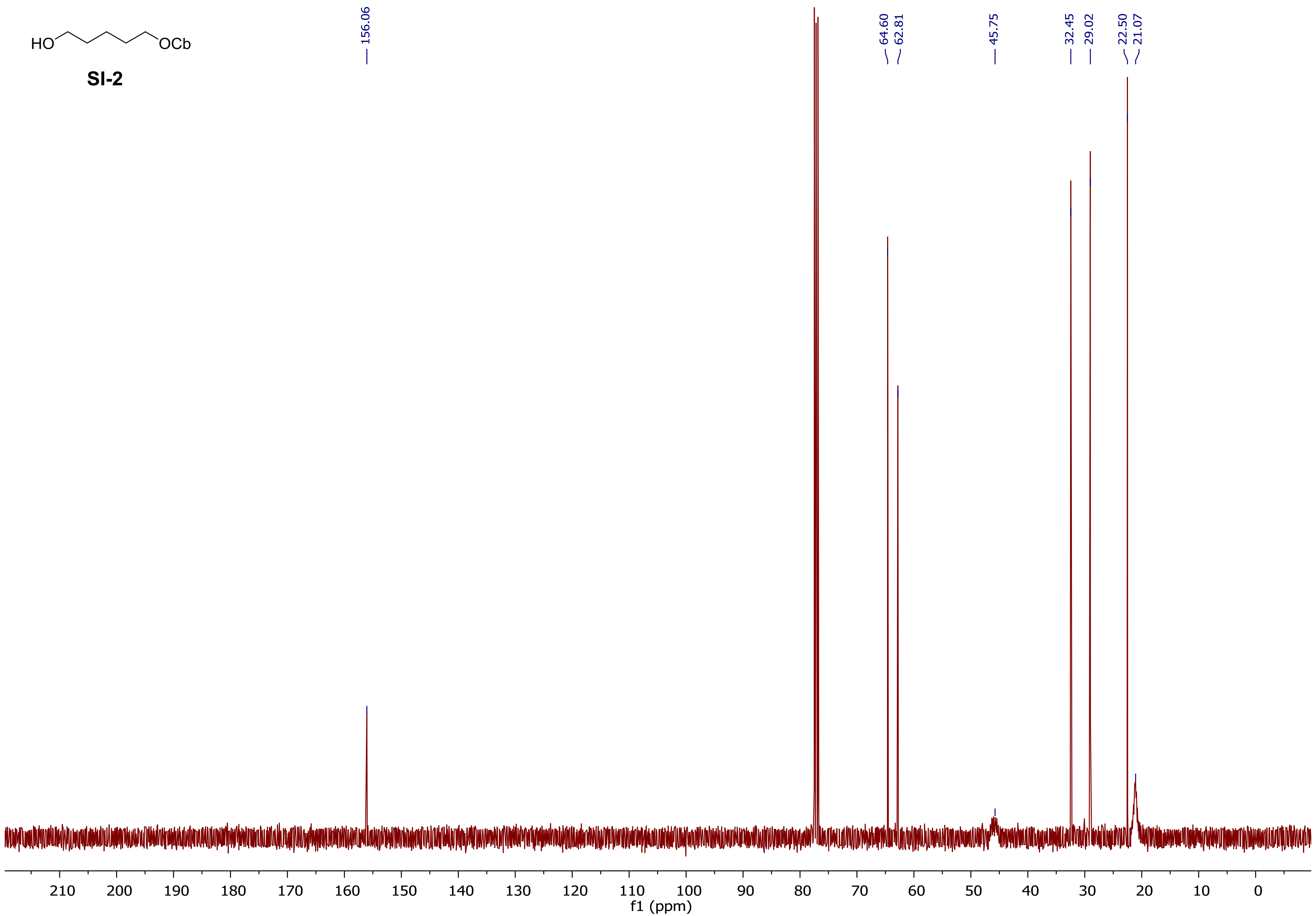
HO
OCb
SI-2
156.06
64.60
62.81
45.75
32.45
29.02
22.50
21.07
210
200
190
180
170
160
150
140
130
120
110
100
90
80
70
60
50
40
30
20
10
0
f1 (ppm)

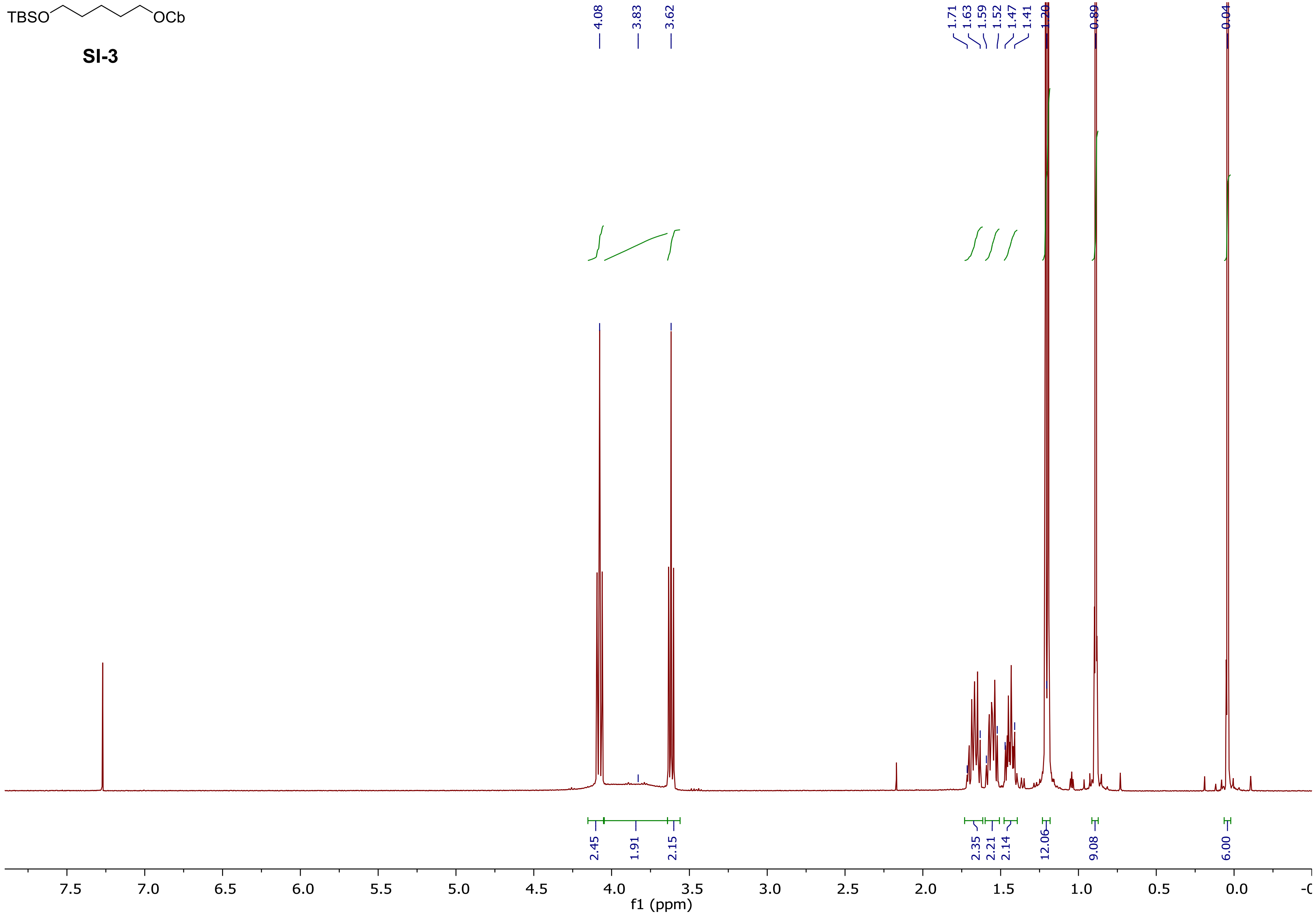
TBSO
OCb
SI-3
4.08
3.83
3.62
1.71
1.63
1.59
1.52
1.47
1.41
1.20
0.89
0.04
2.45
1.91
2.15
2.35
2.21
2.14
12.06
9.08
6.00
7.5
7.0
6.5
6.0
5.5
5.0
4.5
4.0
3.5
3.0
2.5
2.0
1.5
1.0
0.5
0.0
f1 (ppm)

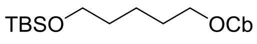
TBSO
OCb

SI-3

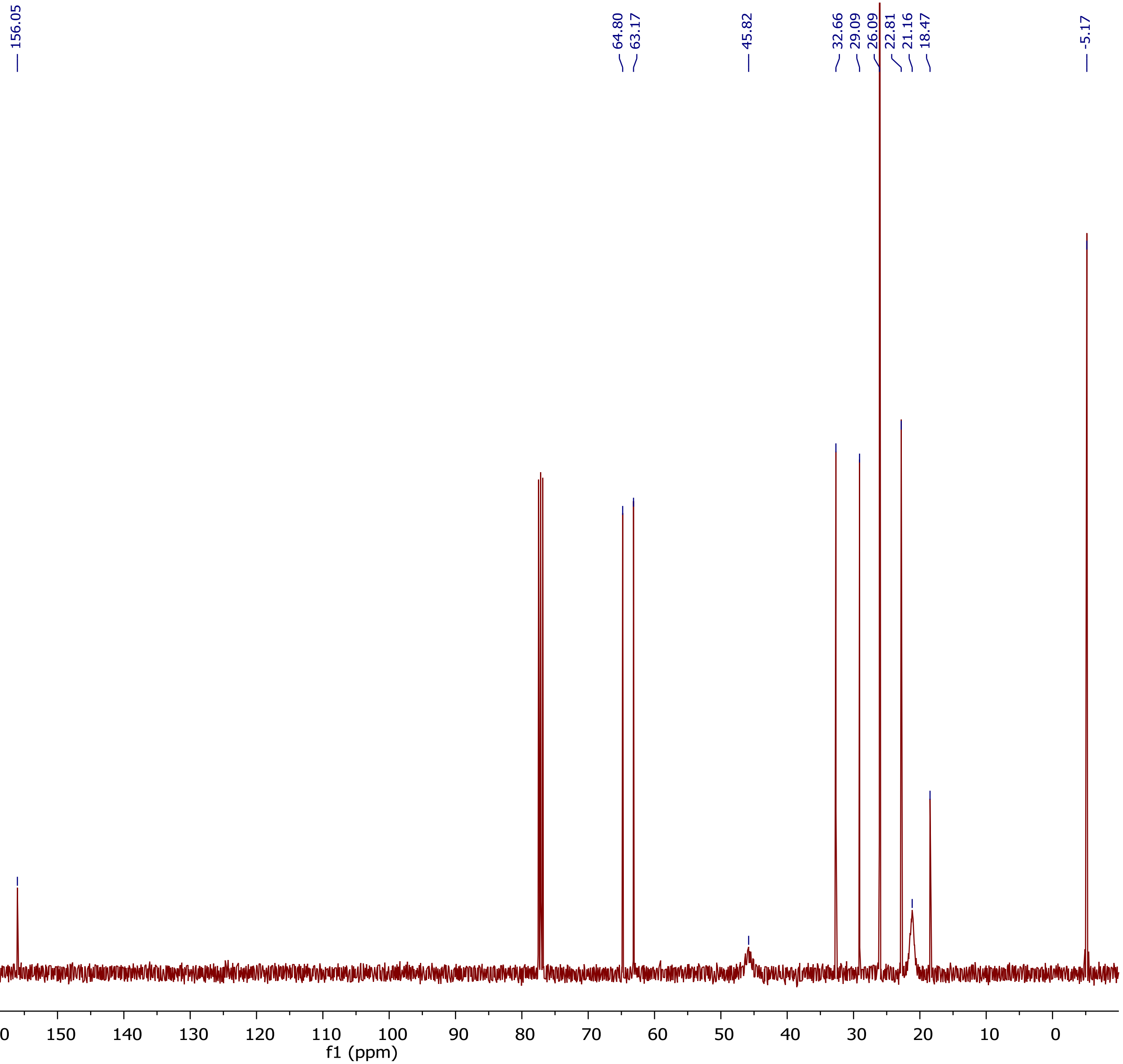
156.05
64.80
63.17
45.82
32.66
29.09
26.09
22.81
21.16
18.47
-5.17
210
200
190
180
170
160
150
140
130
120
110
100
90
80
70
60
50
40
30
20
10
0
f1 (ppm)

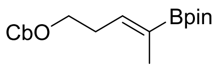
CbO
Bpin

12

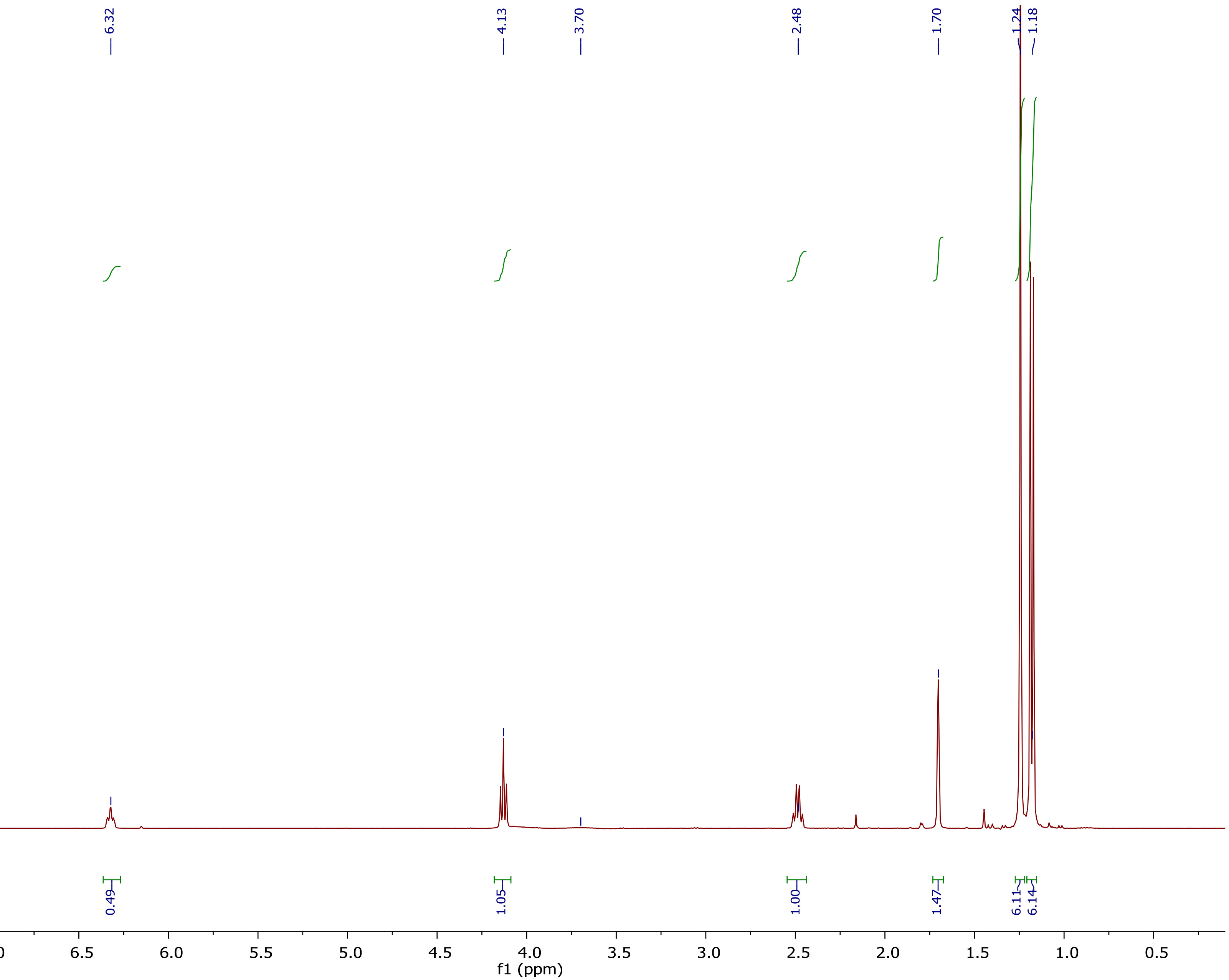
6.32
4.13
3.70
2.48
1.70
1.24
1.18
0.49
1.05
1.00
1.47
6.11
6.14
7.5
7.0
6.5
6.0
5.5
5.0
4.5
4.0
3.5
3.0
2.5
2.0
1.5
1.0
0.5
f1 (ppm)

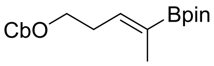
CbO
Bpin

12

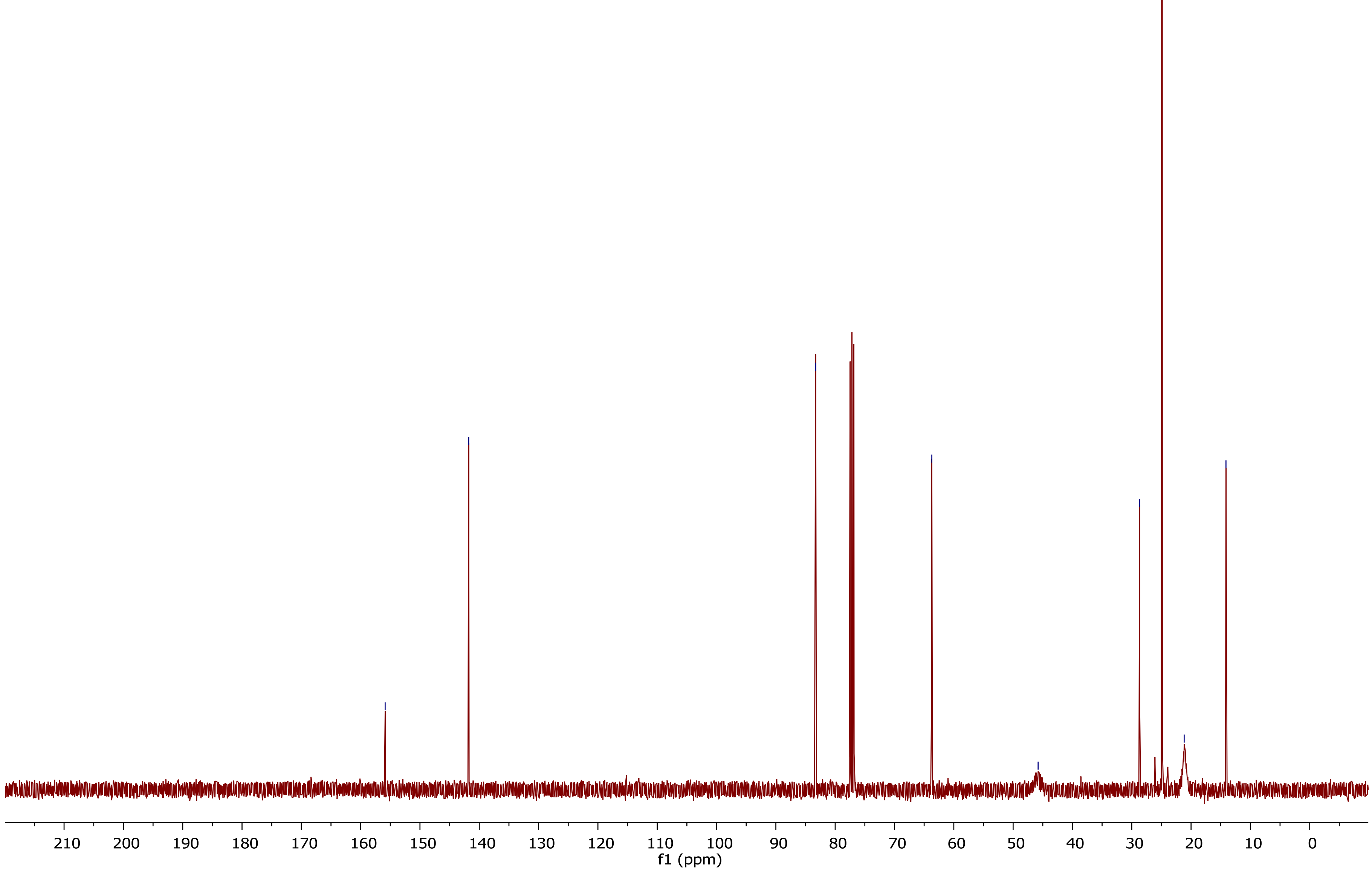
155.87
141.78
83.29
63.70
45.79
28.66
24.87
21.16
14.12
210
200
190
180
170
160
150
140
130
120
110
100
90
80
70
60
50
40
30
20
10
0
f1 (ppm)

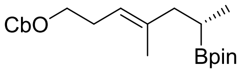

CbO
Bpin

14

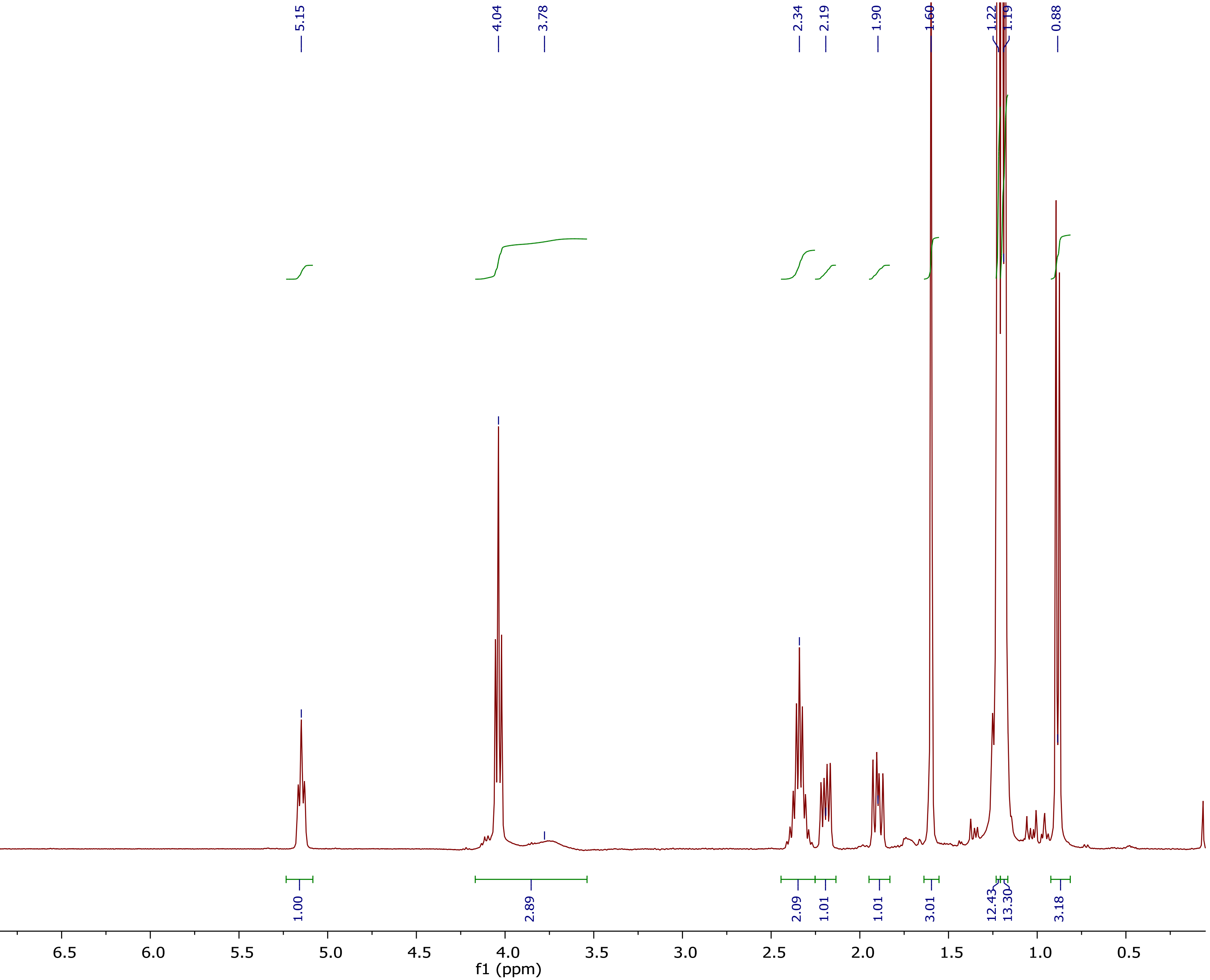

5.15
4.04
3.78
2.34
2.19
1.90
1.60
1.22
1.19
0.88
1.00
2.89
2.09
1.01
1.01
3.01
12.43
13.30
3.18
7.5
7.0
6.5
6.0
5.5
5.0
4.5
4.0
3.5
3.0
2.5
2.0
1.5
1.0
0.5
f1 (ppm)

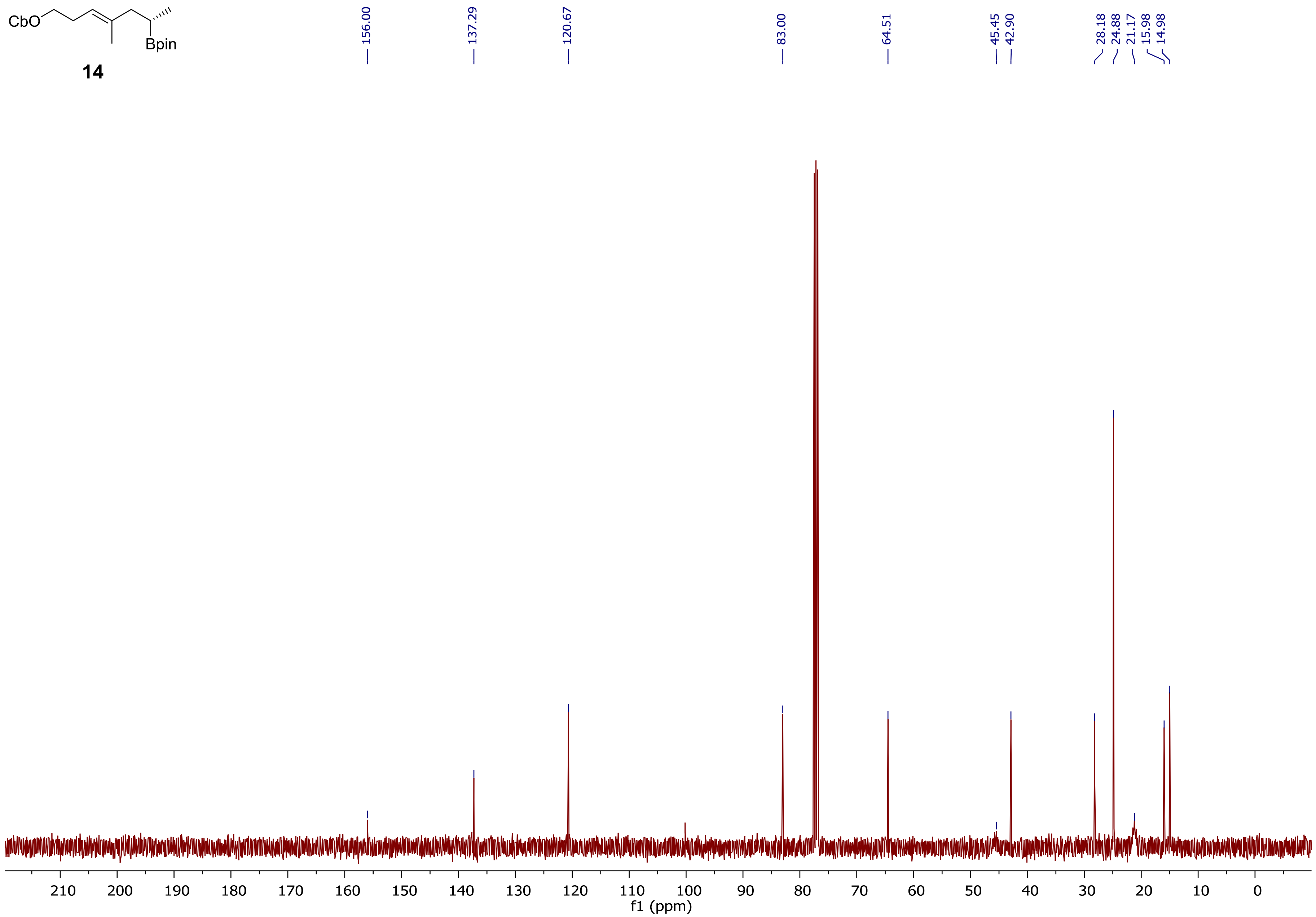
CbO
Bpin
14
156.00
137.29
120.67
83.00
64.51
45.45
42.90
28.18
24.88
21.17
15.98
14.98
210 200 190 180 170 160 150 140 130 120 110 100 90 80 70 60 50 40 30 20 10 0
f1 (ppm)

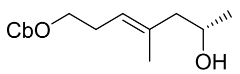

SI-5

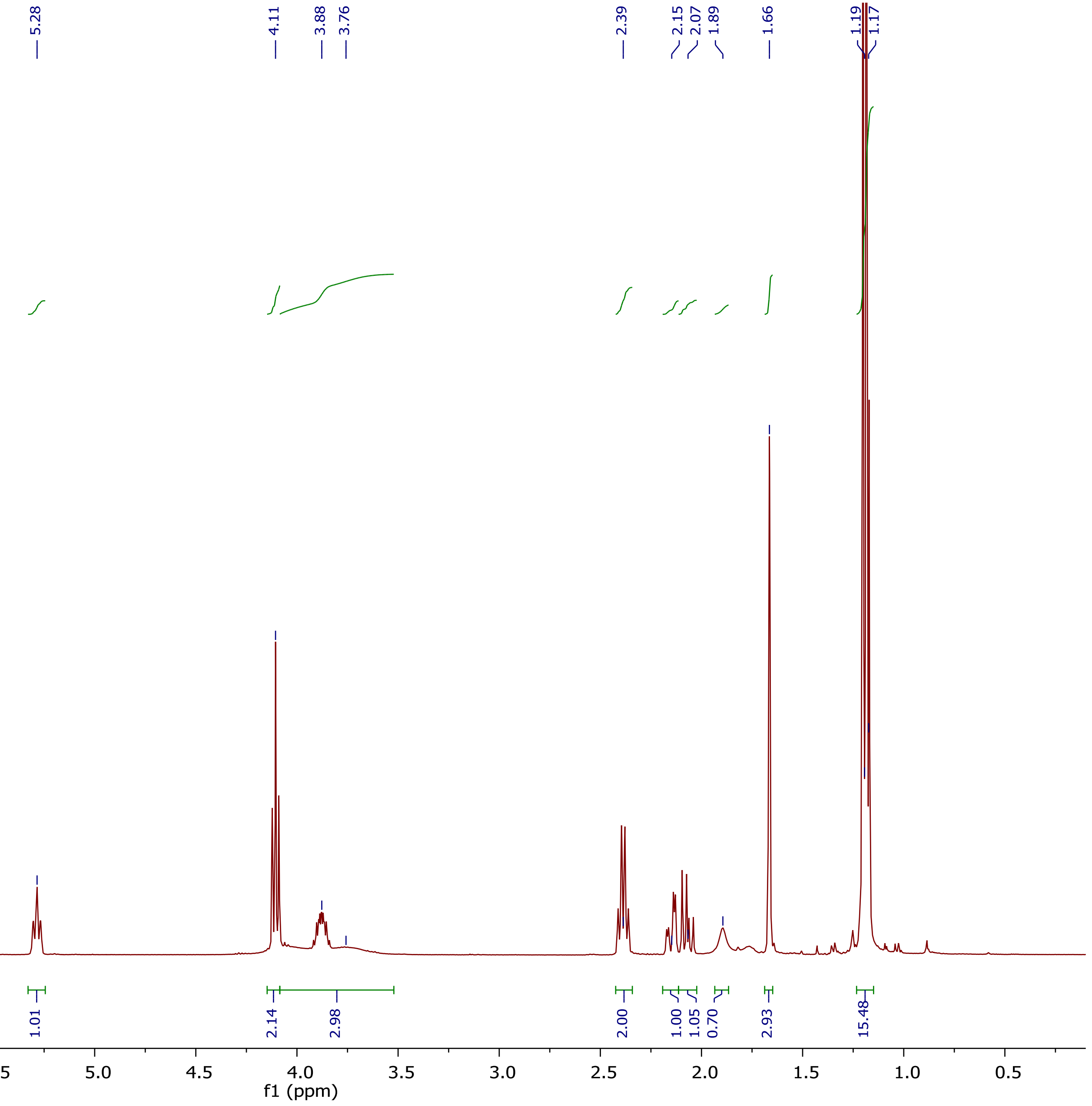

5.28
4.11
3.88
3.76
2.39
2.15
2.07
1.89
1.66
1.19
1.17
1.01
2.14
2.98
2.00
1.00
1.05
0.70
2.93
15.48
7.5
7.0
6.5
6.0
5.5
5.0
4.5
4.0
3.5
3.0
2.5
2.0
1.5
1.0
0.5
f1 (ppm)

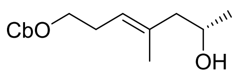

SI-5

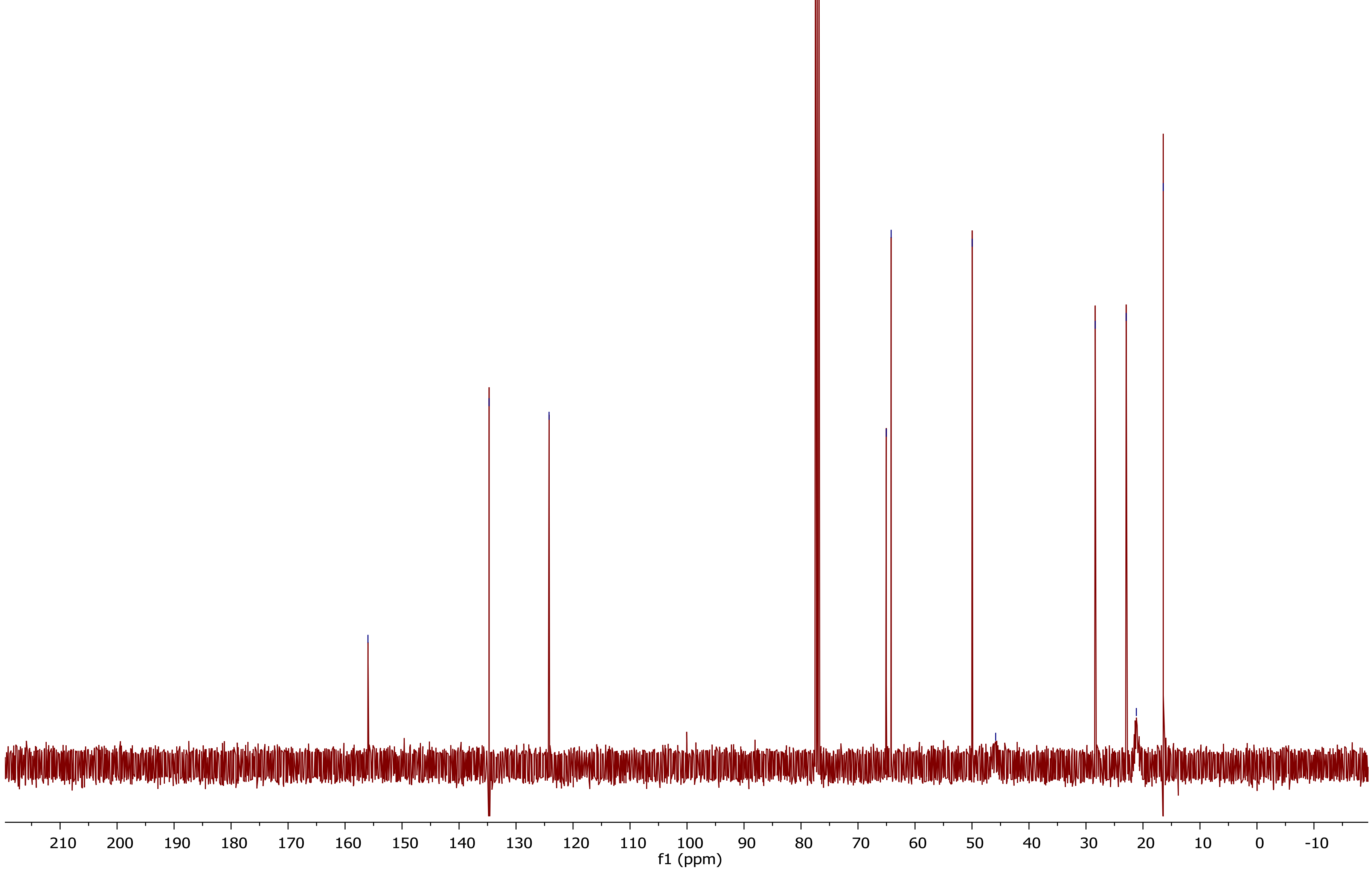

155.99
134.73
124.21
65.04
64.18
49.97
45.85
28.38
22.95
21.16
16.45
210 200 190 180 170 160 150 140 130 120 110 100 90 80 70 60 50 40 30 20 10 0 -10
f1 (ppm)

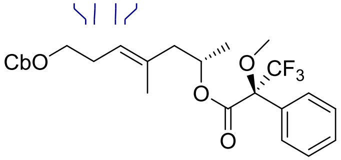

SI-6

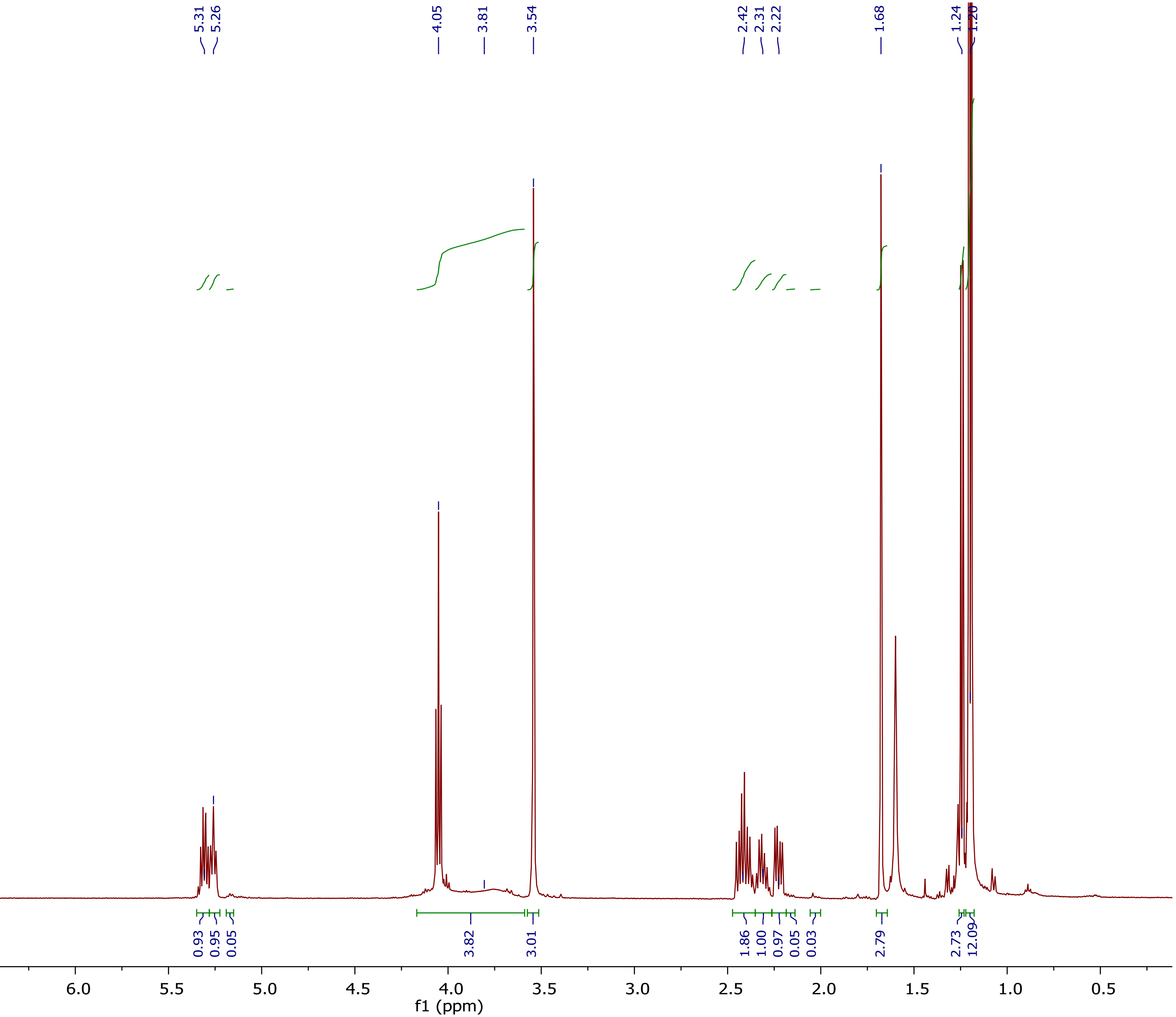
7.54
7.50
7.41
7.37
5.31
5.26
4.05
3.81
3.54
2.42
2.31
2.22
1.68
1.24
1.20
2.00
3.03
0.93
0.95
0.05
3.82
3.01
1.86
1.00
0.97
0.05
0.03
2.79
2.73
12.09
7.5
7.0
6.5
6.0
5.5
5.0
4.5
4.0
3.5
3.0
2.5
2.0
1.5
1.0
0.5
f1 (ppm)

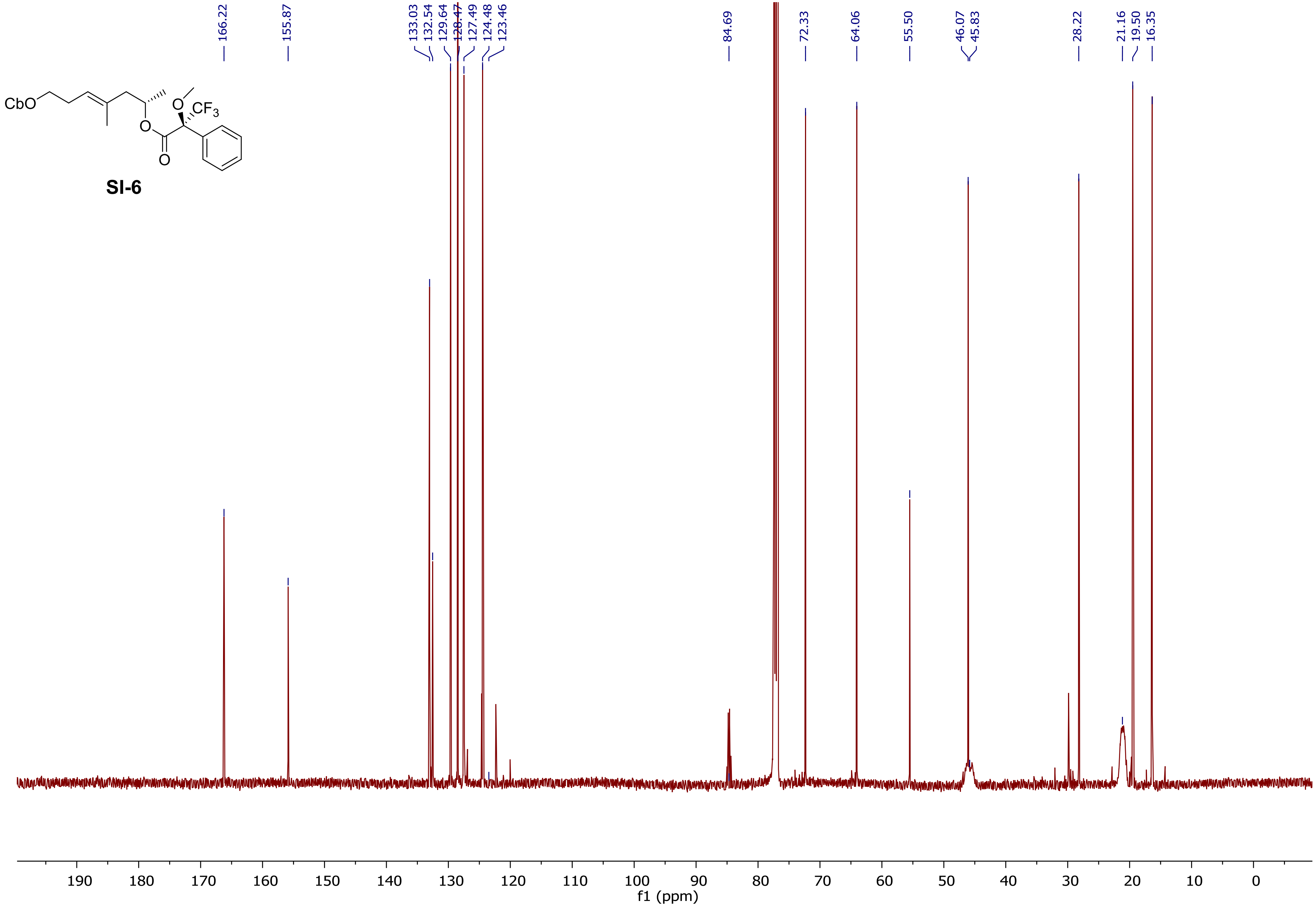
SI-6
166.22
155.87
133.03
132.54
129.64
128.47
127.49
124.48
123.46
84.69
72.33
64.06
55.50
46.07
45.83
28.22
21.16
19.50
16.35
190 180 170 160 150 140 130 120 110 100 90 80 70 60 50 40 30 20 10 0
f1 (ppm)

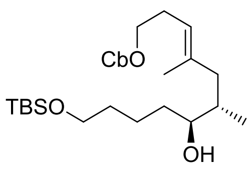

CbO
TBSO
OH

15

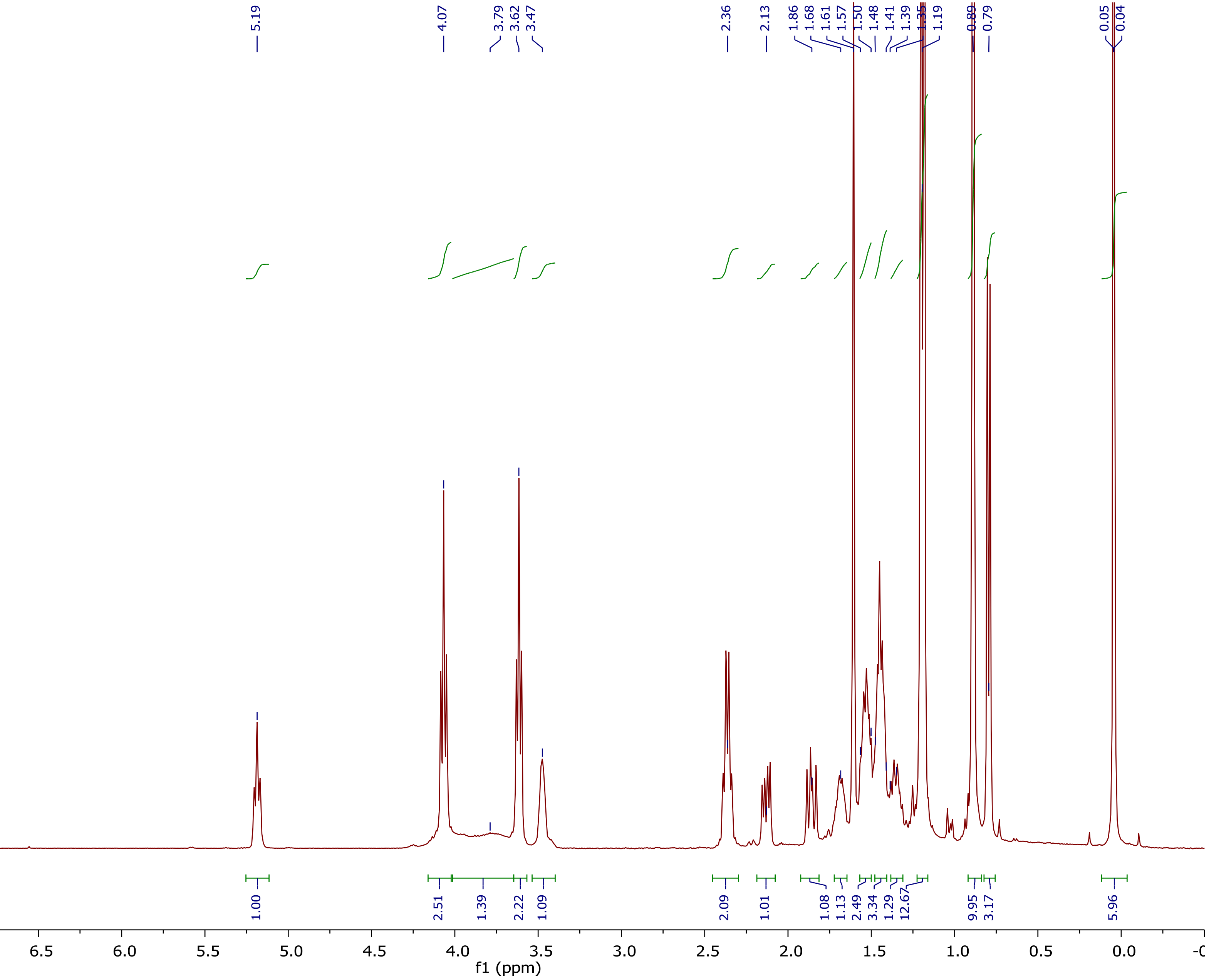

5.19
4.07
3.79
3.62
3.47
2.36
2.13
1.86
1.68
1.61
1.57
1.50
1.48
1.41
1.39
1.35
1.19
0.89
0.79
0.05
0.04
1.00
2.51
1.39
2.22
1.09
2.09
1.01
1.08
1.13
2.49
3.34
1.29
12.67
9.95
3.17
5.96
7.5
7.0
6.5
6.0
5.5
5.0
4.5
4.0
3.5
3.0
2.5
2.0
1.5
1.0
0.5
0.0
f1 (ppm)

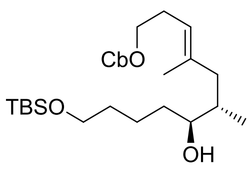

15

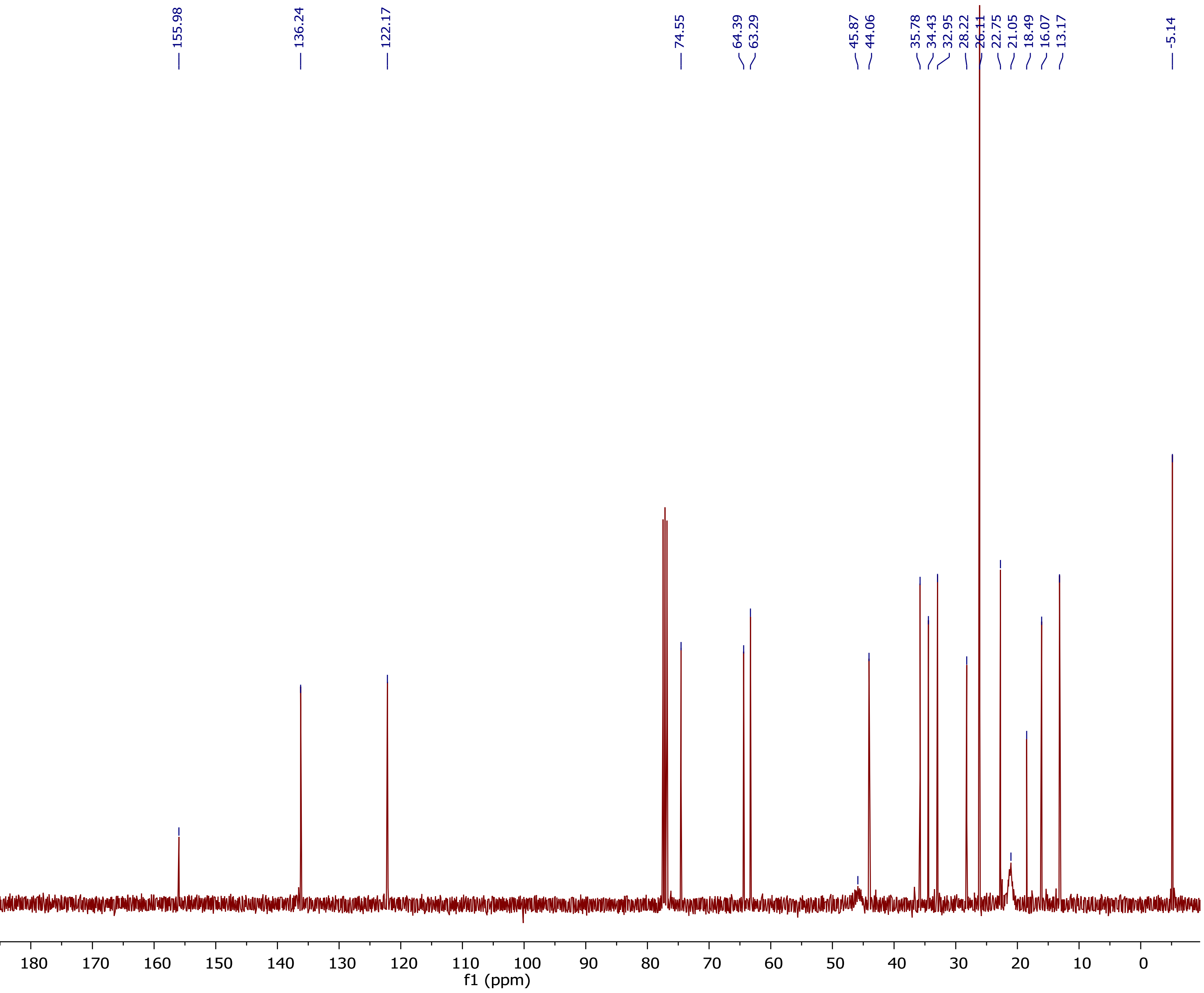
155.98
136.24
122.17
74.55
64.39
63.29
45.87
44.06
35.78
34.43
32.95
28.22
26.11
22.75
21.05
18.49
16.07
13.17
-5.14
210 200 190 180 170 160 150 140 130 120 110 100 90 80 70 60 50 40 30 20 10 0
f1 (ppm)

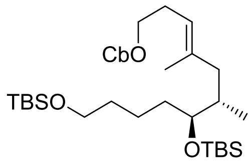
CbO
TBSO
OTBS

5

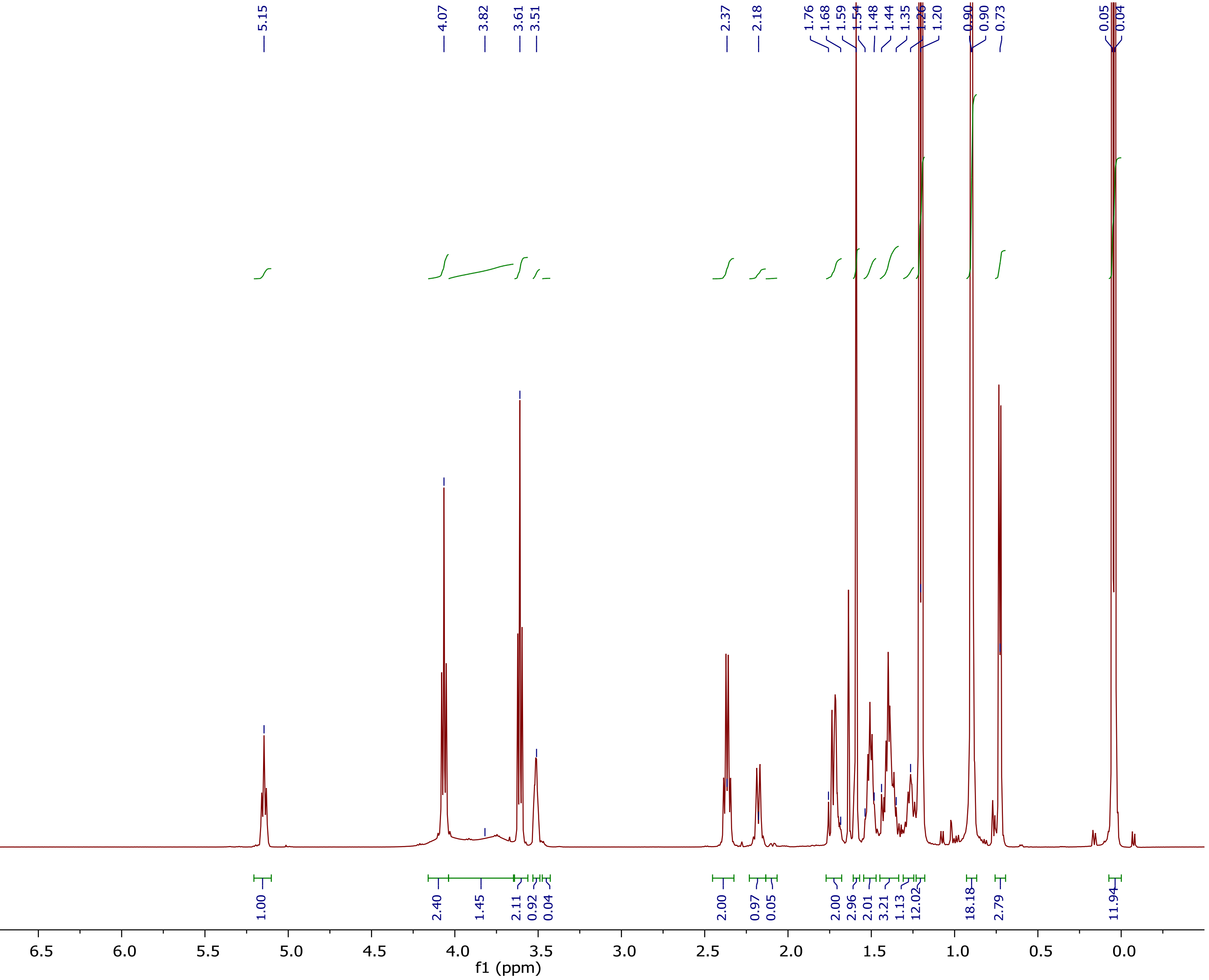
5.15
4.07
3.82
3.61
3.51
2.37
2.18
1.76
1.68
1.59
1.54
1.48
1.44
1.35
1.26
1.20
0.90
0.90
0.73
0.05
0.04
1.00
2.40
1.45
2.11
0.92
0.04
2.00
0.97
0.05
2.00
2.96
2.01
3.21
1.13
12.02
18.18
2.79
11.94
7.5
7.0
6.5
6.0
5.5
5.0
4.5
4.0
3.5
3.0
2.5
2.0
1.5
1.0
0.5
0.0
f1 (ppm)

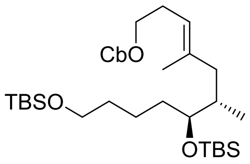
CbO
TBSO
OTBS

5

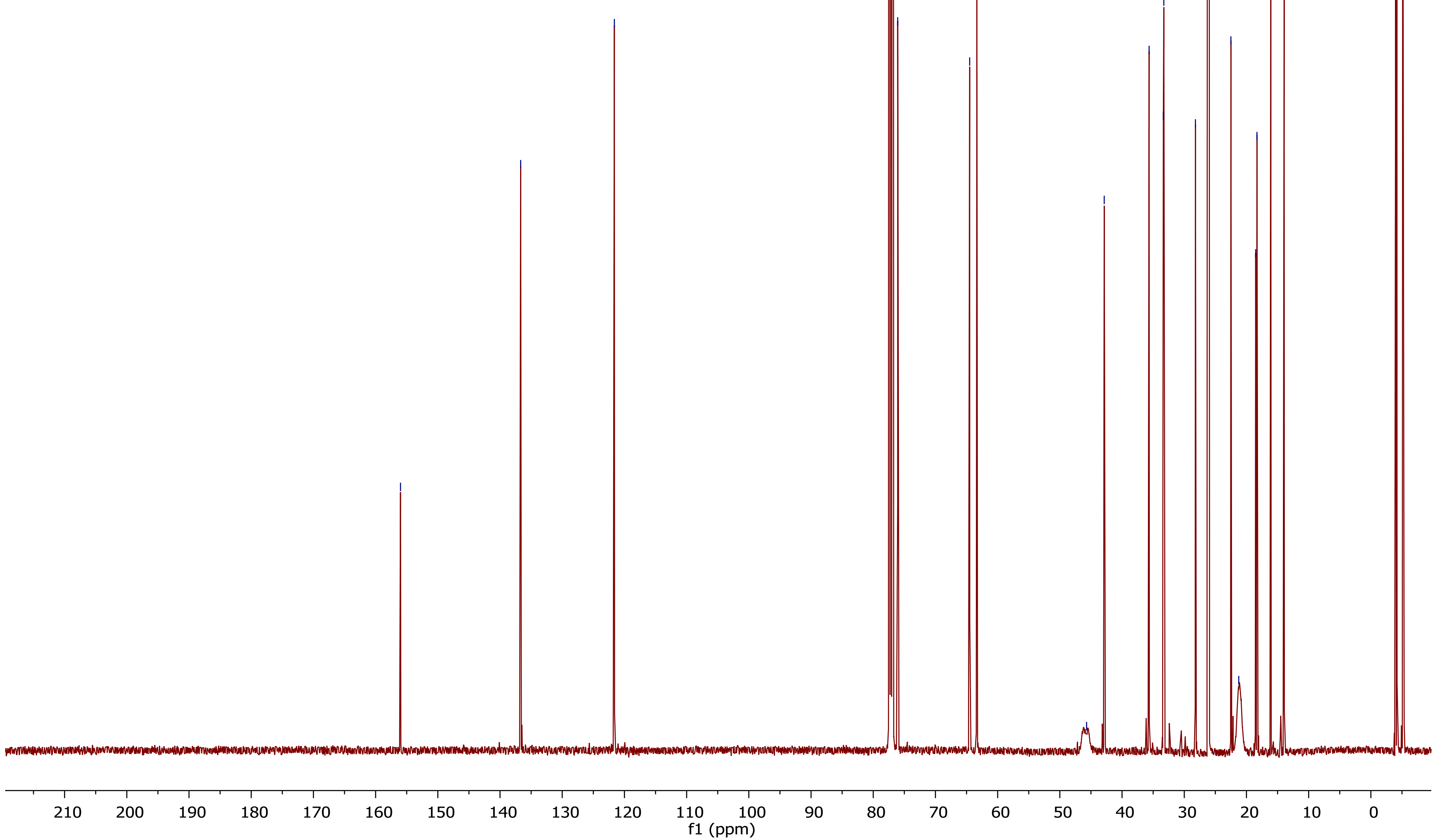
156.00
136.68
121.62
76.07
64.50
63.33
45.70
42.87
35.65
33.36
33.29
28.21
26.13
22.50
21.24
18.52
18.32
16.10
13.94
-3.99
-4.19
-5.13
210 200 190 180 170 160 150 140 130 120 110 100 90 80 70 60 50 40 30 20 10 0
f1 (ppm)

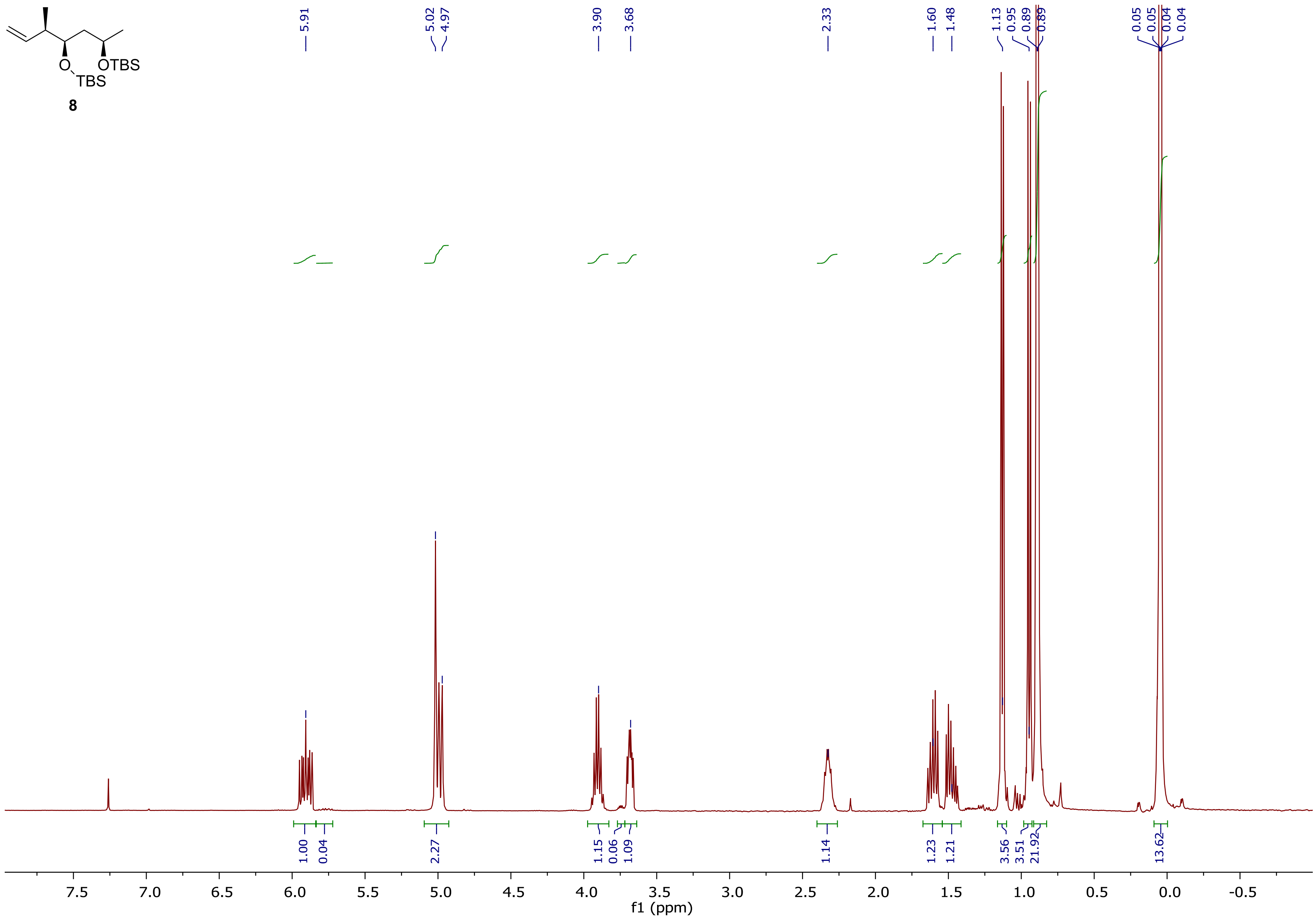

OTBS
O
TBS
8
5.91
5.02
4.97
3.90
3.68
2.33
1.60
1.48
1.13
0.95
0.89
0.89
0.05
0.05
0.04
0.04
1.00
0.04
2.27
1.15
0.06
1.09
1.14
1.23
1.21
3.56
3.51
21.92
13.62
7.5
7.0
6.5
6.0
5.5
5.0
4.5
4.0
3.5
3.0
2.5
2.0
1.5
1.0
0.5
0.0
-0.5
f1 (ppm)

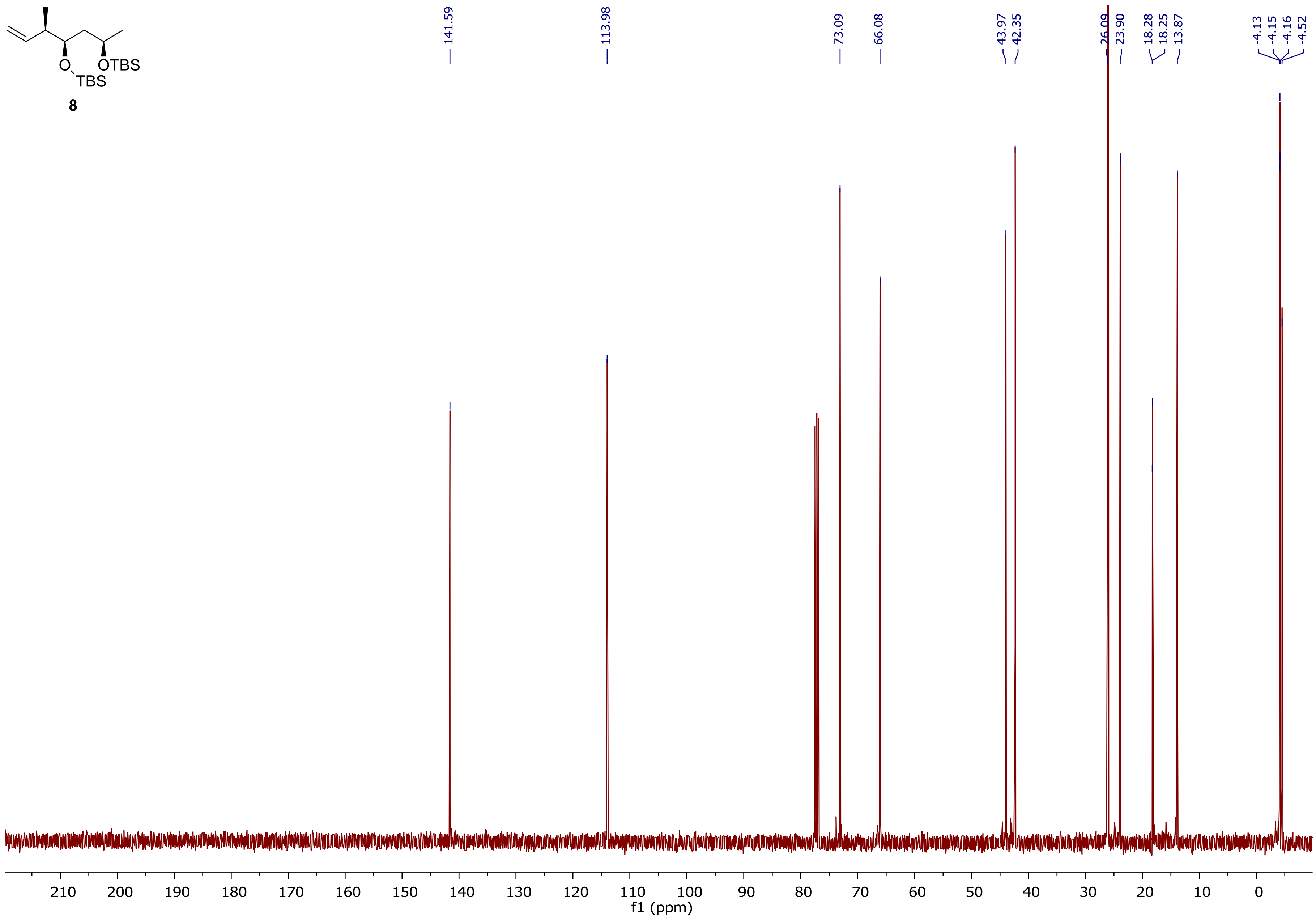

OTBS
O
TBS
8
141.59
113.98
73.09
66.08
43.97
42.35
26.09
23.90
18.28
18.25
13.87
-4.13
-4.15
-4.16
-4.52
210 200 190 180 170 160 150 140 130 120 110 100 90 80 70 60 50 40 30 20 10 0
f1 (ppm)

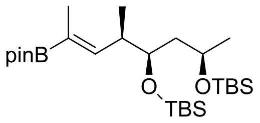
pinB
O
TBS
OTBS

20

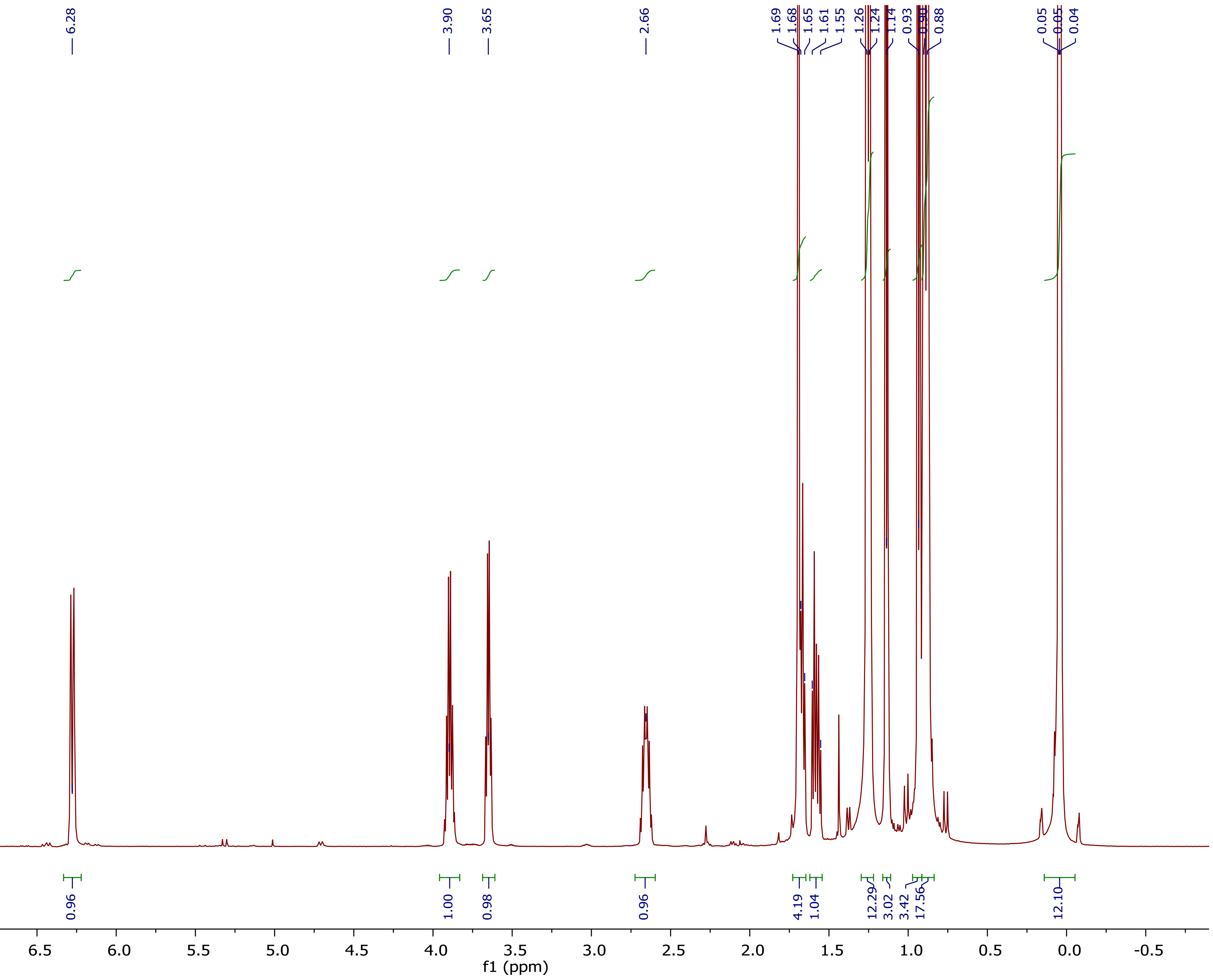
6.28
3.90
3.65
2.66
1.69
1.68
1.65
1.61
1.55
1.26
1.24
1.14
0.93
0.90
0.88
0.05
0.05
0.04
0.96
1.00
0.98
0.96
4.19
1.04
12.29
3.02
3.42
17.56
12.10
7.5
7.0
6.5
6.0
5.5
5.0
4.5
4.0
3.5
3.0
2.5
2.0
1.5
1.0
0.5
0.0
-0.5
f1 (ppm)

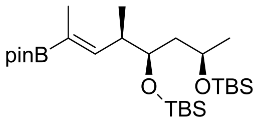

pinB
O
TBS
OTBS

20

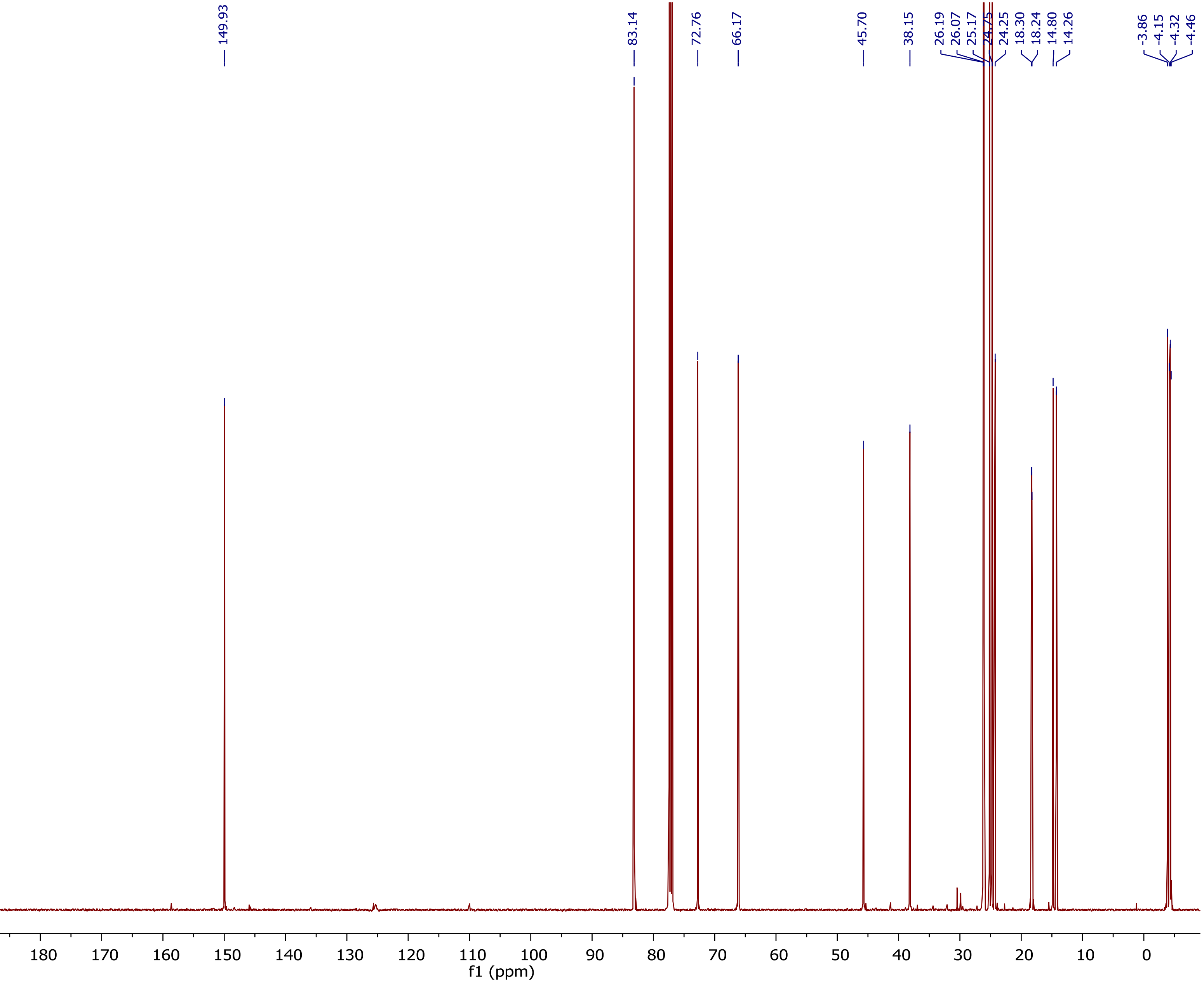

149.93
83.14
72.76
66.17
45.70
38.15
26.19
26.07
25.17
24.75
24.25
18.30
18.24
14.80
14.26
-3.86
-4.15
-4.32
-4.46
210 200 190 180 170 160 150 140 130 120 110 100 90 80 70 60 50 40 30 20 10 0
f1 (ppm)

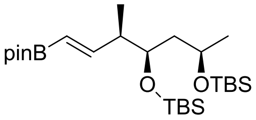
pinB
O
TBS
OTBS

21

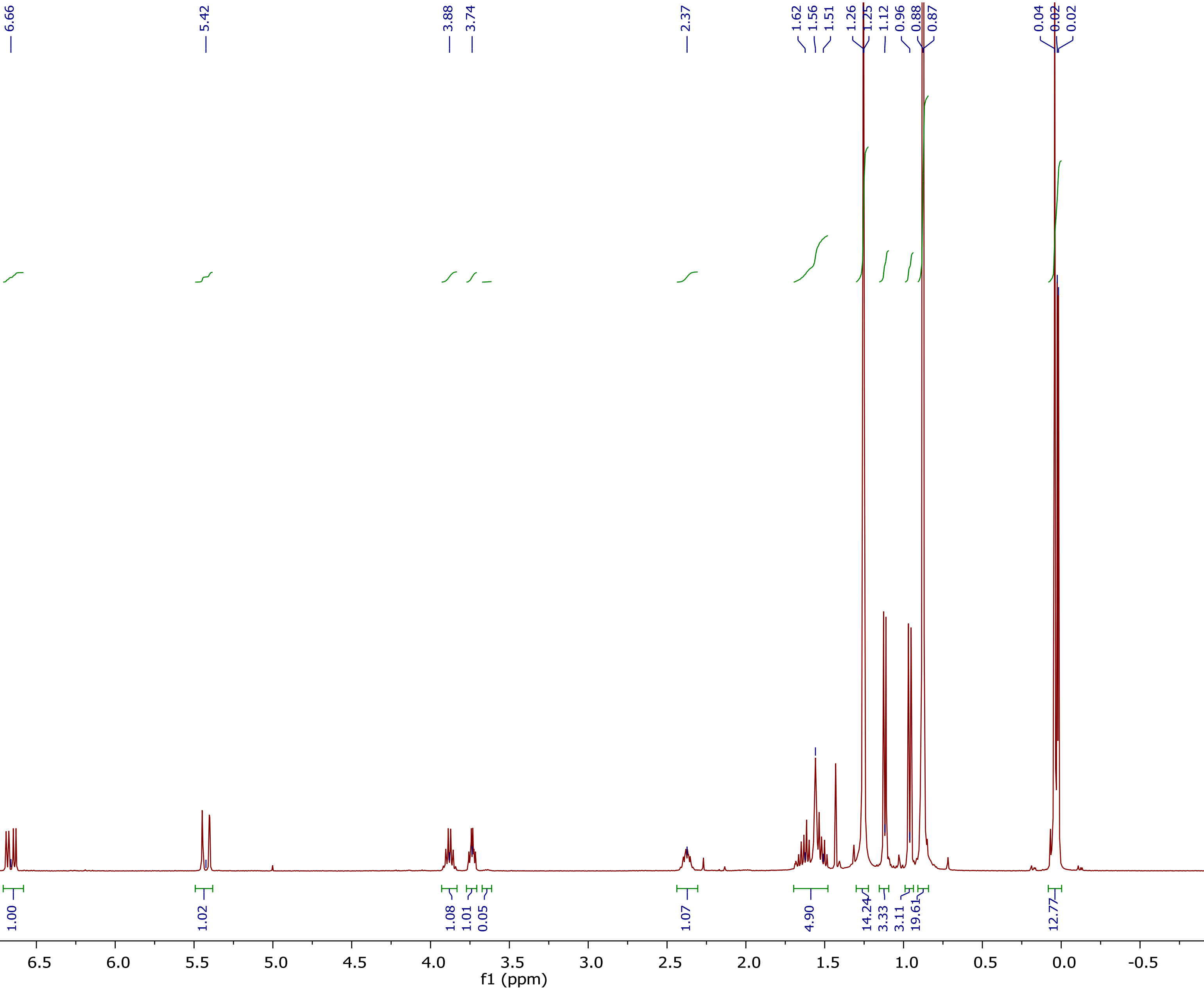
6.66
5.42
3.88
3.74
2.37
1.62
1.56
1.51
1.26
1.25
1.12
0.96
0.88
0.87
0.04
0.02
0.02
1.00
1.02
1.08
1.01
0.05
1.07
4.90
14.24
3.33
3.11
19.61
12.77
7.5
7.0
6.5
6.0
5.5
5.0
4.5
4.0
3.5
3.0
2.5
2.0
1.5
1.0
0.5
0.0
-0.5
f1 (ppm)

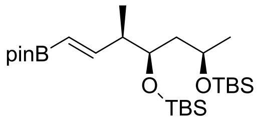
pinB
O
TBS
OTBS

21

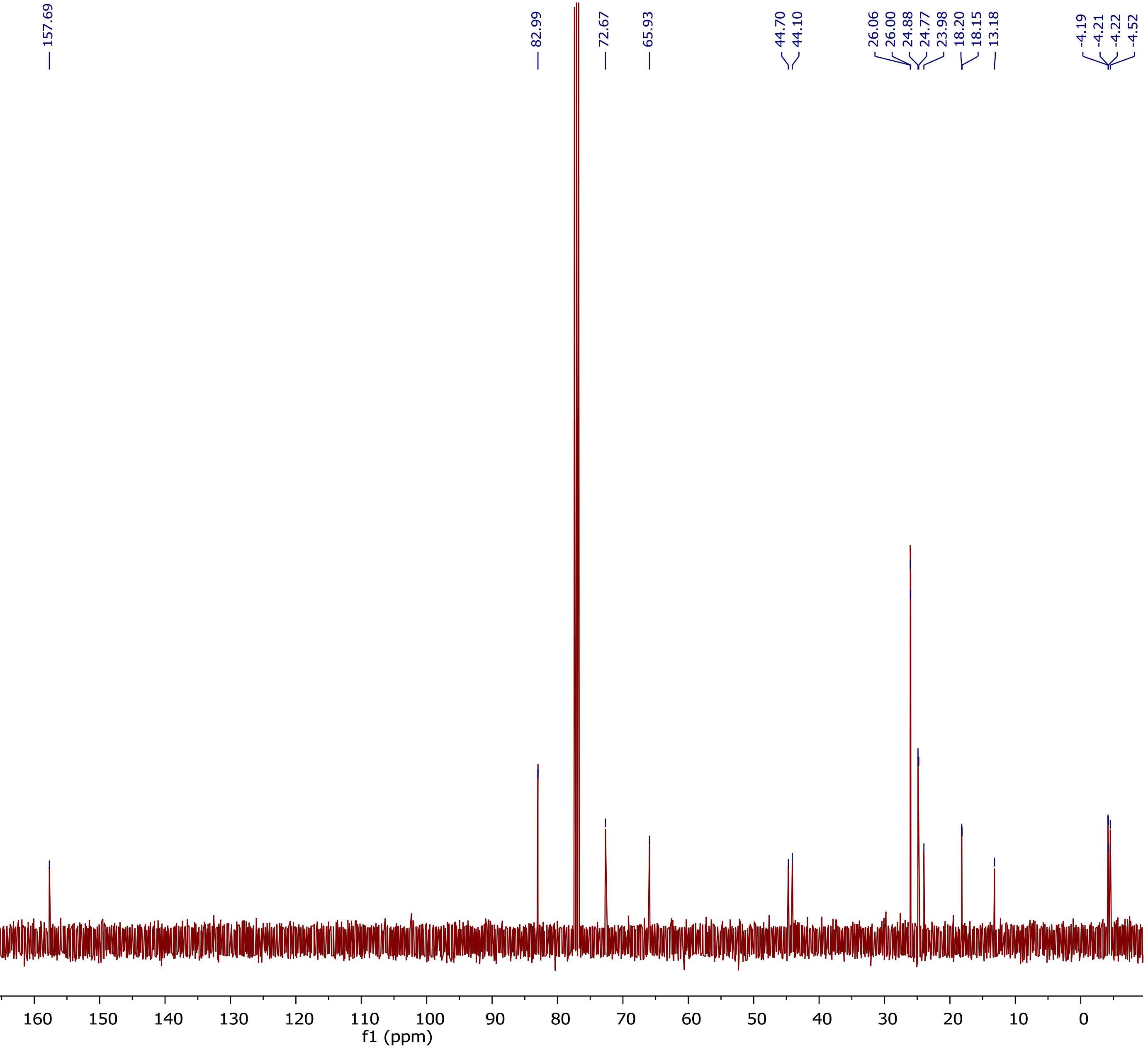
157.69
82.99
72.67
65.93
44.70
44.10
26.06
26.00
24.88
24.77
23.98
18.20
18.15
13.18
-4.19
-4.21
-4.22
-4.52
210 200 190 180 170 160 150 140 130 120 110 100 90 80 70 60 50 40 30 20 10 0
f1 (ppm)

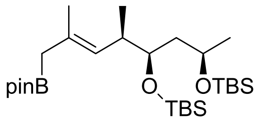
pinB
O
TBS
OTBS

7

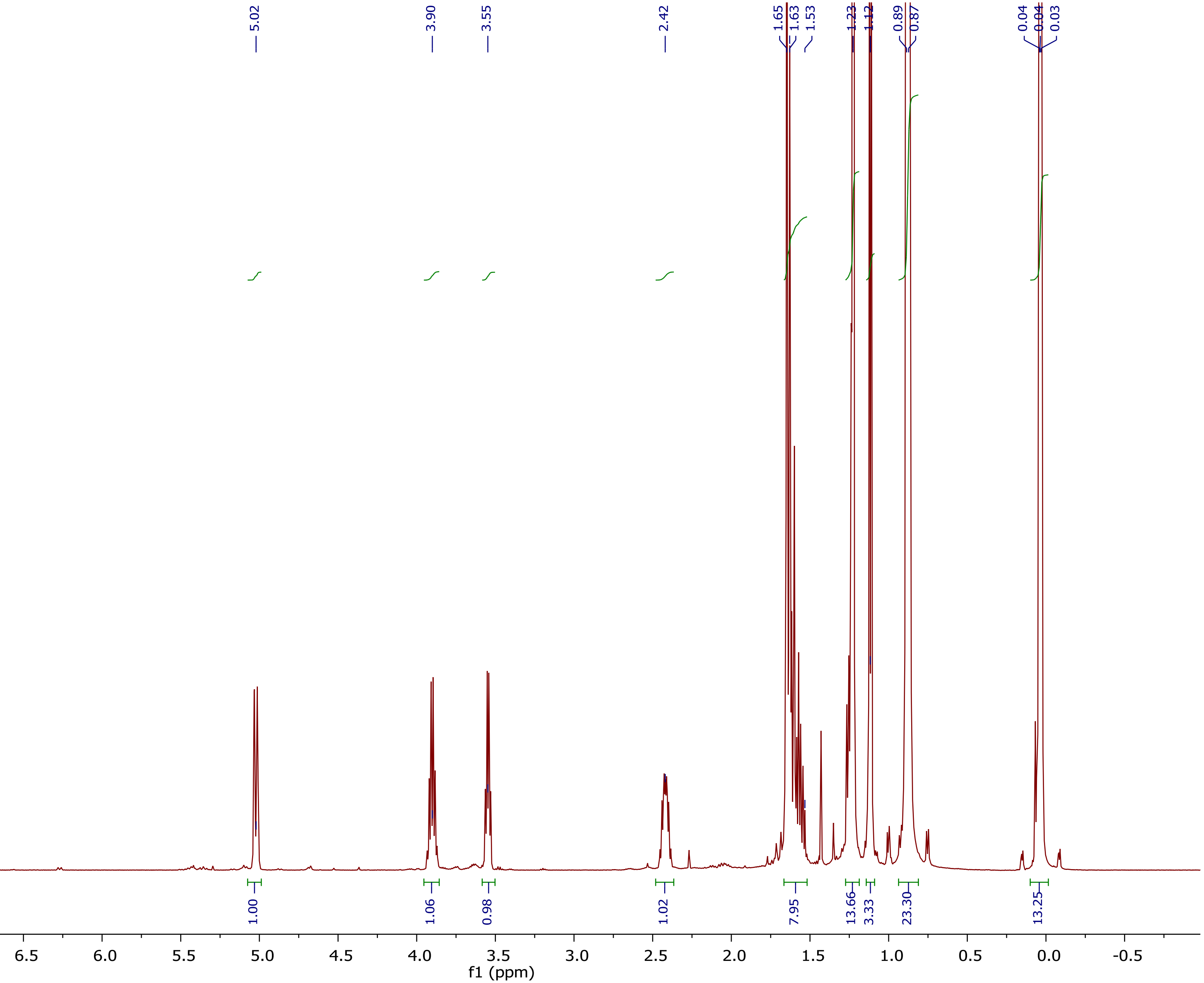
5.02
3.90
3.55
2.42
1.65
1.63
1.53
1.23
1.12
0.89
0.87
0.04
0.04
0.03
1.00
1.06
0.98
1.02
7.95
13.66
3.33
23.30
13.25
7.5
7.0
6.5
6.0
5.5
5.0
4.5
4.0
3.5
3.0
2.5
2.0
1.5
1.0
0.5
0.0
-0.5
f1 (ppm)

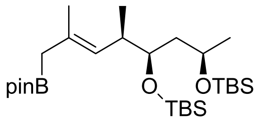

7

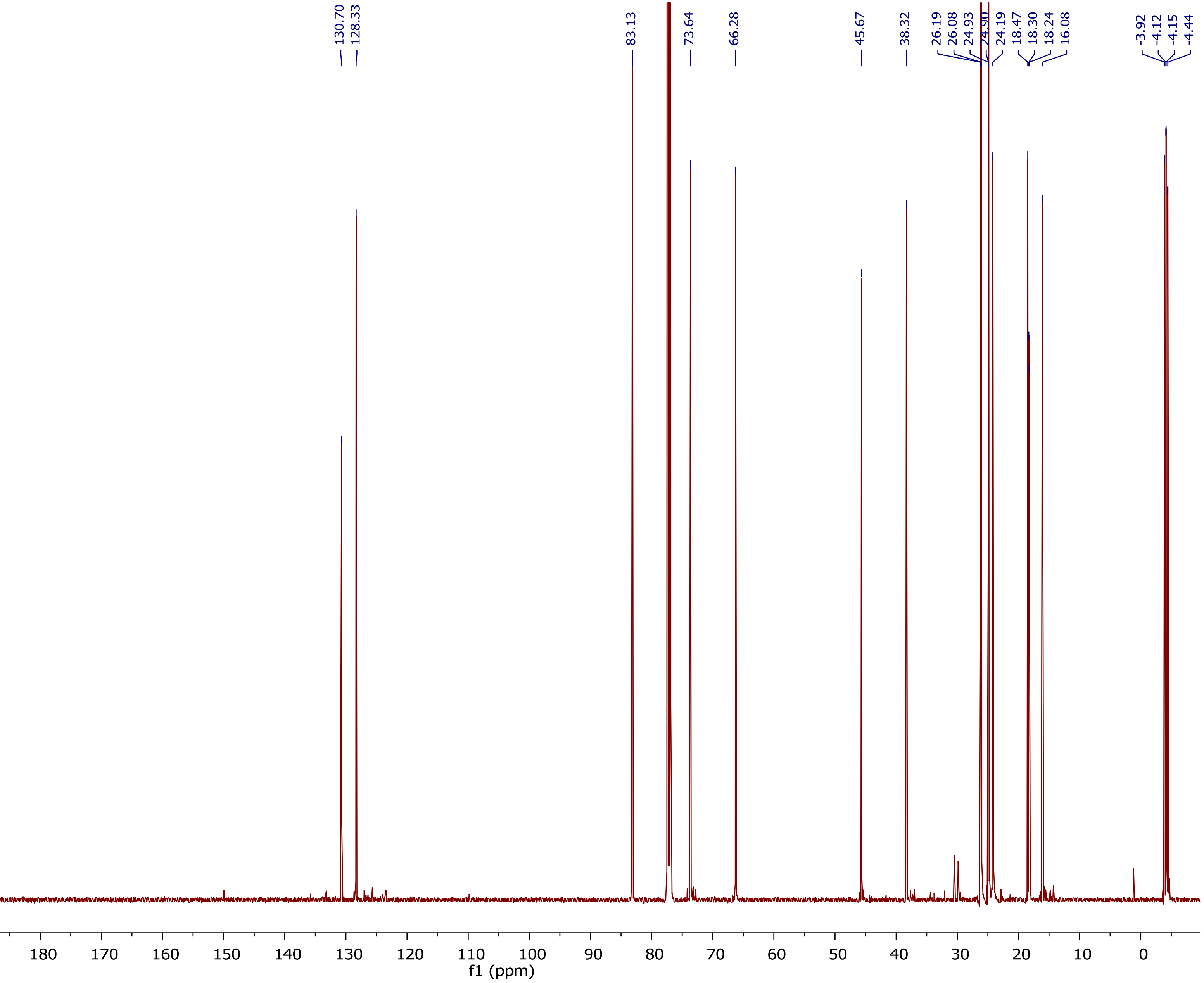

130.70
128.33
83.13
73.64
66.28
45.67
38.32
26.19
26.08
24.93
24.90
24.19
18.47
18.30
18.24
16.08
-3.92
-4.12
-4.15
-4.44
210
200
190
180
170
160
150
140
130
120
110
100
90
80
70
60
50
40
30
20
10
0
f1 (ppm)

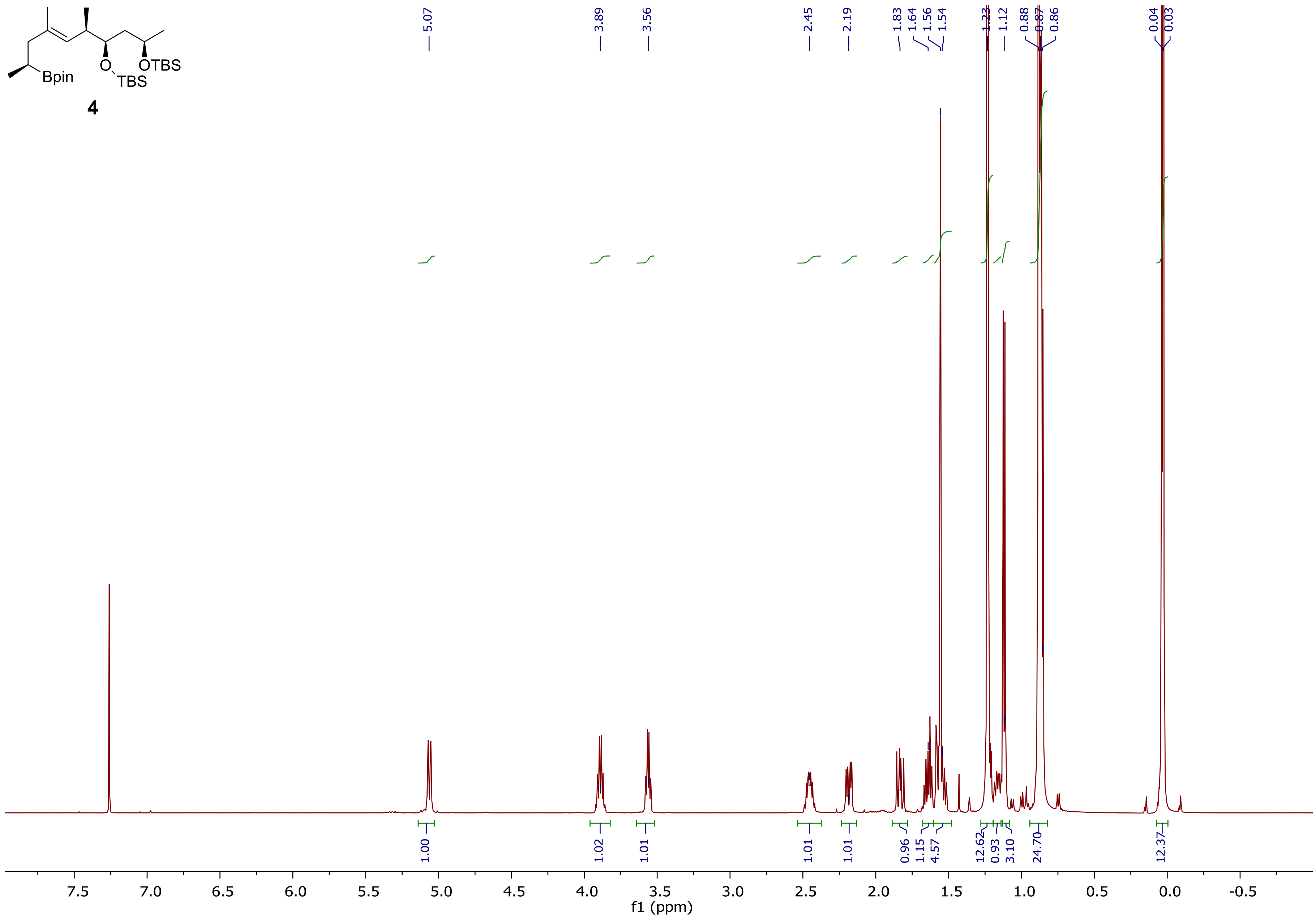
Bpin
O
TBS
OTBS
4
5.07
3.89
3.56
2.45
2.19
1.83
1.64
1.56
1.54
1.23
1.12
0.88
0.87
0.86
0.04
0.03
1.00
1.02
1.01
1.01
1.01
0.96
1.15
4.57
12.62
0.93
3.10
24.70
12.37
7.5
7.0
6.5
6.0
5.5
5.0
4.5
4.0
3.5
3.0
2.5
2.0
1.5
1.0
0.5
0.0
-0.5
f1 (ppm)

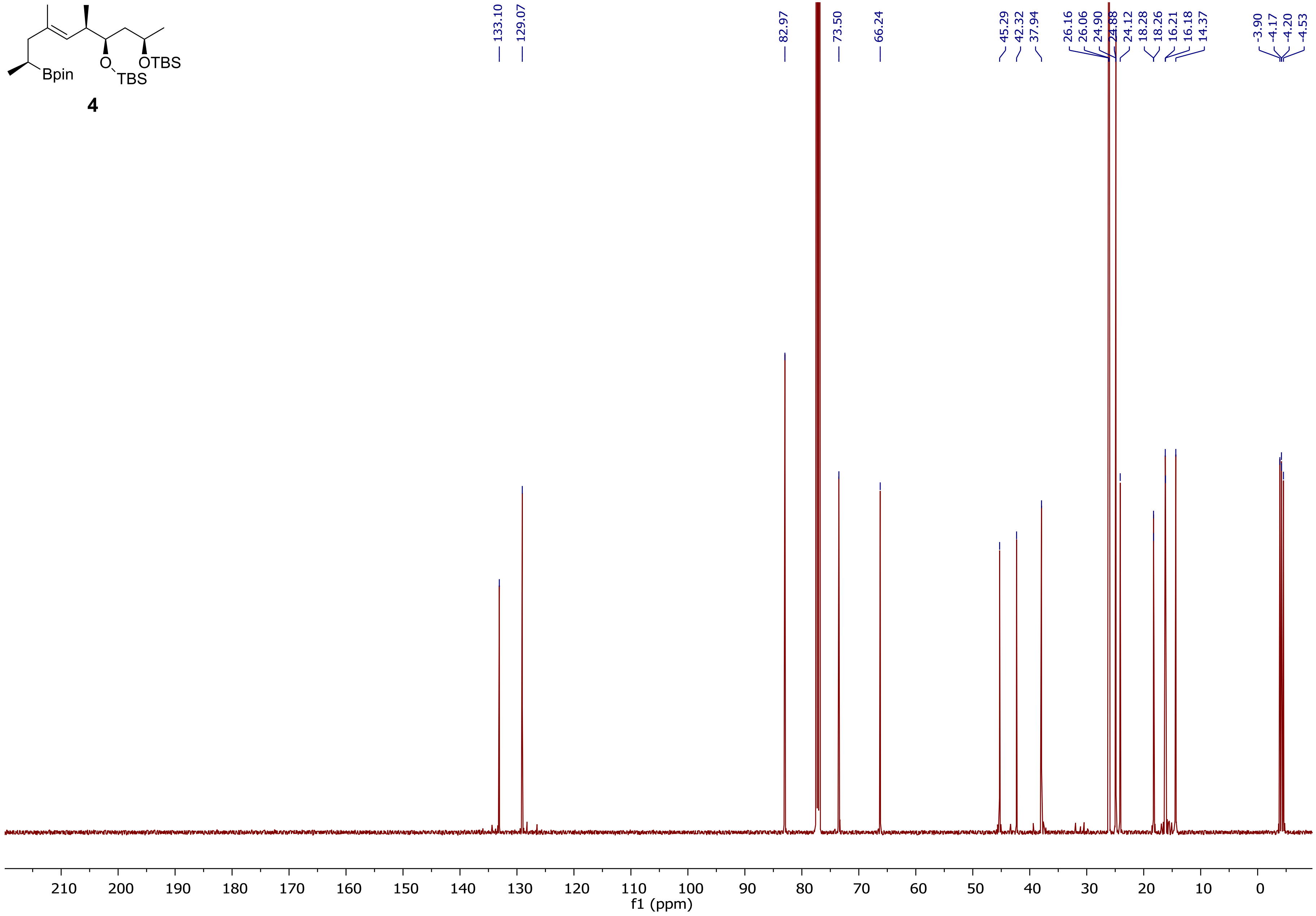
Bpin
O
TBS
OTBS
4
133.10
129.07
82.97
73.50
66.24
45.29
42.32
37.94
26.16
26.06
24.90
24.88
24.12
18.28
18.26
16.21
16.18
14.37
-3.90
-4.17
-4.20
-4.53
210 200 190 180 170 160 150 140 130 120 110 100 90 80 70 60 50 40 30 20 10 0
f1 (ppm)

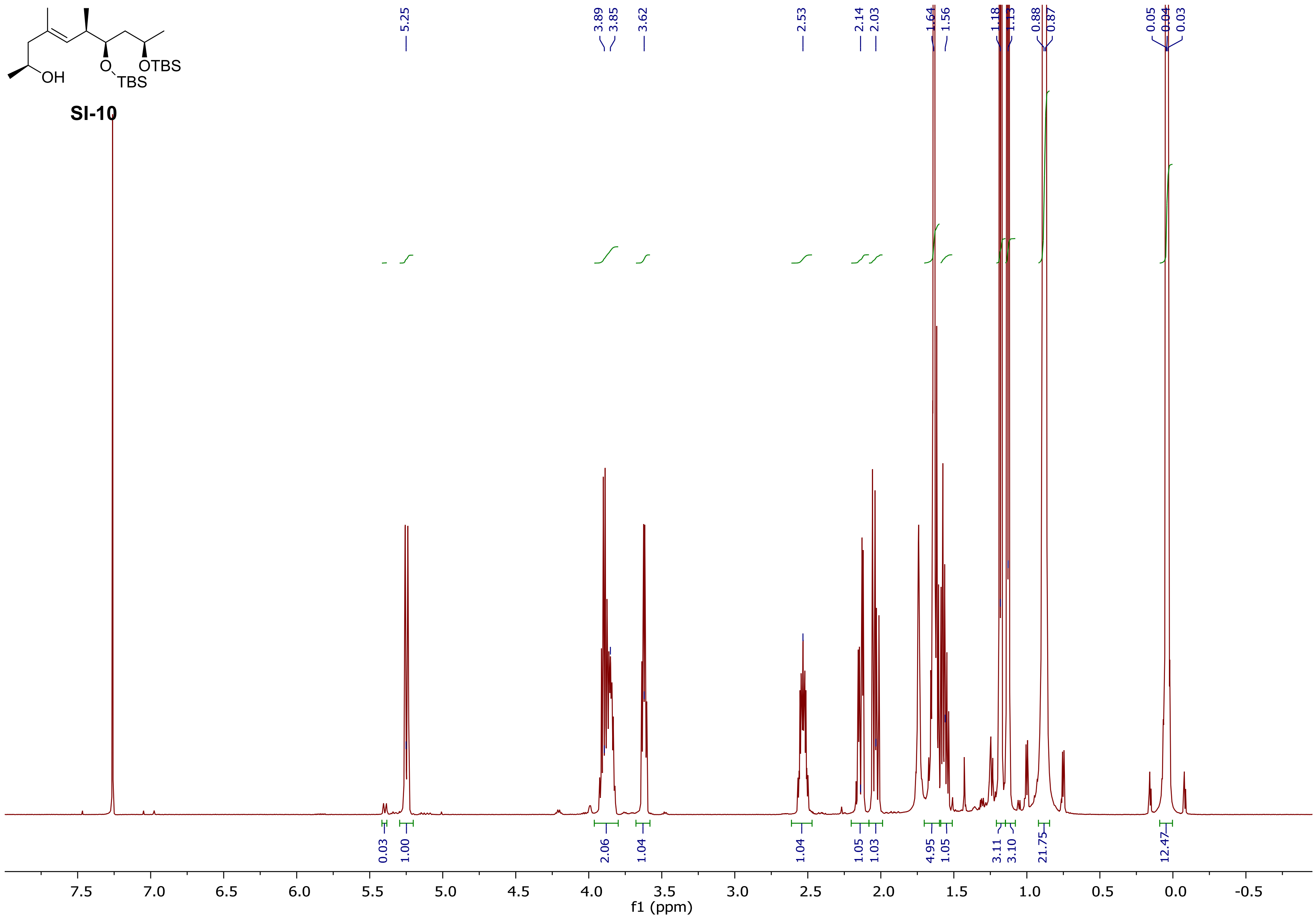
OH
O
TBS
OTBS
SI-10
5.25
3.89
3.85
3.62
2.53
2.14
2.03
1.64
1.56
1.18
1.13
0.88
0.87
0.05
0.04
0.03
0.03
1.00
2.06
1.04
1.04
1.05
1.03
4.95
1.05
3.11
3.10
21.75
12.47
7.5
7.0
6.5
6.0
5.5
5.0
4.5
4.0
3.5
3.0
2.5
2.0
1.5
1.0
0.5
0.0
-0.5
f1 (ppm)

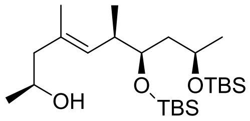
OH
O
TBS
OTBS

SI-10

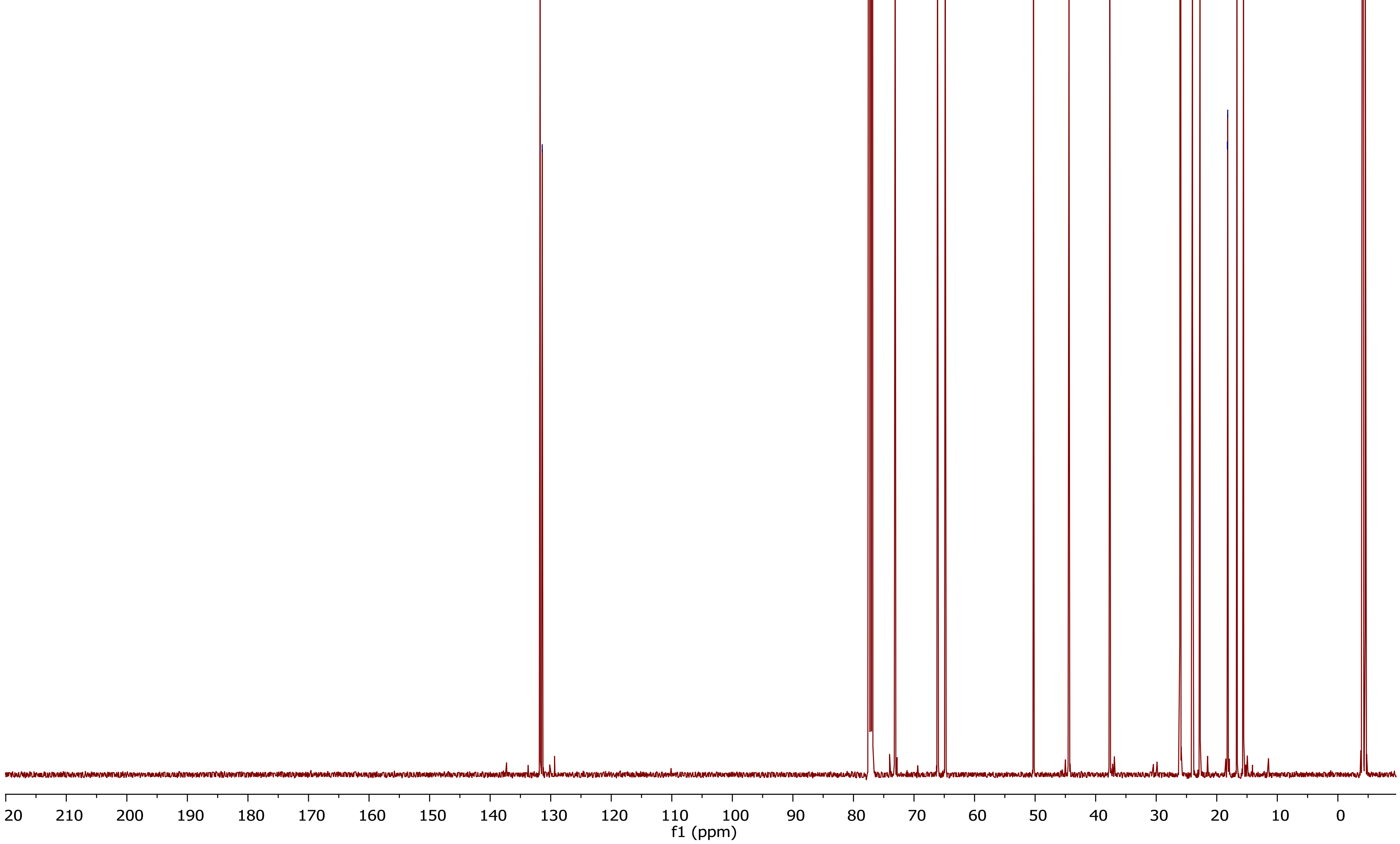
131.74
131.40
73.11
66.12
64.85
50.27
44.41
37.65
26.08
26.01
24.00
22.76
18.25
18.19
16.66
15.58
-4.02
-4.17
-4.19
-4.52
20
210
200
190
180
170
160
150
140
130
120
110
100
90
80
70
60
50
40
30
20
10
0
f1 (ppm)

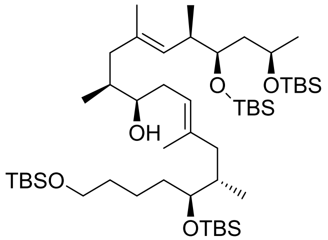
TBSO
OTBS
OH
O
TBS
OTBS

3

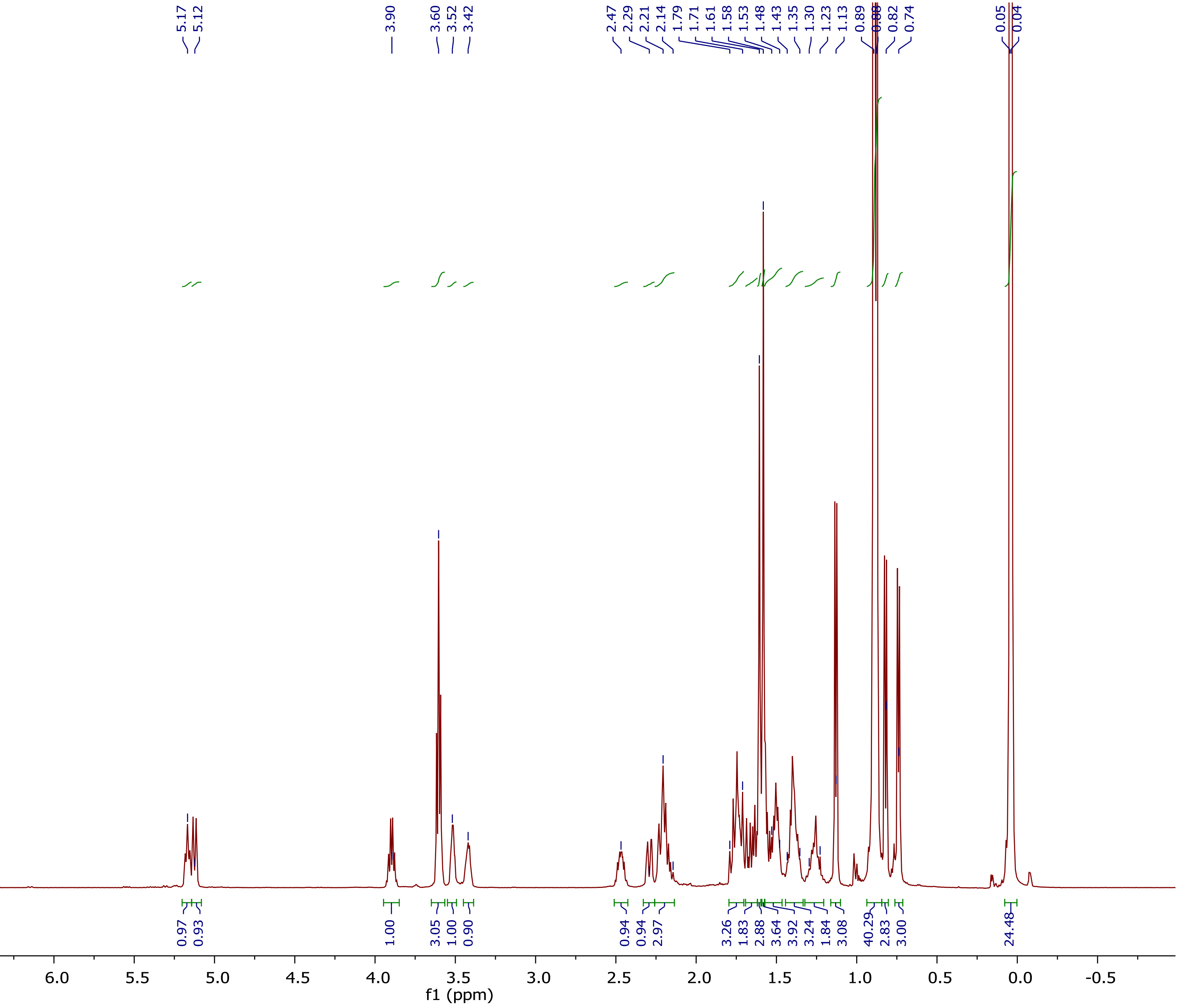
5.17
5.12
3.90
3.60
3.52
3.42
2.47
2.29
2.21
2.14
1.79
1.71
1.61
1.58
1.53
1.48
1.43
1.35
1.30
1.23
1.13
0.89
0.82
0.74
0.05
0.04
0.97
0.93
1.00
3.05
1.00
0.90
0.94
0.94
2.97
3.26
1.83
2.88
3.64
3.92
3.24
1.84
3.08
40.29
2.83
3.00
24.48
7.5
7.0
6.5
6.0
5.5
5.0
4.5
4.0
3.5
3.0
2.5
2.0
1.5
1.0
0.5
0.0
-0.5
f1 (ppm)

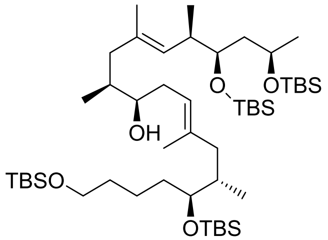
TBSO
OTBS
O
TBS
OH
OTBS

3

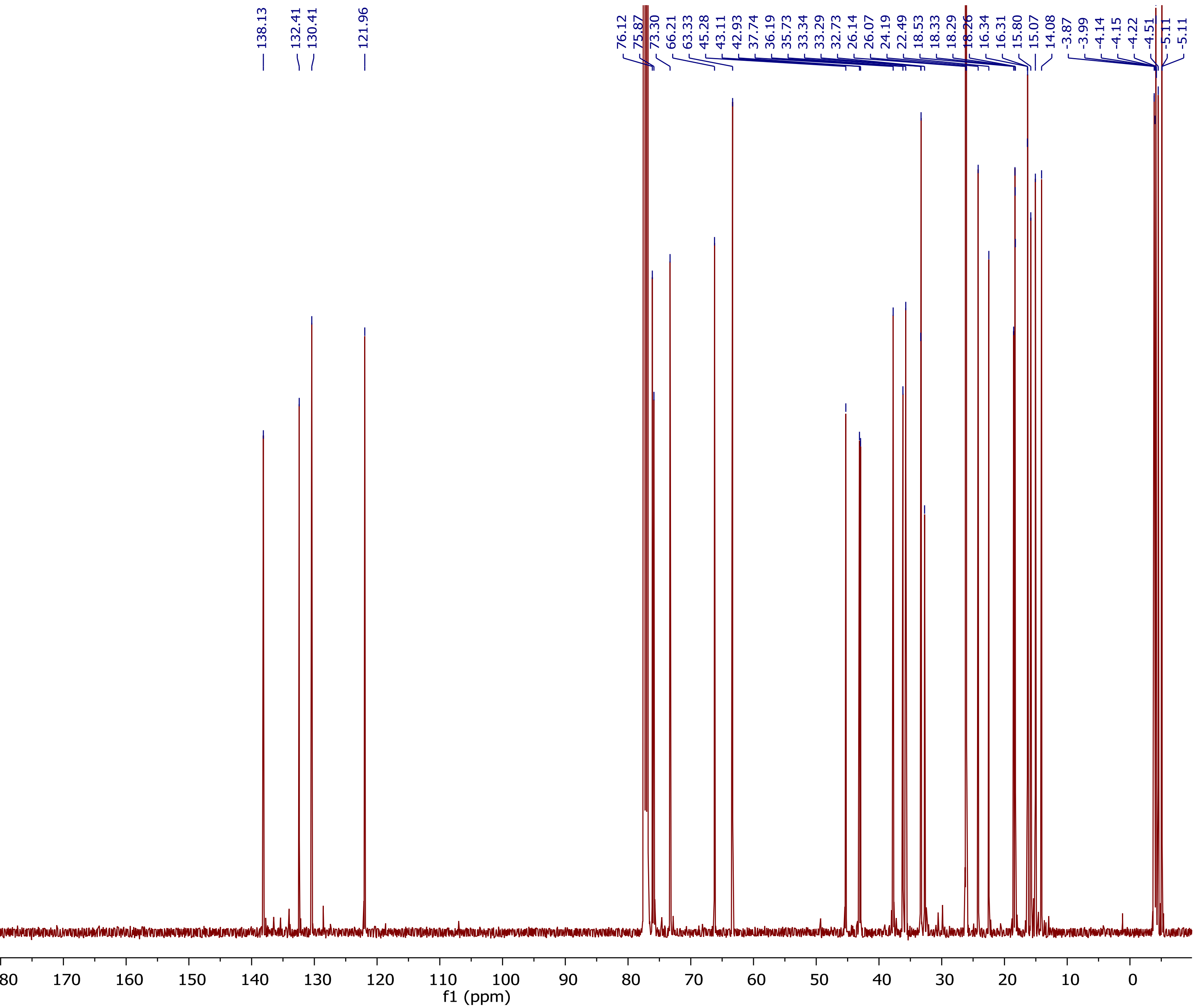
138.13
132.41
130.41
121.96
76.12
75.87
73.30
66.21
63.33
45.28
43.11
42.93
37.74
36.19
35.73
33.34
33.29
32.73
26.14
26.07
24.19
22.49
18.53
18.33
18.29
18.26
16.34
16.31
15.80
15.07
14.08
-3.87
-3.99
-4.14
-4.15
-4.22
-4.51
-5.11
-5.11
210
200
190
180
170
160
150
140
130
120
110
100
90
80
70
60
50
40
30
20
10
0
f1 (ppm)

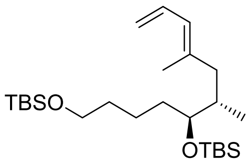

22

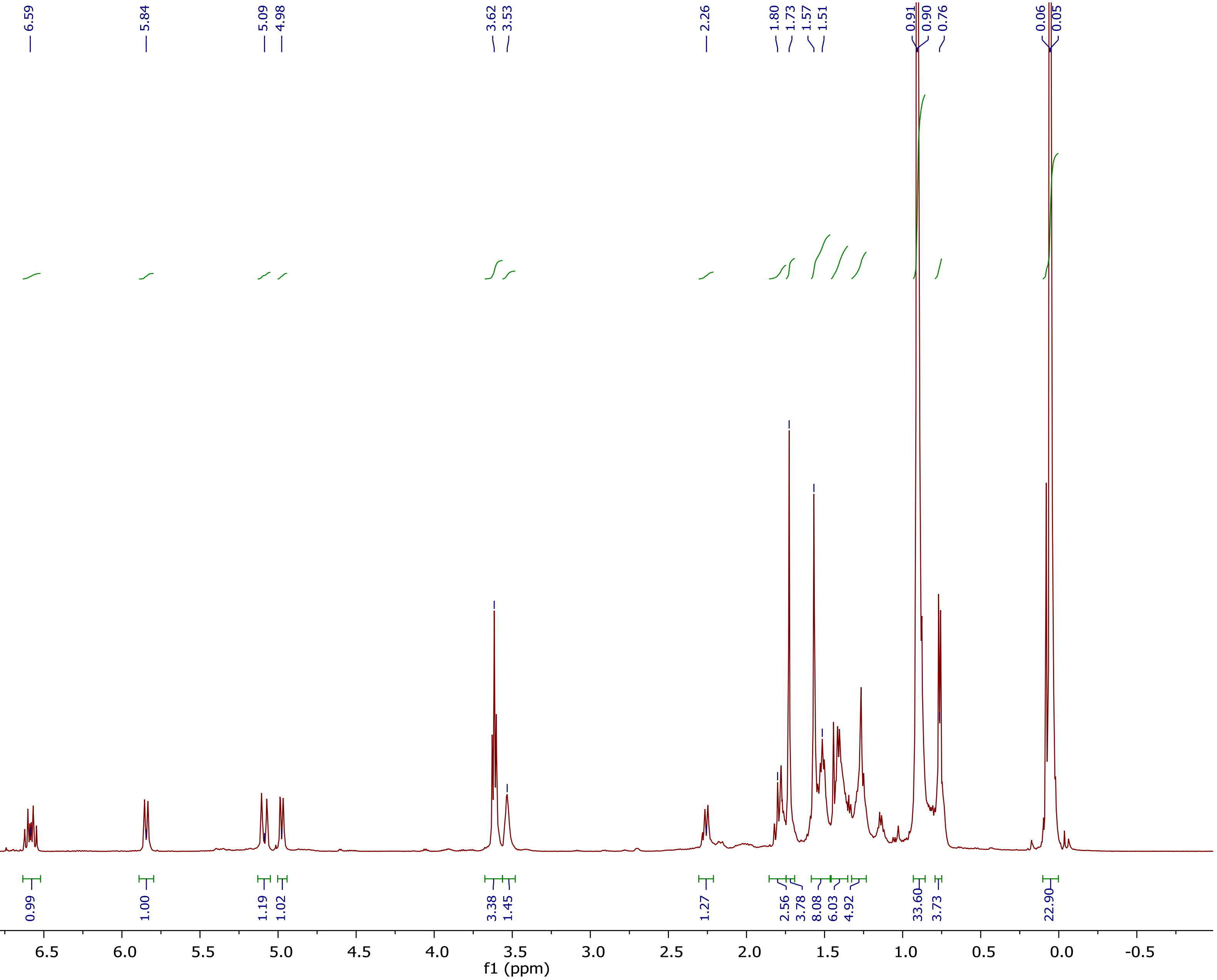

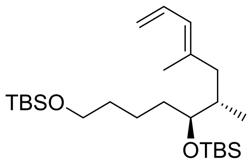
TBSO
OTBS

22

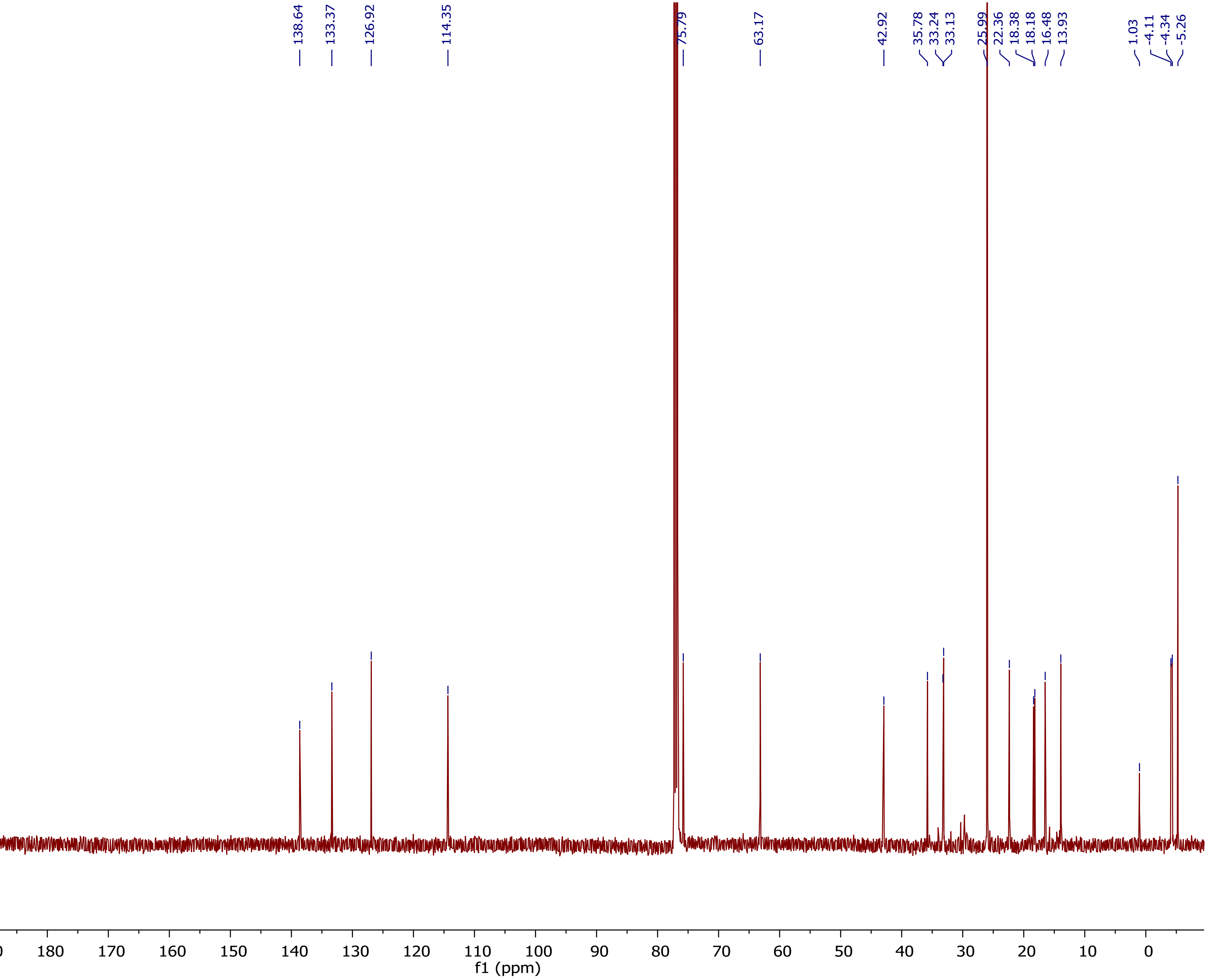
138.64
133.37
126.92
114.35
75.79
63.17
42.92
35.78
33.24
33.13
25.99
22.36
18.38
18.18
16.48
13.93
1.03
-4.11
-4.34
-5.26
210
200
190
180
170
160
150
140
130
120
110
100
90
80
70
60
50
40
30
20
10
0
f1 (ppm)

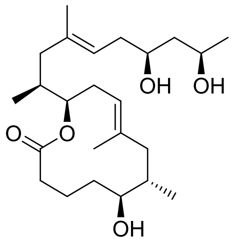
OH
OH
O
O
OH

2

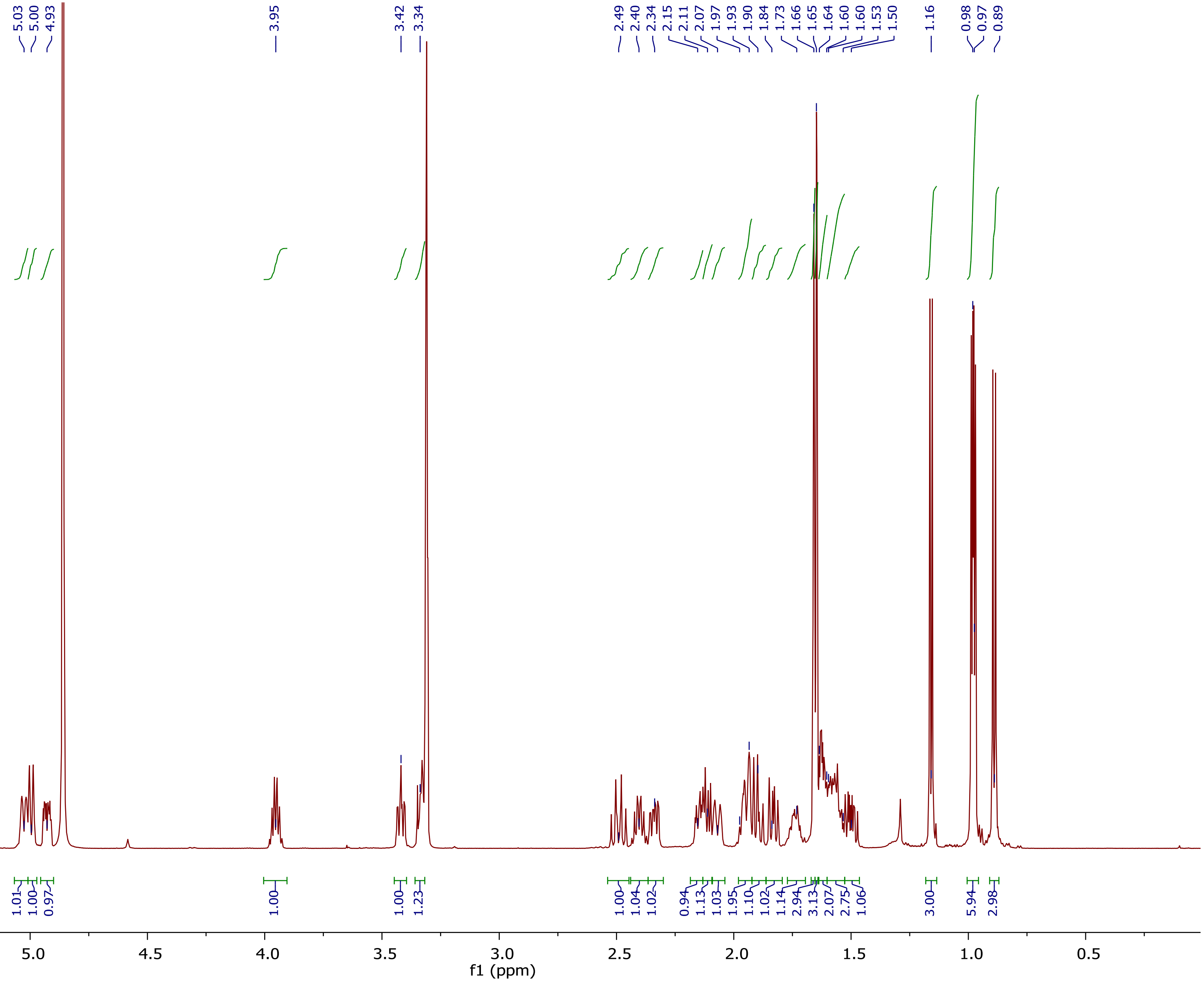
5.03
5.00
4.93
3.95
3.42
3.34
2.49
2.40
2.34
2.15
2.11
2.07
1.97
1.93
1.90
1.84
1.73
1.66
1.65
1.64
1.60
1.60
1.53
1.50
1.16
0.98
0.97
0.89
1.01
1.00
0.97
1.00
1.00
1.23
1.00
1.04
1.02
0.94
1.13
1.03
1.95
1.10
1.02
1.14
2.94
3.13
2.07
2.75
1.06
3.00
5.94
2.98
5.5
5.0
4.5
4.0
3.5
3.0
2.5
2.0
1.5
1.0
0.5
f1 (ppm)

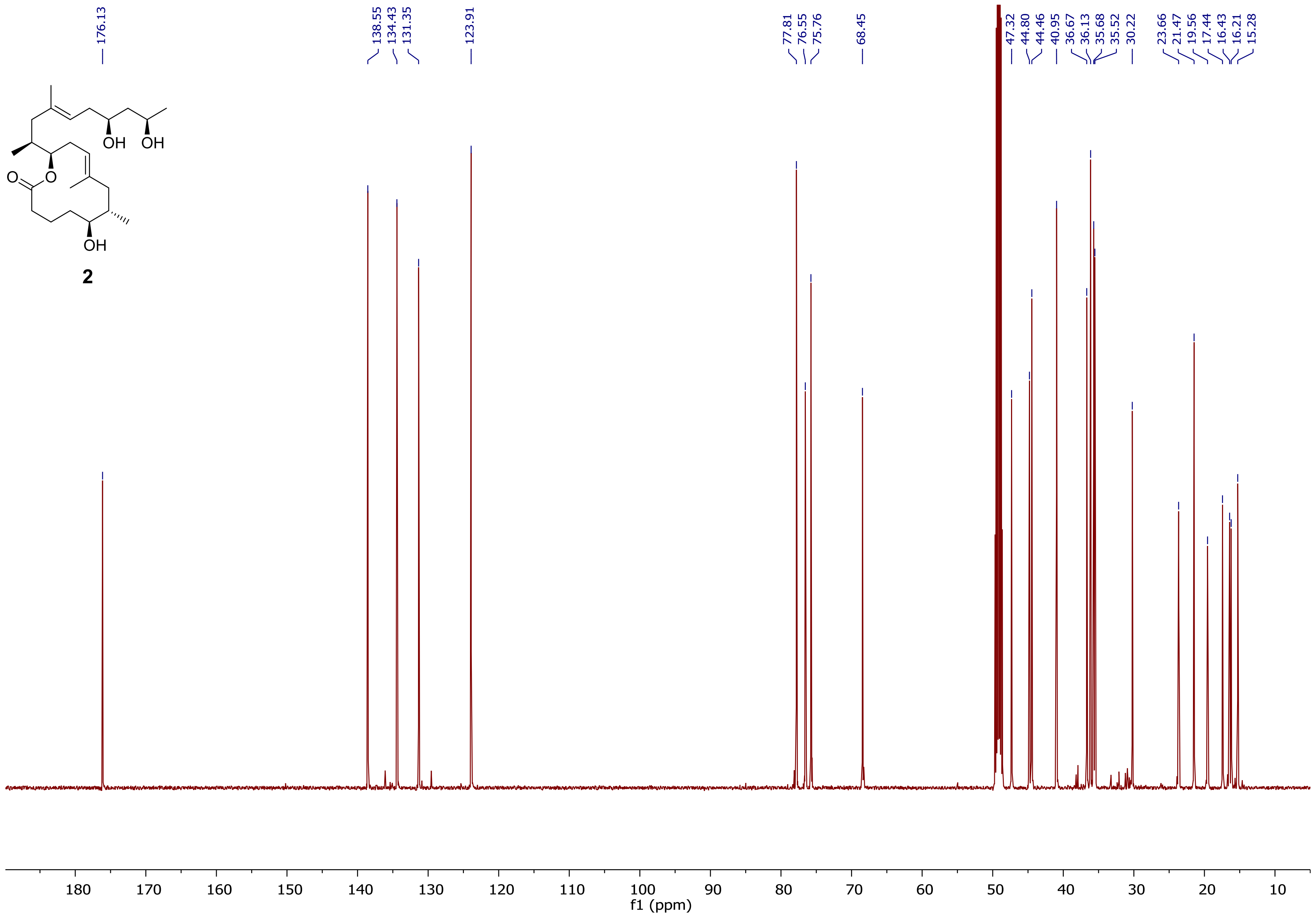
176.13
138.55
134.43
131.35
123.91
77.81
76.55
75.76
68.45
47.32
44.80
44.46
40.95
36.67
36.13
35.68
35.52
30.22
23.66
21.47
19.56
17.44
16.43
16.21
15.28
OH OH
O
O
OH
2
180 170 160 150 140 130 120 110 100 90 80 70 60 50 40 30 20 10
f1 (ppm)
